# Quantitative Tagless Copurification: A Method to Validate and Identify Protein-Protein Interactions* [Fn FN2]

**DOI:** 10.1074/mcp.M115.057117

**Published:** 2016-04-20

**Authors:** Maxim Shatsky, Ming Dong, Haichuan Liu, Lee Lisheng Yang, Megan Choi, Mary E. Singer, Jil T. Geller, Susan J. Fisher, Steven C. Hall, Terry C. Hazen, Steven E. Brenner, Gareth Butland, Jian Jin, H. Ewa Witkowska, John-Marc Chandonia, Mark D. Biggin

**Affiliations:** From the ‡Physical Biosciences Division, Lawrence Berkeley National Laboratory, Berkeley, California 94720;; §Genomics Division, Lawrence Berkeley National Laboratory, Berkeley, California 94720;; ¶OB/GYN Department, University of California San Francisco-Sandler-Moore Mass Spectrometry Core Facility, University of California, San Francisco, California 94143;; ‖Engineering Division, Lawrence Berkeley National Laboratory, Berkeley, California 94720;; **Earth Sciences Division, Lawrence Berkeley National Laboratory, Berkeley, California 94720;; ‡‡Department of Civil and Environmental Engineering, University of Tennessee, Knoxville, Tennessee 37996;; §§Biosciences Division, Oak Ridge National Laboratory, Oak Ridge, Tennessee 37831;; ¶¶Department of Plant and Microbial Biology, University of California, Berkeley, California 94720;; ‖‖Life Sciences Division, Lawrence Berkeley National Laboratory, Berkeley, California 94720

## Abstract

Identifying protein-protein interactions (PPIs) at an acceptable false discovery rate (FDR) is challenging. Previously we identified several hundred PPIs from affinity purification - mass spectrometry (AP-MS) data for the bacteria *Escherichia coli* and *Desulfovibrio vulgaris*. These two interactomes have lower FDRs than any of the nine interactomes proposed previously for bacteria and are more enriched in PPIs validated by other data than the nine earlier interactomes. To more thoroughly determine the accuracy of ours or other interactomes and to discover further PPIs *de novo*, here we present a quantitative tagless method that employs iTRAQ MS to measure the copurification of endogenous proteins through orthogonal chromatography steps. 5273 fractions from a four-step fractionation of a *D. vulgaris* protein extract were assayed, resulting in the detection of 1242 proteins. Protein partners from our *D. vulgaris* and *E. coli* AP-MS interactomes copurify as frequently as pairs belonging to three benchmark data sets of well-characterized PPIs. In contrast, the protein pairs from the nine other bacterial interactomes copurify two- to 20-fold less often. We also identify 200 high confidence *D. vulgaris* PPIs based on tagless copurification and colocalization in the genome. These PPIs are as strongly validated by other data as our AP-MS interactomes and overlap with our AP-MS interactome for *D.vulgaris* within 3% of expectation, once FDRs and false negative rates are taken into account. Finally, we reanalyzed data from two quantitative tagless screens of human cell extracts. We estimate that the novel PPIs reported in these studies have an FDR of at least 85% and find that less than 7% of the novel PPIs identified in each screen overlap. Our results establish that a quantitative tagless method can be used to validate and identify PPIs, but that such data must be analyzed carefully to minimize the FDR.

Proteins interact with each other to form macromolecular complexes in which the activities of each member can be affected by the presence or absence of the other components of the complex (1, 2). Characterizing protein-protein interactions (PPIs)^1^ system-wide will thus greatly aid accurate regulatory and metabolic models of cells.

Two methods have chiefly been used to identify PPIs at high throughput: yeast two hybrid (Y2H) screens and affinity purification—mass spectrometry (AP-MS) (2). The accuracy of the “interactomes” resulting from such screens, however, is a matter of debate (*e.g.* (3–5)). By analyzing AP-MS data with a more stringent approach than used previously, we have previously identified several hundred PPIs for each of the bacteria *Escherichia coli* and *Desulfovibrio vulgaris* (6). These interactomes have significantly lower false discovery rates (FDRs) than nine previously published bacterial Y2H or AP-MS interactomes. In addition, the PPIs in our interactomes are much more frequently detected in independent Y2H or AP-MS experiments, encoded in the same operon, and annotated with the same function than are protein pairs identified in the earlier bacterial screens.

Given the challenge of identifying *bona fide* PPIs, we have developed an additional strategy to validate and detect protein interactomes. Historically, protein complexes were identified individually. A complex was inferred when multiple polypeptides comigrated with an associated enzyme activity through multiple separation steps (*e.g.* (7–10)). Inspired by this classic approach, we have established a “tagless” strategy that detects endogenous complexes isolated from wild-type cells based on the shared elution profiles of polypeptides through multiple chromatographic steps. Like AP-MS and in contrast to Y2H screens, our tagless approach purifies individual protein complexes that can then be characterized further. Unlike AP-MS, though, our method can be scaled to high throughput in any organism because it does not require either genetic manipulation to introduce an affinity tag or the large-scale production of antibodies.

We previously demonstrated the feasibility of the tagless concept in a small-scale study in *E. coli* (11) and in an analysis of outer membrane proteins in *D. vulgaris* (12). We have also fractionated soluble proteins from *D. vulgaris* to purify 14 homomeric and two heteromeric protein complexes >400 kDa and solved the structures of eight of these complexes by electron microscopy (13). We now show that our tagless method can be used in two ways to characterize interactions on a genome-wide scale. First, we show it can be used to compare the accuracy of proposed interactomes by determining the percent of protein pairs in each set that copurify. Second, we demonstrate that when combined with genome location information the tagless strategy can be used to identify PPIs *de novo* with an accuracy comparable to that of our high stringency AP-MS method.

Other variants of the tagless method have been developed (14–19). The one most similar to ours was used in two studies of human cell lines that reported 13,993 and 16,665 PPIs respectively (16, 19). However, our reanalysis of the data from both articles suggest that the majority of the novel PPIs that were not part of the training set of known gold standard PPIs are false positives. We discuss the likely accuracy of previously published interactomes.

## EXPERIMENTAL PROCEDURES

### Cell Culture and Protein Fractionation

A 400 L culture of wild-type *D. vulgaris* was grown aerobically and harvested as described previously (13). Soluble protein extract was prepared from these cells essentially as before (20), except that cells were broken open in an extraction buffer of 25 mm Hepes pH 7.6, 100 mm KCl, 12.5 mm MgCl_2_, 0.1 mm EDTA, 2 mm DTT, 20% glycerol, and 1 mm PMSF. All subsequent separations were performed at 4 °C except for hydrophobic interaction chromatography (HIC), which was run at room temperature. Buffer A contained 25 mm HEPES pH 7.6, 10% (v/v) glycerol, 2 mm DTT, 0.01% (v/v) Nonidet P-40. Buffer A' was identical to Buffer A except that Nonidet P-40 was omitted.

#### 

##### Q-Sepharose Clean-up

Ten grams of soluble protein extract in extraction buffer was loaded onto a 5.0 × 30 cm, 500 ml, Q-Sepharose Fast Flow column (GE Healthcare, Chicago, Il) equilibrated with Buffer A + 50 mm NaCl, and the bound proteins were eluted with Buffer A + 500 mm NaCl. All fractions containing significant amounts of protein were pooled, resulting in a total protein yield of 7 g.

##### Ammonium Sulfate Precipitation

The pooled protein from the Q-Sepharose cleanup step was then fractionated into 6 parts by ammonium sulfate precipitation: 0–38%, 38–48%, 48–53%, 53–57%, 57–63%, and >63% ammonium sulfate saturation.

##### MonoQ Anion Exchange Chromatography (Q-IEC)

Two ammonium sulfate fractions, 38–48% and 57–63%, each containing ∼1 g protein, were resuspended in Buffer A and then exchanged into Buffer A + 50 mm NaCl using a 5.0 × 30 cm, 500 ml, Sephadex G25 (GE Healthcare) column to remove contaminating ammonium sulfate. Each of these two fractions was then loaded onto a separate 3.5 × 10 cm, 96 ml Q-IEC column (GE Healthcare). The Q-IEC columns were pre-equilibrated with Buffer A + 50 mm NaCl and developed with a linear gradient from 50 mm–500 mm NaCl in Buffer A over 25 column volumes at a flow rate of 10 ml/min and fraction size of 24 ml. All of the Q-IEC fractions were analyzed by both native PAGE and SDS-PAGE (supplemental Fig. S1).

##### Hydrophobic Interaction Chromatography (HIC)

Every second or third Q-IEC fraction that contained significant amounts of protein (80–200 mg proteins) were each fractionated by HIC. Each Q-IEC fraction was diluted with an equal volume of Buffer A' + 2 m (NH_4_)_2_SO_4_ and applied to a 5 ml HiTrap Phenyl HP column (GE Healthcare) equilibrated with Buffer A' + 1 m (NH_4_)_2_SO_4_. After washing with 2 column volumes of Buffer A' + 1 m (NH_4_)_2_SO_4_, the column was developed with a linear gradient from 1 m - 0 m (NH_4_)_2_SO_4_ in Buffer A' over 15 column volumes at a flow rate of 1 ml/min and fraction size of 2.5 ml. A total of 29 HIC columns were run.

##### Size Exclusion Chromatography (SEC)

Every other HIC fraction that contained a significant amount of protein (0.4–4.0 mg protein) was fractionated by a 1.6 × 60 cm, 120 ml Superdex 200 column (GE Healthcare) at a flow rate of 0.4 ml/min and fraction size of 2.5 ml. A total of 332 SEC columns were run (supplemental Fig. S2).

### Tryptic Digestion and Labeling With Isobaric Tags for Relative Quantitation (iTRAQ)

#### 

##### 96-well Plate Trypsin Digestion

Our protocol is based on a method originally introduced by Papac *et al.*, for protein N-deglycosylation (21) that was further adopted for protein tryptic digestion and iTRAQ labeling by Basa *et al.* (22). A Multiscreen-IP 0.45 μm 96 well plate (Millipore, Billerica, MA, MAIPN4510) was used with a multifold system apparatus (Millipore) in which protein samples, buffers and reagents are filtered through polyvinyl difluoride (PVDF) membranes. The PVDF membranes in each well were first wetted with 100 μl ethanol for 10 s, then rinsed three times with 250 μl MilliQ water and once with 50 μl 6 m Guanidine/HCl. 1 ml of each column fraction containing 0.2–40 μg of protein was denatured by adding 1 ml of 6 m Guanidine/HCl and then the proteins were bound to the PVDF membrane and the denaturant removed by application of the vacuum. The membrane bound proteins were reduced by incubating 50 μl of Tris-(2-carboxyethyl)-phosphine (TCEP) (1 mg/ml) in 6 m Guanidine/HCl with the membrane at 37 °C for 1 h. The reducing solution was removed and the membrane was rinsed three times with 250 μl of MilliQ water. The protein was then alkylated by addition of 50 μl 25 mm iodoacetamide to each well for 30 min in the dark at room temperature. Next the membrane was blocked by incubation with 100 μl of 1% polyvinylpyrrilidone (PVP)-360 solution at room temperature for 30 min. The membranes were rinsed with 250 μl MilliQ water three times. Trypsin digestion was carried out by incubating the membrane in each well with 40 μl trypsin (20 ng/μl in 0.5 m triethylammonium bicarbonate (TEAB), Promega, Fitchberg, WI, Sequencing Grade) for 4 h at 37 °C in a humid incubator. The tryptic peptides were eluted from the membranes into a 96-well collection plate using the vacuum. The membranes were washed twice with 10 μl ethanol, the washes being combined with the eluted peptide solutions by centrifugation of the Multiscreen-IP filter plate/collection plate at 2000 rpm using a Beckman J6-MC centrifuge.

##### iTRAQ Derivatization

4-plex (114–117) or 8-plex iTRAQ reagents (113–119, 121) (AB Sciex, Redwood City, CA) were prepared by adding 70 μl of ethanol to each vial to give a total volume of 90 μl. Large scale derivatization reactions were carried out by mixing these 90 μl aliquots with the tryptic peptides eluted from a single fraction and incubating the two at room temperature for 1 h. We also developed a protocol that used 1/8th of the iTRAQ reagent but gave the same quantification accuracy and reproducibility. In this case, 9 μl of the ethanol/iTRAQ reagent solution was mixed with 6.5 μl of digested peptides and incubated as above.

##### Forming iTRAQ Multiplexes

iTRAQ derivatized peptides from a series of column fractions were pooled into multiplexes. 8-plex labeling was preferred as it allows more fractions to be analyzed per unit time, 4-plex labeling only being used prior to the availability of 8-plex reagents. An 8-plex would have fractions derivatized with iTRAQ labels 113–119 and 121. To allow protein elution profiles to be quantitated across all selected fractions from a single column, one “joint” fraction was labeled twice with a common iTRAQ label (*e.g.* 113) that was used in two otherwise nonoverlapping multiplexes.

SEC iTRAQ derivatized fractions from the same Q-IEC fraction were pooled according to two different schemes to generate elution profiles for both SEC and HIC columns (Fig. 2*A*). To determine protein elution profiles along the SEC dimension, several distinct multiplexes were formed that together covered 19 consecutive fractions from the same SEC column. The iTRAQ derivatized fractions were generally pooled to form three multiplexes of 8, 8, and 5 fractions respectively, the 8th and 15th fractions being common to adjacent multiplexes. In early experiments when 4-plexes were employed, however, more multiplexes were required to cover a single column. To determine protein elution profiles along a single HIC dimension, 12 iTRAQ derivatized fractions were pooled from the different sizing columns run using the protein that eluted from that HIC column. Derivatized digested proteins that eluted from SEC columns at the same retention time (*i.e.* size) were pooled into pairs of multiplexes that usually contained 8 and 5 fractions respectively. This process was repeated for groups of similarly eluting fractions for other retention times (Fig. 2B), yielding ∼10 sets of multiplex pairs that measure elution across a single HIC column. The combined iTRAQ derivatized fractions for each multiplex were speed vacuumed down to ∼20 μl, acidified with 0.1% trifluoroacetic acid (TFA), zip-tipped (C18 Millipore) and submitted to MALDI LC MS/MS analysis.

### Mass Spectrometry and Identification of Proteins

#### 

##### Reversed Phase HPLC Peptide Fractionation

iTRAQ-labeled peptide mixtures were separated by reversed phase chromatography using an Ultimate 3000 dual column HPLC system (Dionex, Sunnyvale, CA) that was set up in a parallel configuration and equipped with a pair of reversed phase LC Packings/Dionex Monolithic PepSwift-DVB trap and analytical columns (200 μm × 1 cm and 200 μm × 5 cm, respectively). The LC system was operated in a swinging fashion to allow for a simultaneous peptide fractionation and column equilibration using an active and a resting column, respectively. A linear LC gradient (flow rate of 2.5 μl/min) was used, in which the percentage of mobile phase B [80% acetonitrile, 0.05% TFA in water] in mobile phase A [0.05% TFA in water] was increased from 0% at 5 min to 60% at 19 min. Starting from 9.7 min, the LC eluates were mixed with MALDI matrix [5 mg/ml α-cyano-4-hydroxycinnamic acid (CHCA) in 80% acetonitrile/0.05% TFA], containing 10 mm ammonium phosphate and 20 fmol/μl of [Glu^1^]-fibrinopeptide B (Glu-Fib) as internal calibration standard and spotted onto a blank MALDI plate (AB Sciex) using a SunChrom Fraction Collector/Spotter (Sunchrom, Friedrichsdorf, Germany). Each sample was fractionated into 129 fractions over an 8-min collection time, with a frequency of 3.66 s per spot. Typically, fractions from 10–12 LC runs were placed on a MALDI plate.

##### MALDI Mass Spectrometry

The majority of analyses were performed using a 4800 MALDI TOF/TOF mass spectrometer (AB Sciex) operated using 4000 Series Explorer software (version 3.5.28193; build 1011, AB Sciex). External calibration based on Plate Model software (AB Sciex) was applied. Internal one-point calibration using the monoisotopic mass of the spiked Glu-Fib (m/z 1570.677) as a reference was performed for all spectra that met the preset internal standard data quality criteria (minimum accuracy of 0.2 Da and signal-to-noise (S/N) of 50). The total number of shots per spectrum was 800–1500 for MS and 1500–4000 for MS/MS, the latter using the vendor's supplied “stop conditions” software, which automatically stopped data acquisition once all the specified criteria were reached (an estimated S/N of 60 for an accumulated spectrum, and a minimum of 4 peaks above the S/N threshold at m/z >200, excluding a 100 m/z range directly below the precursor mass). The fixed laser intensity of 3800–4500 and 4700–5500 was used in MS and MS/MS modes, respectively. Collision cell was floated at 1 kV and ambient air was used as a collision gas; gauge read the pressure of ∼5E-07 bar. Using the Interpretation Method algorithm for the 4000 Series Explorer software, the 12 most abundant peaks per MS spectrum (*i.e.* per spot) were automatically selected for MS/MS and fragmented in order of diminishing precursor intensity. Trypsin autolysis peaks were excluded from MS/MS analysis. A small portion of the data were acquired using AB Sciex 5800 TOF/TOF mass spectrometer while employing an iterative MS/MS acquisition routine, as described elsewhere (23).

##### Identifying Proteins and Quantitating Their Abundance from MS Data

The AB Sciex search engine ProteinPilot™ v. 3.0 and 4.0 with the Paragon™ Method algorithm (24) was employed for protein identification and calculation of relative protein abundances. The ProteinPilot “Add TOF/TOF Data” module was used to extract raw MS data stored in an Oracle database for direct submission to a search engine. Early in the project a custom database containing *D vulgaris* proteins, seven protein standards and commonly encountered contaminants (a total of 3688 entries) was employed. The subsequent, majority of analyses (∼88%) utilized an extended database (a total of 51,283 entries) that included 6-frame translated products of the *D. vulgaris* genome. The following settings for the Paragon Method were utilized: iTRAQ 8-plex or 4-plex (peptide labeled) for “Sample Type”; iodoacetamide for “Cys Alkylation”; trypsin for “Digestion”; 4800 for “Instrument”; none for “Species”; and thorough ID for “Search Effort”. None of the options for “Special Factors” and “ID Focus” were selected. “Detected Protein Threshold” was set to 0.47 (66.0%). The presence of at least one peptide matched with a confidence of 95% was used as a threshold for considering a protein for further analysis. Competitor protein identifications based on same evidence (spectra) explained by alternate hypotheses of the same confidence were included (supplemental Data set S1). After subsequent filtering described below, however, all proteins present in pairs that co-occur with CC values ≥0.85 or are part the 200 high confidence PPIs were detected by at least one peptide with a confidence of 99% and were ranked as primary identifications.

The average relative abundance of each polypeptide was calculated on the basis of relative ratio values of constituent peptides using default settings of a ProteinPilot algorithm that employs stringent criteria of eligibility for inclusion into a data pool. Specifically, the following data were excluded from quantitation: (1) peptides matched with confidence < 15%); (2) peptides that could be matched to more than one protein with an Unused ProtScore of at least 1.3; (3) spectra for which the alternate peptide hypothesis had at least some minimal confidence (>1%) (4) peptides with low intensity signals (sum of the S/N for all the reagent pairs is < 9); (5) peptides with partial iTRAQ modifications; (6) Peptides with a combined feature probability < 30%, *e.g.* semitryptic peptides, peptides with low probability modifications and peptides with large delta masses. Neither bias correction nor background subtraction options were employed. The ProteinPilot generated relative abundances were then normalized for each polypeptide by arbitrarily assigning a value of 1 to the fraction in which the polypeptide had the highest abundance within a multiplex and recalculating its relative abundances in all other fractions using the apex-associated iTRAQ reagent as a denominator.

Before using these mass spectrometry data to validate and identify PPIs ribosomal proteins and abundant chaperonins (DVU0811, DnaK; DVU0812, GrpE; DVU1976, GroEL; DVU1977, GroES) were removed because these highly abundant proteins had been shown to lead to many potential false positives in AP-MS data (6) and because the RNA component of the ribosome makes it atypical. After this data filtering, 1,242 unique proteins remained in the data set (supplemental Data set S2).

### Other Bacterial Interactome Data

The PPIs and reciprocally confirmed PPIs for nine bacterial Y2H and AP-MS interactomes (25–33) were derived as previously (6). Homologs and interologs between species were determined as before, as was the percent overlap between different interactomes (6). Three benchmark sets of well characterized PPIs were defined for the EcoCyc protein complexes and for reciprocally confirmed bait-prey, prey-bait pairs from Y2H and AP-MS screens as previously (6).

### Validating Protein Interactomes

Co-occurring protein pairs in the tagless data set were defined as pairs where both proteins were detected in the fractions of the same iTRAQ multiplex (supplemental Data set S3). To quantitate the similarity of elution profiles, Pearson cross-correlation values (CC values) were computed for each multiplex for each pair of proteins that were confidently detected and for which iTRAQ raw intensity values were ≥0.01 for at least one of the proteins in ≥3 fractions. For each pair, the maximum CC scores for the pair in the SEC and separately in the HIC dimensions were determined and used in all subsequent analyses (supplemental Data set S3). Fig. 3 shows the distributions of these maximum CC values for the SEC and HIC dimensions for sets of protein pairs expected to interact and pairs expected not to interact.

The enrichment of co-occurring pairs with high CC values in sets of PPIs from different species was determined as shown in supplemental Table S1. The fraction of protein pairs that have CC values ≥0.85 in both HIC and SEC dimensions was normalized by the fraction of all interologs for a species that have CC values ≥0.85 in both HIC and SEC dimensions, irrespective of whether there is any evidence these pairs interact (supplemental Table S1). This normalization removes small variations in the proportion of conserved protein pairs between species that tend to be highly correlated, which likely reflects differences in abundances of conserved *versus* all proteins. These normalized values are those referred to as “PPI fold enrichment” in the Results section (Fig. 4).

### Identifying PPIs de novo

#### 

##### Gold Standards

Curated gold standard sets of interacting and noninteracting pairs of proteins used previously to identify PPIs from AP-MS data were employed (6). Of the 536 gold standard positive pairs, 57 co-occurred in at least one multiplex in our data set. Of the 27,542 gold standard negative pairs, 1068 co-occurred in the same multiplex. The co-occurring gold standard pairs present in the tagless data set are indicated in supplemental Data set S3.

##### Features to Distinguish Bona Fide PPIs from Noninteracting Protein Pairs

Eight features (scoring functions) for each co-occurring pair of proteins were defined to distinguish *bona fide* PPIs from pairs that do not interact.

(1) Maximal Pearson correlation coefficient (CC) from multiplexes in the SEC dimension, as defined above. These scores range from −1 to +1. If the two proteins were never observed in the same SEC multiplex under the conditions of data dependent precursor ion selection used in the study, a score of −1 was assigned.
(2) Maximal Pearson correlation coefficient (CC) over the HIC dimension, calculated as for (1).
(3) Dice's coefficient for comigration of two proteins over all multiplexes. The number of times two proteins have been observed together in a multiplex divided by the sum of individual observances of each protein in all the multiplexes (34). This feature helps to resolve the problem of “frequent fliers,” which are either proteins that tend to bind nonspecifically to many other proteins or highly abundant proteins detected in many fractions. For frequent fliers this value is close to zero, whereas for proteins that form specific interactions the value is higher.
(4) Peptide ratio. The number of unique peptide sequences detected by MS provides an approximation of protein abundance. We expect that components of stable protein complexes might be more abundant when they copurify with other members of the complex. For each protein in a given multiplex we compute a ratio between the number of unique peptides observed and the maximal number of peptides observed for the protein across all multiplexes. For a co-occurring pair in a given multiplex, we assign the score of the smaller of the peptide ratios calculated for the two proteins. Finally, for each pair of proteins, we assign the score of the maximal value for the pair over all multiplexes.
(5) Minimal number of proteins. Some regions of fractional space are more populated than others. The presence of a higher number of proteins in a fraction leads, by chance, to more highly correlated pairs. We expect less dense regions of fractional space to contain fewer false positives. For each pair of proteins we assign a score as the number of proteins in the multiplex with the fewest total number of proteins in which the two proteins co-eluted with a CC score of at least 0.85.
(6) STRING - Neighborhood. A feature from the STRING database (35) that reflects how frequently in bacterial species the two genes appear nearby on a chromosome.
(7) STRING - Co-occurrence. A feature from the STRING database that reflects how frequently two genes co-appear (anywhere) in a genome across bacterial species.
(8) STRING - Fusion. A feature from the STRING database that reflects how frequently a gene fusion event happens between the two genes across bacterial species.

Distributions of scores for all eight features on our gold standard sets are shown in supplemental Figs. S6–S13 and the values given for each co-occurring pair in supplemental Data set S3.

##### Predicting PPIs

We trained two separate logistic regression classifiers to predict PPIs, using the gold standard sets. One classifier used only the first five features, and the second logistic regression used all eight. The set of predictions from the first classifier is referred to as the “MS-only” set and predictions from the second classifier are referred to as the “MS+STRING” set.

We tested the performance of both classifiers using a cross-validation procedure optimized for our specific problem in which individual interaction pairs cannot be considered as independent measurements because some may share the same proteins (36). At each iteration of cross-validation, all proteins from a single operon were selected and all their interactions (both within and outside the operon) were used for validation and the rest were used for training. We call this procedure one-operon-out cross validation. We first applied a threshold that gives a 20% FDR based on the cross-validation tests. This identified 201 MS-only PPIs and 300 MS+STRING PPIs. A subset of 200 these PPIs were then classified as high confidence PPIs based on high logistic regression score and being more highly enriched in multiple PPI quality metrics, see “Results” and supplemental Data set S4. The distributions of scores for all five MS-only regression features for the 200 high confidence tagless PPIs are shown in supplemental Fig. S14. These distributions closely resemble those for the same features measured on the gold standard positive proteins (compare supplemental Figs. S14 with supplemental Figs. S6–S10). Thus the logistic regression that included the additional three STRING features did not rely solely on these features, but also strongly relied on the MS data to determine likely PPIs.

### Experimental Design and Statistical Rational

This project determines high confidence PPIs using a logistic regression that combines multiple different features from the mass spectrometry data, described above. For this reason, no single aspect of the mass spectrometry data, such as reproducibility between technical or biological replicas, provides the most telling measure of accuracy. Instead, the fundamental criteria for judging the accuracy of our high confidence PPIs are the FDRs calculated using gold standard and gold negative protein pairs, see above, and the additional quality metrics shown in the Results section. Our analysis indicates that the PPIs in our high confidence interactome are comparable in accuracy to those in three benchmark sets of validated PPIs: the EcoCyc data set and AP-MS and Y2H PPIs that have been reciprocally confirmed in biological replicas as bait prey and prey bait pairs. In contrast, by the same suite of criteria, nine previously proposed bacterial interactomes are much less accurate.

That said the reproducibility of our tagless assay is revealed in two further ways. First, *bona fide* protein pairs copurify in both of two different, orthogonal chromatography separations much more frequently than randomly chosen protein pairs (Figs. 3 and 4). Second, three of the features used in the logistic regression (Dice's coefficient, maximum CC SEC, and maximum CC HIC) measure copurification in separate events. Strong scores in multiple of these features are highly favored by the logistic regression (supplemental Data set S4).

### Estimating a False-Negative Rate and the Overlap Expected Between D. vulgaris Interactomes

There are 79 PPIs from the gold standard positive set where both proteins are found somewhere among the 1,242 proteins in the tagless fractions. 18 of these are in the 200 high confidence tagless PPIs, thus the false negative rate is 1 - (18/79) = 77%. The false negative rate for our *D. vulgaris* AP-MS interactome has previously been estimated at 69% by a similar approach (6).

Out of 459 AP-MS PPIs and 200 high confidence tagless PPIs identified in this study, 60 are present in both sets. Of the AP-MS PPIs, both partner proteins for 308 pairs are found somewhere in the tagless fractions, though not necessarily in the same fractions. Thus, the percent of AP-MS PPIs not found in the tagless fractions is 1 - (60/308) = 80%, which is quite close to the 77% false negative rate we estimate for the tagless method.

Of our 200 high confidence tagless PPIs, both partner proteins for 143 pairs are among the proteins detected in our previous AP-MS screen, though not necessarily in the same affinity purifications. Thus, the percent of high confidence tagless PPIs not found to interact in the AP-MS screen is 1 - (60/143) = 58%, which is actually lower than the 69% false negative rate for the AP-MS screen. The overlap between our AP-MS and tagless interactomes is thus close to that expected based on their false negative rates and could be said to provide partially independent support for these false negative rate estimates.

### Reanalysis of Havugimana et al.'s and Wan et al.'s Tagless Interactomes

Havugimana *et al.* and Wan *et al.* defined gold standard positive and negative protein pairs using the CORUM data set (37). Havugimana *et al.* divided these each into four independent quarters (16) and used two gold positive/negative quarters in a machine learning approach to define 35,956 “tagless-only” PPIs at an estimated 20% FDR based on copurification of nuclear and, separately, cytoplasmic fractions (supplemental Data set S6). The other two gold positive/negative quarters were used in a subsequent filtering and clustering step that employed gene expression and other data to identify 13,993 “high confidence” PPIs from the tagless-only PPIs, again at an estimated 20% FDR (16) (supplemental Data set S6). Of these PPIs, 4596 were identical to PPIs in the CORUM gold standards, whereas the remaining 9395 were novel (supplemental Table S4). Wan *et al.* define 16,655 high confidence PPIs using a filtered set of CORUM PPIs as gold standards (see their Supplemental Table 2 (19)). Of these, 4176 were identical to PPIs in the CORUM gold standards, whereas the remaining 12,479 were novel (supplemental Table S5).

For Havugimana *et al.*'s 35,956 tagless-only PPIs, we re-estimated the FDR using that portion of the complete gold standard CORUM data set held out during training of the classifiers (supplemental Data sets S7 and S8). Of the 9188 pairs in the cytoplasmic fraction, there are 296 positive and 773 negative held out CORUM gold standard PPIs, a 72% FDR. Of the 27,211 pairs in the nuclear fraction, there are 142 positive and 643 negative held out gold standard PPIs, an 82% FDR. Overall the FDR for the tagless-only protein pairs is thus 76%. Separately, if we calculate the FDR of the tagless only PPIs using the same portion of the gold standards that Havugimana *et al.* used, we replicate their FDR estimate of 20%. Assuming that the held in and held out parts of the gold standards were randomly selected from CORUM by Havugimana *et al.*, the two portions should give the same FDR estimate.

We also employed a total of 114,754 PPIs from three BioGrid data sets (38) that are each largely independent of the CORUM gold standards used by Havugimana *et al.* and Wan *et al.* (supplemental Data set S6). The three BioGrid data sets are derived from AP-MS data, Y2H data and other physical interaction assays respectively. The overlap between each BioGrid data set and Havugimana *et al.* 's and Wan *et al.* 's PPIs are shown in supplemental Tables S4 and S5 respectively. The overlap is on average seven- to eight-fold higher for PPIs that were also in the gold standard positive sets than for the novel PPIs identified in the high confidence sets. We assume that the PPIs from the CORUM gold standard positive sets should be equally well supported by BioGrid data as the novel high confidence PPIs. Thus, even if the CORUM gold standard positive sets used by Havugimana *et al.* and Wan *et al.* were 100% accurate, the novel PPI sets would be only 12.5% (100/8) to 14.3% (100/7) accurate. This implies that the FDRs for the novel protein pairs should be at least 85%, and will be higher if the CORUM gold standards contain a significant number of false positives.

A concern with our analysis is the possibility that the *bona fide* PPIs in the BioGrid data sets largely overlap with the CORUM data set, whereas the false positives in BioGrid may not. To test this we performed the following comparison. The overlap between the BioGrid AP-MS data set and the other two BioGrid data sets combined contains 5,566 PPIs, corresponding to a 14% or a 16% overlap depending on the direction considered. If the same overlap analysis is repeated, but those PPIs also present in CORUM are removed from each of the three BioGrid data sets, the overlap now contains 4201 PPIs, or 11% or 12% of PPIs. That is, the non CORUM part of the BioGrid AP-MS data set is similarly enriched for *bona fide* PPIs as the complete BioGrid AP-MS data set. Therefore, the novel PPIs identified by Havugimana *et al.* and Wan *et al.* at high confidence are not well supported by the data in BioGrid.

We have also determined the overlap between the novel PPIs identified in the two high confidence interactomes. Out of the 9395 and 12,479 novel PPIs in the two interactomes, only 652 PPIs are found in both. That is only 6.9% of Havugimana *et al.* 's novel PPIs are found in Wan *et al.* 's interactome and 5.2% of Wan *et al.* 's novel PPIs are found in Havugimana *et al.* 's interactome.

### Data Reporting

All raw MS files and associated ProteinPilot search engine results files were uploaded to the UCSD Center for Computational Mass Spectrometry, MassIVE, and can be downloaded on line (MassIVE identifier: MSV000079440; ProteomeXchange identifier: PXD003392). A spectral library containing annotated MS/MS spectra for DvH protein identifications, including MS/MS spectra for the great majority of proteins identified on the basis of a single peptide has been deposited at the Panoramaweb site (39) and can be viewed at Panorama Public, project title: “Tagless_analysis_of_protein-protein_interactions_in_Desulfovibrio_vulgaris”. A small minority of single peptide hit spectra that could not be uploaded to Panoramaweb are provided in supplemental Dataset S9. The protein interactions have been submitted to the IntAct database and assigned the identifier EBI-11695284.

## RESULTS

### A Large Scale Fractionation Detects Over a Third of the Proteome

**Fig. 1.**
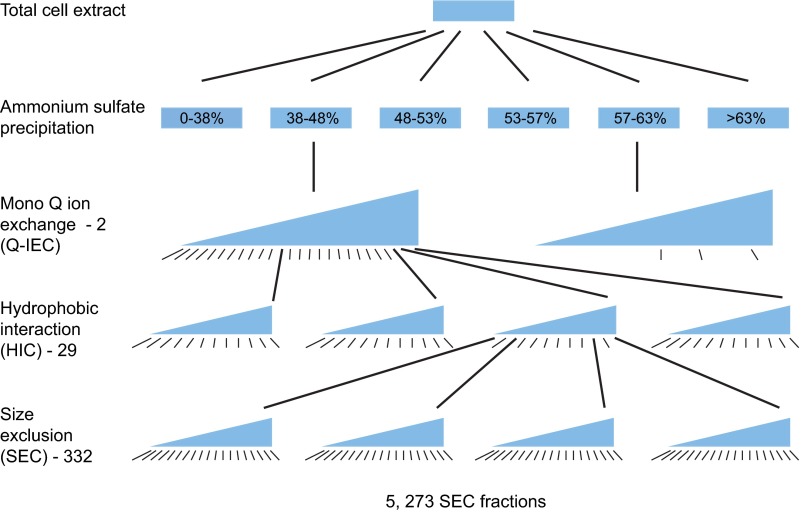
**Scheme for the tagless fractionation.** Ten grams of soluble protein cellular extract was subject to Ammonium Sulfate (AS) precipitation. Two out of the resulting six fractions were then subject to MonoQ ion exchange (Q-IEC) chromatography. 26 fractions from the Q-IEC column from the 38–48% AS step were separated by Hydrophobic interaction chromatography (HIC), whereas only 3 Q-IEC fractions from the 57–63% AS step were separated by HIC. 332 fractions from the HIC dimension were then each subject to Size exclusion chromatography (SEC), generating a set of 5273 SEC fractions that were subject to two dimensional iTRAQ mass spectrometry as described in Fig. 2*A*. Only a small subset of the HIC and SEC columns run are shown. The black lines below each fractionation step show those fractions subject to further separation or, in the case of the SEC fractions, to iTRAQ MS/MS analysis.

To allow detection of a large number of proteins after extensive fractionation, 10 g of soluble protein was extracted from a crude cell lysate of 400L of wild-type *D. vulgaris* cell culture. This crude extract was separated by ammonium sulfate precipitation, followed by three successive highly parallel chromatographic steps (Fig. 1): MonoQ anion exchange Chromatography (Q-IEC); Hydrophobic Interaction Chromatography (HIC); and Size Exclusion Chromatography (SEC) (Experimental Procedures, (13)). To avoid redundantly analyzing similar fractions, every second or third fraction from each proceeding separation step was used as input to the subsequent step (Experimental Procedures, supplemental Fig. S1).

**Fig. 2.**
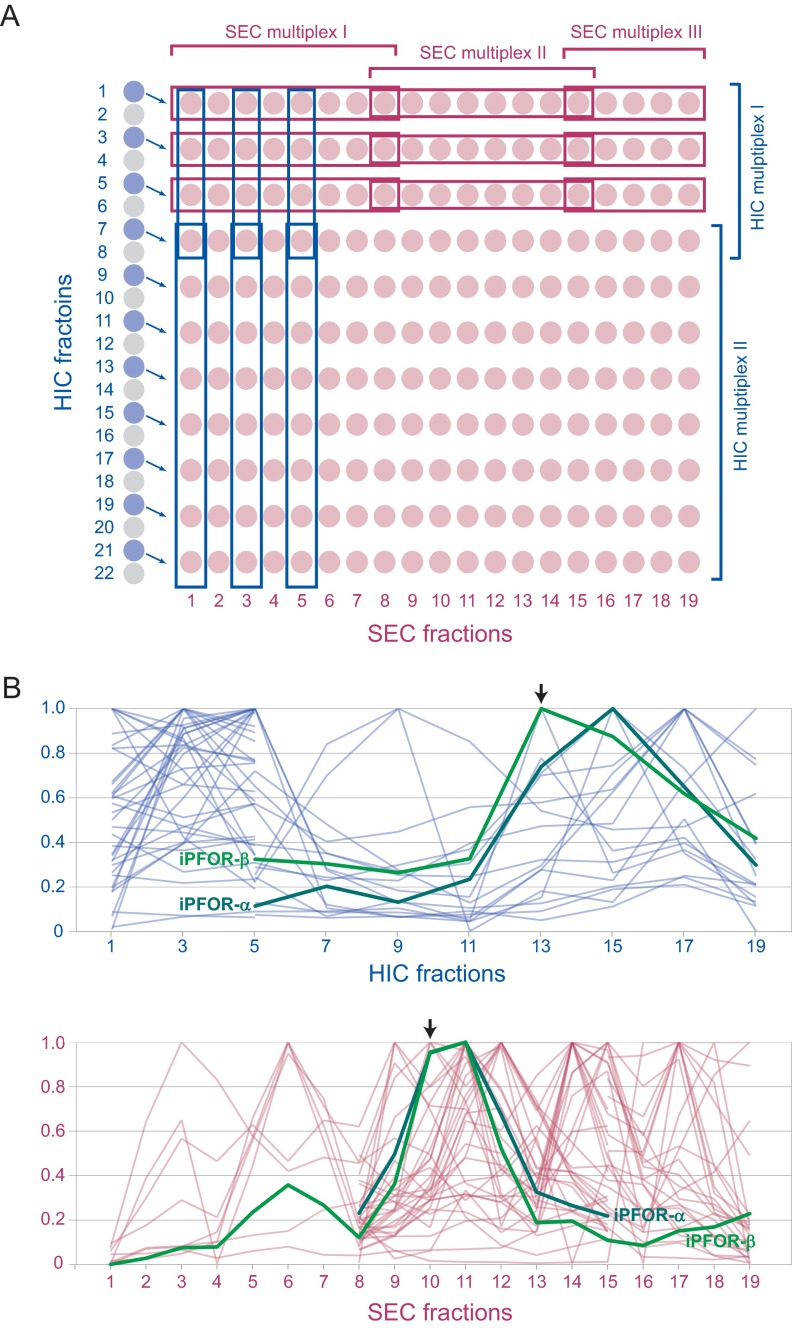
**Two dimensional iTRAQ labeling reveals elution profiles in SEC and HIC dimensions.** *A*, Left are shown 22 fractions eluted from a single HIC column. Every other fraction (11 blue disks) was separated on an SEC column, each producing 19 SEC fractions (red disks). The resulting total of 11 × 19 = 228 SEC fractions were digested with trypsin and each digested sample split into several portions to be used for mapping protein elution across the SEC and HIC dimensions (see Experimental Procedures). Two or more portions of each fraction were labeled with an iTRAQ reagent and combined with other fractions labeled with different isobaric iTRAQ reagents to form multiplexes. Multiplexes of up to 8 fractions are allowed by iTRAQ, and thus several multiplexes are required to determine the elution profiles across each column. A common “joint” fraction was included in adjacent multiplexes. Fractions were combined to form multiplexes that track protein elution along the SEC dimension (horizontal) and, separately, along the HIC dimension (vertical). For simplicity only three joined series of multiplexes are shown for each dimension, but from a single HIC column typically 10 joined series would cover the HIC dimension and 10–12 the SEC dimension. *B*, The iTRAQ elution profiles of proteins across the HIC dimension (top) and the SEC dimension (bottom) are shown. Only one joined series is shown for each dimension out of the larger number of series obtained for every HIC column run and its associated SEC fractions. The black arrows indicate the particular HIC fraction that was separated to produce the SEC profiles and the SEC fractions that were joined into multiplexes to generate profiles of a subset of the proteins eluting on the HIC dimension. The profiles for the alpha and beta subunits of indolepyruvate ferredoxin oxidoreductase (DVU1950 and DVU1951) are shown in bold green. The profiles of all other proteins detected are shown in red (SEC dimension) and blue (HIC dimension).

Each fraction from the SEC dimension was digested with trypsin and the resulting peptides labeled with isobaric tags for relative and absolute quantitation (iTRAQ) (40) to quantitate relative abundances of each protein between fractions (Experimental Procedures). Samples were combined to form iTRAQ multiplexes that contained between 3–8 SEC fractions for simultaneous mass spectrometry. Two patterns of iTRAQ labeling were used (Fig. 2*A*). In one, successive fractions from the same SEC column were labeled to determine the elution profiles of each protein across that column. In the second, the equivalent fractions from multiple SEC columns (*i.e.* fractions with the same retention time, same sized proteins) were labeled to allow the elution of proteins across the HIC column to be inferred (Fig. 2*A*). A total of 1472 distinct iTRAQ-labeled multiplexes were obtained and assayed by MALDI MS (Experimental Procedures).

The proteins detected were relatively evenly distributed across the SEC fractions, with a median of 25 proteins per fraction (supplemental Fig. S2). A small number of proteins appeared in at least 500 fractions, whereas 56% of all proteins appeared in fewer than 50 (supplemental Fig. S3). The detected proteins span all functional classifications, but are biased toward genes that are more highly expressed (supplemental Fig. S4). 1242 proteins were confidently identified not counting ribosomal proteins and chaperonins, which were excluded from our analysis of interacting protein pairs (Experimental Procedures, supplemental Data sets S1 and S2). This represents 36% of the 3403 proteins annotated in the *D. vulgaris* genome.

### Known PPIs Have Highly Correlated Elution Profiles in Multiple Dimensions

There are 770,661 possible pairwise combinations among the 1242 proteins in our data set. Because of the extensive fractionation employed, however, for only 146,792 (19%) of these pairs do both members co-occur in at least one SEC or HIC iTRAQ multiplex (supplemental Data set S3). We refer to a case where two proteins are found in some of the same factions as a co-occurring pair. Members of the same protein complex should not only co-occur, though, but should also have similar elution profiles. Indeed, as an example, the alpha and beta subunits of indolepyruvate ferredoxin oxidoreductase have similar elution profiles in both the HIC and SEC dimensions, whereas many other proteins in these same fractions have very different profiles (Fig. 2*B*). Simlarly, members of other well characterized complexes also copurify closely with each other (supplemental Fig. S5). Therefore, to better quantitate the degree to which proteins copurify, Pearson cross-correlation values (CC values) were computed for each iTRAQ multiplex for both the SEC and separately the HIC dimensions. Each co-occurring protein pair was assigned the maximum CC value for that pair for the SEC and, separately, for the HIC dimension. Co-occurring pairs with higher CC values are more likely to be *bona fide* interacting members of a protein complex than are co-occurring pairs with low CC values.

We have established three independent “benchmark” sets of well characterized PPIs (6) (Experimental Procedures). One was based on protein interactions from the *E. coli* EcoCyc data set, which is a manually curated set of interactions identified from low throughput experiments in the literature (41). The other two comprise the 2–3% of protein pairs from the published AP-MS or Y2H screens that have been reciprocally confirmed as both bait-prey and prey-bait pairs in the same experiment. In addition, we identified a large set of “negative” protein pairs that are unlikely to interact, based on the failure to observe such interactions in extensive analyses of *E. coli* protein complexes (6) (Experimental Procedures). For each of these four sets, “interologs” were defined where both members of the pair were mapped to homologs in *D. vulgaris* and are present among the 1242 proteins identified in the tagless fractions (Experimental Procedures; supplemental Table S1).

**Fig. 3.**
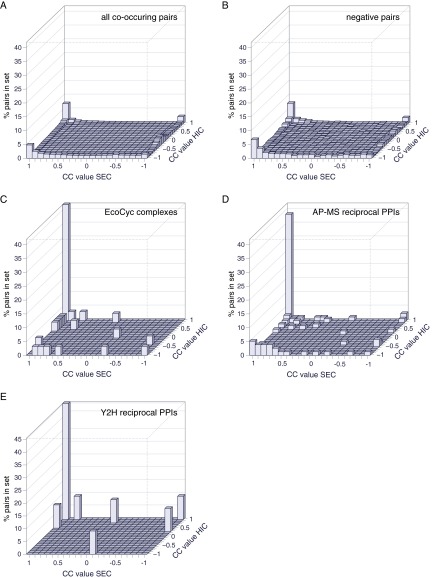
**Distribution of the Pearson cross correlation (CC) scores for the SEC and HIC dimensions.** Each plot shows the percentage of protein pairs in a given set that have the indicated maximum CC values for the SEC and the HIC dimensions. The two rows at −1 show the CC values where protein pairs are only detected in one dimension only. *A*, The set of all 146,792 co-occurring protein pairs. *B*, 1496 negative protein pairs unlikely to interact. *C*, 31 EcoCyc complex PPIs. *D*, 28 reciprocally confirmed AP-MS PPIs. *E*, 11 reciprocally confirmed Y2H PPIs. *D–E* are largely interologs of protein pairs defined using data from other species, except that some of the reciprocally confirmed AP-MS PPIs in *D* are from our *D. vulgaris* AP-MS interactome.

PPIs from our three benchmark sets co-occur in the same fractions 2.2–2.7 fold more often than do members of the negative protein pairs or of the 146,792 co-occurring pairs (supplemental Table S1). In addition, PPIs in the three benchmark sets are much more likely to have high maximum CC values in both the HIC and SEC dimensions than seen for all co-occurring protein pairs or for negative protein pairs (Fig. 3; supplemental Table S1). Thirty-six to 45% of co-occurring benchmark PPIs have CC values >0.85, whereas only 9–10% of all co-occurring pairs or negative pairs have CC values >0.85 (supplemental Table S1, column 3/column 6).

**Fig. 4.**
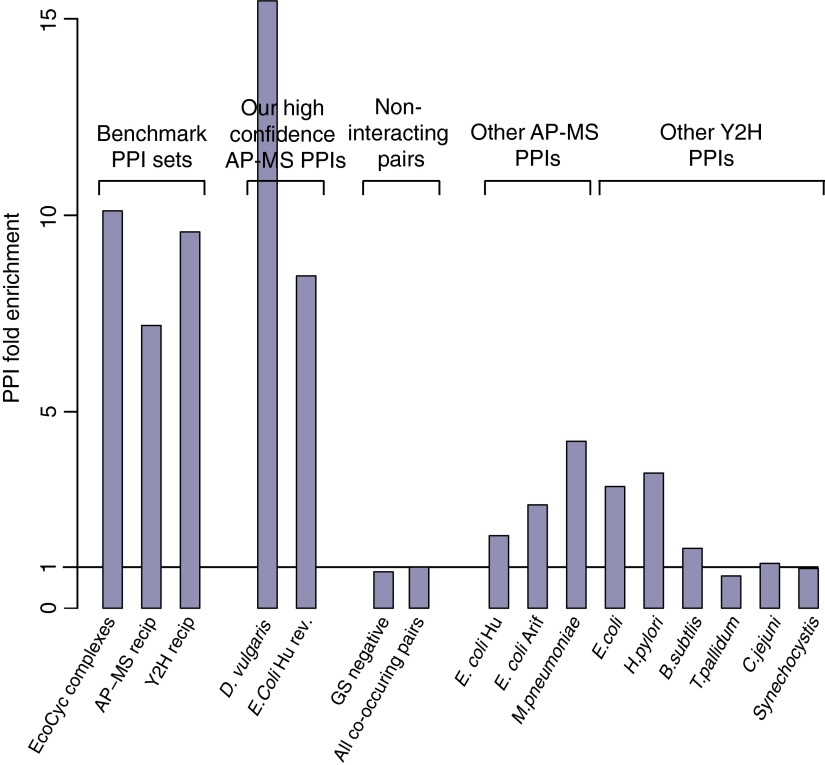
**Enrichment of highly correlated, co-occurring protein pairs.** The PPI fold enrichment of co-occurring protein pairs with CC values in both HIC and SEC dimensions ≥0.85 (Experimental Procedures; supplemental Table S1)). PPI fold enrichments are shown for different sets of protein pairs. To the left are the three benchmark data sets, though in this case *D. vulgaris* pairs were not included in the reciprocal AP-MS PPIs. Next are our two AP-MS interactomes for *D. vulgaris* and *E. coli*; the set of negative pairs unlikely to interact and the set of all co occurring protein pairs; and finally the nine earlier Y2H and AP-MS interactomes. The set of all co occurring protein pairs by definition have a PPI fold enrichment of 1.

To provide a measure that combines the propensity of *bona fide* PPIs to co-occur and have high CC values, we calculated a “PPI fold enrichment” value as follows. For each group of protein pairs, we calculated the fraction of its interologs that co-occur in both HIC and SEC dimensions with CC values ≥0.85 as a fraction of all interologs from that set present among the 1242 detected proteins (supplemental Table S1, column 7). We then determined the fold enrichment of these values over the values seen for all pairs of co-occurring interologs for the given species, irrespective of whether these interologs interact (Fig. 4; supplemental Table S1, column 11; see Methods for further details). The three benchmark sets have PPI fold enrichments of 7.2–10.1 *versus* values of 1.0 and 0.9 for all co-occurring pairs or the negative pairs.

### A Tagless Assay to Validate PPI Data Sets

Our previous work identified several hundred high confidence PPIs from AP-MS data for *D. vulgaris* and *E. coli*, and in addition suggested that nine other Y2H and AP-MS bacterial interactomes are dominated by protein pairs lacking the characteristics expected of *bona fide* PPIs (6). To further compare the properties of these various interactomes, we have exploited our tagless data set by calculating the PPI fold enrichment for each interactome. Our *D. vulgaris* and *E. coli* AP-MS interactomes are enriched in highly correlated protein pairs as well or better than the three benchmark PPIs. Importantly, our high confidence AP-MS data sets have PPI fold enrichments that are two to 20-fold higher than seen for the nine other proposed interactomes. Thus, consistent with our earlier results, the majority of protein pairs in these nine other interactomes are different in character from either our two high confidence AP-MS PPIs or the three benchmark PPIs.

Our *D. vulgaris* AP-MS interactome, though, has a PPI fold enrichment score that is ∼two-fold larger than that of our *E. coli* AP-MS interactome or the three benchmark data sets. This suggests that not all physical interactions are conserved between species, even when the proteins that participate in these interactions are conserved. This tendency is modest, however, and does not impact the comparison, for example, between the various PPI sets from *E. coli*. Thus although our tagless validation assay moderately favors sets of PPIs from the species used for the tagless fractionation, it can nevertheless clearly distinguish the properties of interactomes from a range of species.

### Identifying PPIs from Tagless Data

Given the strong tendency for well-characterized PPIs to have high CC values, it might be assumed that it is straight forward to identify *bona fide* PPIs from the tagless data. There are, however, 13,693 co-occurring protein pairs with maximum CC values ≥0.85 in both the HIC and SEC dimensions. The maximum CC values for all 146,792 co-occurring protein pairs show a similar distribution to those of the negative set of pairs (Fig. 3), suggesting that the majority of co-occurring pairs with CC values ≥0.85 do not in fact form stable interactions. Instead, most of these protein pairs likely represent the fortuitous comigration of proteins that result because of the large number proteins present in each fraction. Thus, additional criteria are needed to distinguish between protein pairs that physically interact from those that do not.

We therefore established logistic regression, machine learning to combine up to eight features and rank co-occurring pairs by the confidence that they are *bona fide* PPIs, see Experimental Procedures. Five features derive only from the tagless mass spectrometry data and include the CC values in the HIC and SEC dimensions as well as the frequency with which protein pairs co-occur in the same fractions. The remaining three features are based on genome location and capture the tendency for two genes to be present in the same operon across a range of species, using information from the Search Tool for the Retrieval of Interacting Genes/Proteins (STRING) (35). The logistic regression was trained on a gold standard positive set of likely PPIs and a gold standard negative set of noninteracting protein pairs (6) (Experimental Procedures). All eight features show strong enrichment of pairs from the gold positive set over pairs from the gold negative set (supplemental Figs. S6–S13), indicating that each feature can partially distinguish true positives from false positive PPIs. Cross-validation ensured that gold standard complexes used for training were excluded from the validation step.

When using just the five mass spectrometry only features in the logistic regression, 201 “MS-only” PPIs were identified at 20% FDR (supplemental Table S2). When using all eight features, 300 “MS+STRING” PPIs were detected at 20% FDR (supplemental Table S2). FDR estimates, however, are subject to error. For example, only two gold negative and nine gold positives protein pairs were detected among the MS-only protein pairs (supplemental Table S2). Such small numbers allow only an approximate FDR estimate to be made. Therefore, we adopted five additional PPI quality metrics to identify high confidence PPIs: the percent of protein pairs encoded in the same operon; the enrichment of protein pairs whose members share the same functional annotation by The Institute of Genome Research (TIGR role); the fraction of protein pairs found in our high confidence AP-MS interactome for *D. vulgaris*; the fraction of protein pairs found in at least one of the three AP-MS interactomes for *E. coli* or *M. pneumoniae*; and the fraction of PPIs that are found in at least one Y2H interactome (Experimental Procedures; (6)).

**Fig. 5.**
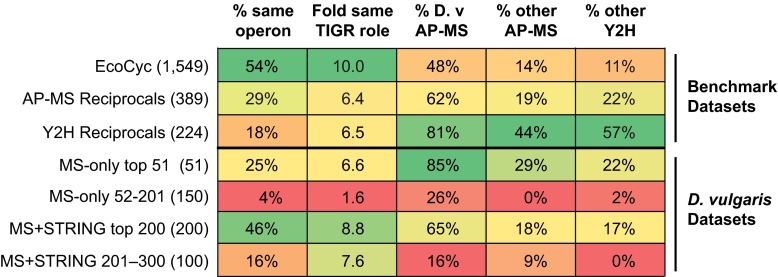
**PPI quality metrics for benchmark data sets and high and low confidence *D. vulgaris* tagless protein pair sets.** The top three rows show metrics for benchmark bacterial data sets: the EcoCyc complexes (41), and protein pairs that have been reciprocally confirmed in either four AP-MS studies, including ours, or in six Y2H studies (Experimental Procedures) (6). The remaining rows show metrics for sets of protein pairs identified by the MS-only and MS+STRING logistic regressions. The regression scores were used to rank and separate PPIs into a high and low scoring set in each case. The numbers of protein pairs in each set are given in brackets. The columns show from left to right: the percent of pairs whose members are encoded in the same operon; fold enrichment of pairs for which both members have the same TIGR role over that expected among randomly chosen pairs; percent overlap with PPIs from the *D. vulgaris* AP-MS interactome; percent overlap with a combined set of interologs from the three bacterial AP-MS interactomes for other bacterial species; and percent overlap with a combined set of interologs from the six bacterial Y2H interactomes (Experimental Procedures; supplemental Table S2).

The protein pairs in the MS-only and MS+STRING sets that have high logistic regression scores also have higher PPI quality metrics and more frequently include gold positive PPIs than the protein pairs with lower logistic regression scores (supplemental Data sets S3 and S4; Fig. 5). We therefore divided the logistic regression rank lists to select PPIs that had quality metrics similar to those of our three benchmark sets, which identified the top 51 MS-only PPIs and the top 200 MS+STRING PPIs. Those MS-only and MS+STRING protein pairs excluded from these “top” sets have much lower PPI quality metric scores and include no gold positive PPIs, suggesting that they are predominantly false positives (supplemental Table S2, Fig. 5).

We then compared the overlap between the protein pairs identified in the two top sets. The 18 PPIs from the top 51 MS-only set that are also found in the top 200 MS+STRING set contain virtually all of the PPIs validated by our quality metrics, whereas the nonoverlapping 33 pairs do not and thus are likely false positives (supplemental Table S2). We conclude that it is not possible to identify a useful number of PPIs from our tagless data set at an acceptable FDR without using additional information, such as genome location. We therefore designated the top 200 MS+STRING set as our “high confidence” tagless set, supplemental Data set S4.

Although these high confidence PPIs were defined in part using genome location data, they are well supported by the tagless fractionation data. The 200 PPIs each have CC values ≥0.85 in at least one HIC or SEC column (supplemental Data set S4). In addition, collectively they are enriched in each of the five MS only regression features to a similar degree as the gold standard positives (compare supplemental Fig. S14 with supplemental Figs. S6–S13). Thus although the genome location data was essential for identifying bona fide PPIs, our logistic regression did not rely solely on this information, but instead weighted strongly the evidence for physical interaction provided by the tagless data.

### Validating the Tagless Interactome

To provide separate evidence validating our high confidence tagless PPIs, we compared them to the PPIs we identified for the same species by AP-MS. Out of a total of 599 PPIs present in at least one of these two interactomes, 60 were identified in both, 140 only in the tagless interactomes and 399 only in the AP-MS interactome. Although this overlap may seem small, if only cases where both members of a pair are present in each interactome are considered, 65% of tagless PPIs are identified in the AP-MS interactome and 60% of the AP-MS PPIs are identified in the tagless interactome. In addition, because both assays fail to detect many PPIs, a complete overlap is not expected. Based on the false negative rates of 69% for the AP-MS study and 77% for the tagless screen, the overlap between the AP-MS and tagless interactome is within 3% of what one would expect, see Experimental Procedures. Thus, our two *D. vulgaris* screens strongly cross validate each other.

**Fig. 6.**
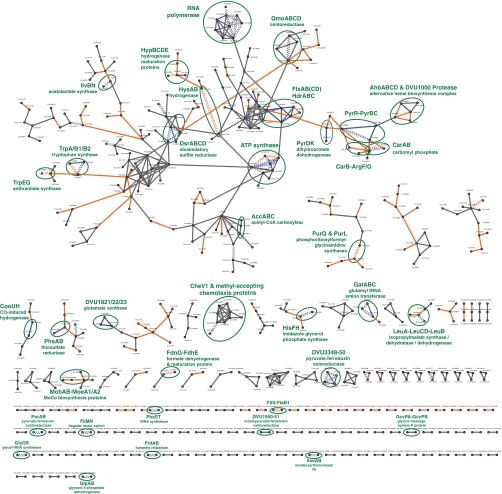
**Combined AP-MS and tagless interactome for *D. vulgaris*.** All 599 interactions present in the union of our high confidence AP-MS and tagless interactomes are shown. PPIs in both the AP-MS and tagless interactomes are shown in blue; PPIs only present in the tagless interactome are shown in orange; and PPIs only in the AP-MS interactome are shown in gray. PPIs also supported by additional evidence from gold standard positives or from AP-MS or Y2H screens in other bacteria are shown by wavy lines. Green ellipses show examples of complexes annotated in other species, as labeled.

**Fig. 7.**
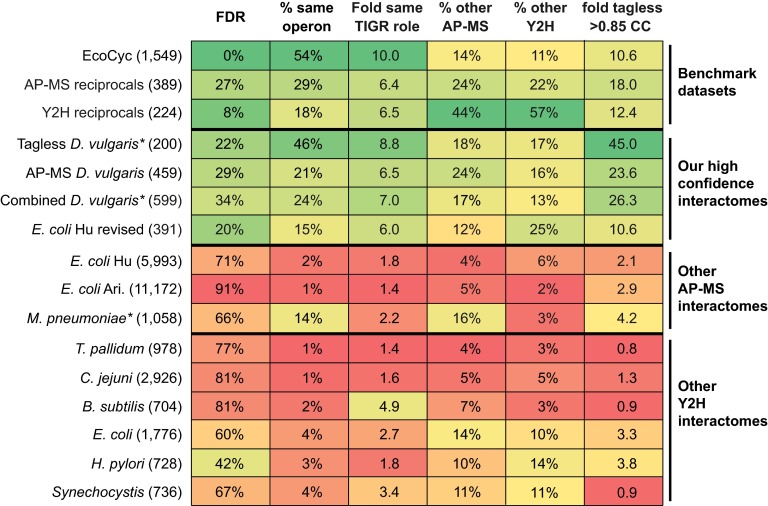
**PPI quality metrics for benchmark data sets and proposed bacterial interactomes.** The top three rows show metrics for the three benchmark data sets described in Fig. 5. The remaining rows show metrics for our tagless, AP-MS and combined interactomes; the three other AP-MS interactomes (25–27); and the six Y2H data sets (28–33), see Experimental Procedures. The numbers of protein pairs in each set are given in brackets. The left most column shows the FDR estimated using gold standard positive and negatives sets based only on complexes from the EcoCyc data set or, in the case of the non *E. coli* studies, their interologs. The right most column shows the fold enrichment of highly correlated co-occurring protein pairs found in our tagless assay (supplemental Table S1). The remaining columns are as in Fig. 5. Data sets for which genome location data was used in addition to interaction data to identify protein pairs are indicated with *.

To further validate the accuracy of high confidence tagless PPIs for *D. vulgaris*, we first combined it with the AP-MS PPIs to create a single interactome (Fig. 6; supplemental Data set S5). We then compared the enrichment of multiple PPI quality metrics in this combined interactome, our tagless interactome, our AP-MS interactome, the three benchmark sets, and the nine other bacterial interactomes (supplemental Table S3; Fig. 7). For all six quality metrics examined, our combined and our tagless interactomes have similar properties to the three benchmark sets and our AP-MS interactomes, whereas the other nine interactomes have lower quality metric scores. Three of these metrics, though, were partially or fully used in the selection of the tagless interactome: same operon, same TIGR role, and fold enrichment in high CC values. This selection bias could lead to false positives in the tagless interactome being enriched in these three metrics. These biases, however, do not apply to the other three PPI quality metrics used in Fig. 7. In addition, the tagless and the combined interactomes have a connectivity similar to that of the EcoCyc data set and of our AP-MS interactomes and are much less connected than the nine other interactomes (supplemental Table S3). Thus, the properties of the tagless and the combined interactomes further support the conclusion that the nine other bacterial interactomes are dominated by protein pairs that are not well supported by other independent data. The similarities in properties of our tagless and the combined interactomes and the gold standards provides additional evidence that our tagless PPIs are strongly enriched in *bona fide* PPIs.

The accuracy of our tagless interactome is further supported by other experimental evidence on individual protein complexes (see supplemental Text S1 for detailed discussion). For example, the 60 PPIs identified by both AP-MS and tagless methods include many protein complexes well characterized in other experiments: *e.g.* ATP synthase, dissimilatory sulfite reductase, carbomyl phosphate synthase, and RNA polymerase (Fig. 6, supplemental Fig. S15). In addition, many PPIs identified at high confidence by the tagless method but not by our AP-MS screen are supported by physical interaction data from another species: *e.g.* the interaction between flagella proteins FliS and FlaB1; formate dehydrogenase and a formate dehydrogenase formation protein; HypD and HypE hydrogenase maturation proteins; phosphoribosylformylglycinamidine synthases I and II; and Qmo oxidoreductase and adenyl sulfate reductase alpha subunit (Fig. 6, supplemental Fig. S15).

### Reanalysis of Two Human Tagless Interactomes

Of the other tagless screens published to date, only two closely related surveys by Havugimana *et al.* and Wan *et al.* are comparable to ours in that they measure quantitative copurification profiles across multiple separation methods on a large scale (16, 19). From 5584 proteins detected in human cell lines, Havugimana *et al.* defined 35,956 “tagless-only” PPIs at 20% FDR based only on the tagless copurification of protein pairs. They then identified 13,993 “high confidence” PPIs by filtering the tagless-only PPIs using data on mRNA coexpression and protein interactions in other species. Wan *et al.* extended this study by including further tagless data for humans and for other animals to generate 16,655 high confidence human PPIs. Because the two high confidence human tagless interactomes identify 20 times more PPIs per detected protein than our tagless interactome, we have reanalyzed Havugimana *et al.* and Wan *et al.* 's results to determine if their approach is more effective than ours.

Havugimana *et al.* determined the FDR of the 35,956 tagless-only PPIs using one half of a set of gold standard positive and negative PPIs that were based on the CORUM database. We re-estimated the FDR for the tagless-only PPIs using the other half of Havugimana *et al.* 's gold standards that had been held out (see Experimental Procedures). This re-estimated FDR is 76%, in contrast to the estimate of 20% that both Havugimana *et al.* and we obtain using the original half of the gold standards. This disparity suggest that the classifier generated by Havugimana *et al.* was over fit for characteristics specific to the originally used half of the gold standards and as a result their FDR estimate is unreliable.

To estimate the accuracy of the two high confidence interactomes, we first employed 114,754 PPIs from three BioGrid data sets (38) that are largely independent from the CORUM gold standards used by Havugimana *et al.* and Wan *et al.*, see Experimental Procedures. We divided each of the high confidence tagless interactomes into ∼ 4000 PPIs that were part of the gold standard positives used to define them and >9000 novel PPIs (supplemental Tables S4 and S5). The novel PPIs overlap with the BioGrid data seven- to eight-fold less often than the PPIs that were gold standard positives (supplemental Tables S4 and S5,). This implies that the FDRs for the novel protein pairs should be at least 85%, and will be higher if the gold standards contain a significant number of false positives (see Experimental Procedures for explanation).

In addition, despite the fact that the two interactomes are based on similar data and analysis methods, the overlap between the novel PPIs identified in both is very low. Out of the 9395 and 12,479 novel PPIs in the two tagless interactomes, only 652 PPIs are in common, less that a 7% overlap (Experimental Procedures). This poor reproducibility is consistent with our 85% FDR estimate for the novel protein pairs.

## DISCUSSION

It is challenging to confidently identify PPIs using high throughput methods such as Y2H and AP-MS. For example, there is a poor overlap between protein pairs identified in different screens, even when these screens are performed in the same species (3, 4, 6, 42)). In addition, there are many low affinity, nonfunctional interactions that are not under evolutionary constraint but instead arise by chance between short segments of proteins (43). This has led to different interpretations of high throughput interactomes. One interpretation is that a high proportion of their protein pairs are false positives that do not interact (*e.g.* (3, 4)). Alternatively, it could be that most pairs are genuine interactions, but these are not well replicated between screens because of high false negative rates, the rapid evolution of PPIs between species, and/or the different specificities of the AP-MS and Y2H methods for detecting classes of interaction (*e.g.* (32, 44)).

To address these challenges, we have previously established a more stringent analysis strategy for AP-MS data and used it to identify several hundred PPIs each for the bacteria *D. vulgaris* and *E. coli* (6). These two interactomes have significantly lower FDRs than nine earlier AP-MS and Y2H interactomes for bacteria and are much more enriched in protein pairs that have similar functions or are reproducibly detected in other interaction assays (6) (Fig. 7). These results suggest that the nine earlier bacterial interactomes are dominated by false positives that do not interact.

Here we have provided further support for this idea using a tagless assay. In this approach, a crude extract of native *D. vulgaris* proteins is subjected to extensive chromatographic separation and the relative levels of proteins eluting in each column fraction are quantitated using iTRAQ LC MALDI MS (Figs. 1 and 2). The protein partners from our two AP-MS interactomes copurify with each other as frequently as the protein pairs in three benchmark data sets of well characterized PPIs: the EcoCyc complexes from *E. coli* and the ∼3% of PPIs that are reciprocally confirmed as bait-prey and prey-bait pairs in nine Y2H or AP-MS screens of bacteria (Fig. 4). In contrast, the full set of all protein pairs from the nine other bacterial interactomes copurify two- to 20-fold less often (Fig. 4). Because none of the other nine bacterial interactomes or the benchmark data sets are from the same class of bacteria as *D. vulgaris*, the comparison of these data sets is limited to that subset of proposed PPIs whose partners are both present in *D. vulgaris*. Nonetheless, the results in our tagless validation assay mirrors other properties measured for all members of each interactome, such as the tendency to be encoded in the same operon or to share the same functional annotation (Fig. 7), indicating that our cross species comparison is valid.

It might be suggested that most protein pairs detected by Y2H are *bona fide* PPIs, but because they interact at low affinity they cannot be detected by the tagless method. Indeed, Y2H can detect interactions of as little as micro molar affinity (45), and these will not survive the hours of fractionation and varied buffer conditions employed in our tagless protocol. The benchmark Y2H reciprocal PPIs, however, are as well enriched in protein pairs that copurify in the tagless assay as our *E. coli* AP-MS PPIs and the other two benchmark data sets (Fig. 4). This suggests that interologs for most Y2H protein pairs fail to copurify in the tagless assay, not because they are low affinity, *bona fide* PPIs, but because they are inherently irreproducible even in the Y2H assay and thus likely false positives.

In addition to using our tagless assay to validate existing interactomes, we have also used it in combination with genomic location data to identify *de novo* 200 PPIs. These tagless-identified PPIs have similar properties to our AP-MS interactomes and the three benchmark interactomes (Fig. 7; supplemental Table S3). In addition, these 200 PPIs overlap with our AP-MS interactome for *D.vulgaris* within 3% of expectation, once FDRs and false negative rates are taken into account, Experimental Procedures. Thus, two different biochemical purification screens, AP-MS and tagless, both identify sets of protein pairs with similar characteristics.

Although we have not analyzed the published eukaryotic AP-MS and Y2H interactomes, the protocols used to identify these PPIs are similar to those used to identify the nine bacterial interactomes we examined (42, 46–52). As a result, the, eukaryotic interactomes could also have higher FDRs than originally claimed (6).

Several other variants of the tagless method have been published (14–19). The two most similar to ours also generate quantitative, coelution profiles across several chromatography steps (16, 19). In these studies of human cell lines, ∼20-fold more PPIs were reported per detected protein than identified in our *D. vulgaris* study. Although this difference might reflect a difference in the connectivity of interactomes in animals *versus* bacteria, our reanalysis of the human tagless data sets suggest that at least 85% of the novel protein pairs identified are false positives, see “Results.” Just as Y2H and AP-MS data sets can suffer from a high background of false positives, tagless data sets also require careful analysis to limit the FDR.

The only other system-wide tagless screen was performed on the archaeon *Pyrococcus furiosus* (14). This screen required that PPIs be encoded by genes located close to each other in the genome, similar to the strategy we found necessary to identify high confidence PPIs from our tagless data set. The *P. furiosus* screen, however, did not create quantitative elution profiles of protein abundance, which our analysis indicates is a powerful indicator of the likelihood that a pair of proteins physically interact (Figs. 2*B*, 3 and 4; supplemental Figs. S5–S7).

Tagless screens provide a useful new class of evidence for deciphering the structure of protein interactomes. Given the unmet challenge of determining a full interactome at both low FDR and low false negative rate, however, additional refinements of this and the other high throughput screens will be required to gain a complete picture of protein interaction networks.

## Supplementary Material

Supplemental Data

## Footnotes

^1^ The abbreviations used are:

PPI: Protein – protein interaction
AP-MS: affinity purification – mass-spectrometry
CC: Pearson cross correlation
FDR: false discovery rate
HIC: hydrophobic interaction chromatography
iTRAQ: isobaric tags for relative and absolute quantitation
Q-IEC: MonoQ anion exchange chromatography
SEC: size exclusion chromatography
STRING: Search Tool for the Retrieval of Interacting Genes/Proteins
TIGR: The Institute of Genome Research
Y2H: yeast two hybrid.

## References

[1] Alberts B., Johnson A., Lewis J., Raff M., Roberts K., and Walter P. (2007) Molecular Biol. Cell, 5 edition ed., Garland Science, New York.

[2] Kristensen A. R., and Foster L. J. (2013) High throughput strategies for probing the different organizational levels of protein interaction networks. Mol. bioSystems 9, 2201–2212 10.1039/c3mb70135b 23861068

[3] von Mering C., Krause R., Snel B., Cornell M., Oliver S. G., Fields S., and Bork P. (2002) Comparative assessment of large-scale data sets of protein-protein interactions. Nature 417, 399–403 12000970 10.1038/nature750

[4] Edwards A. M., Kus B., Jansen R., Greenbaum D., Greenblatt J., and Gerstein M. (2002) Bridging structural biology and genomics: assessing protein interaction data with known complexes. Trends Gen. 18, 529–536 10.1016/s0168-9525(02)02763-4 12350343

[5] Vidal M., Cusick M. E., and Barabasi A. L. (2011) Interactome networks and human disease. Cell 144, 986–998 21414488 10.1016/j.cell.2011.02.016 PMC3102045

[6] Shatsky M., Allen S., Gold B. L., Liu N. L., Juba T. R., Reveco S. A., Elias D. A., Prathapam R., He J., Yang W., Szakal E. D., Liu H., Singer M. E., Geller J. T., Lam B. R., Saini A., Trotter V. V., Hall S. C., Fisher S. J., Brenner S. E., Chhabra S. R., Hazen T. C., Wall J. D., Witkowska H. E., Biggin M. D., Chandonia J.-M., and Butland G. (2016) Bacterial interactomes: interacting protein partners share similar function and are validated in independent assays more frequently than previously reported. 15, 1539–1555 10.1074/mcp.M115.054692 PMC4858938 26873250

[7] McHenry C. S., and Crow W. (1979) DNA polymerase III of Escherichia coli. Purification and identification of subunits. J. Biol. Chem. 254, 1748–1753 368075

[8] Link A. J., Fleischer T. C., Weaver C. M., Gerbasi V. R., and Jennings J. L. (2005) Purifying protein complexes for mass spectrometry: applications to protein translation. Methods 35, 274–290 15722224 10.1016/j.ymeth.2004.08.019

[9] Camacho-Carvajal M. M., Wollscheid B., Aebersold R., Steimle V., and Schamel W. W. A. (2004) Two-dimensional Blue native/SDS gel electrophoresis of multi-protein complexes from whole cellular lysates: a proteomics approach. Mol. Cell. Proteomics 3, 176–182 14665681 10.1074/mcp.T300010-MCP200

[10] Austin R. J., and Biggin M. D. (1996) Purification of the Drosophila RNA polymerase II general transcription factors. Proc. Natl. Acad. Sci. U.S.A. 93, 5788–5792 8650170 10.1073/pnas.93.12.5788 PMC39139

[11] Dong M., Yang L. L., Williams K., Fisher S. J., Hall S. C., Biggin M. D., Jin J., and Witkowska H. E. (2008) A “tagless” strategy for identification of stable protein complexes genome-wide by multidimensional orthogonal chromatographic separation and iTRAQ reagent tracking. J. Proteome Res. 7, 1836–1849 18336004 10.1021/pr700624e

[12] Walian P. J., Allen S., Shatsky M., Zeng L., Szakal E. D., Liu H., Hall S. C., Fisher S. J., Lam B. R., Singer M. E., Geller J. T., Brenner S. E., Chandonia J. M., Hazen T. C., Witkowska H. E., Biggin M. D., and Jap B. K. (2012) High-throughput isolation and characterization of untagged membrane protein complexes: outer membrane complexes of Desulfovibrio vulgaris. J. Proteome Res. 11, 5720–5735 23098413 10.1021/pr300548d PMC3516867

[13] Han B. G., Dong M., Liu H., Camp L., Geller J., Singer M., Hazen T. C., Choi M., Witkowska H. E., Ball D. A., Typke D., Downing K. H., Shatsky M., Brenner S. E., Chandonia J. M., Biggin M. D., and Glaeser R. M. (2009) Survey of large protein complexes in D. vulgaris reveals great structural diversity. Proc. Natl. Acad. Sci. U.S.A. 106, 16580–16585 19805340 10.1073/pnas.0813068106 PMC2742403

[14] Menon A. L., Poole F. L., Cvetkovic A., Trauger S. A., Kalisiak E., Scott J. W., Shanmukh S., Praissman J., Jenney F. E., Wikoff W. R., Apon J. V., Siuzdak G., and Adams M. W. W. (2009) Novel multiprotein complexes identified in the hyperthermophilic archaeon Pyrococcus furiosus by non-denaturing fractionation of the native proteome. Mol. Cell. Proteomics 8, 735–751 19043064 10.1074/mcp.M800246-MCP200 PMC2668238

[15] Gordon S. M., Deng J., Tomann A. B., Shah A. S., Lu L. J., and Davidson W. S. (2013) Multi-dimensional co-separation analysis reveals protein-protein interactions defining plasma lipoprotein subspecies. Mol. Cell. Proteomics 12, 3123–3134 23882025 10.1074/mcp.M113.028134 PMC3820928

[16] Havugimana P. C., Hart G. T., Nepusz T., Yang H., Turinsky A. L., Li Z., Wang P. I., Boutz D. R., Fong V., Phanse S., Babu M., Craig S. A., Hu P., Wan C., Vlasblom J., Dar V. U., Bezginov A., Clark G. W., Wu G. C., Wodak S. J., Tillier E. R., Paccanaro A., Marcotte E. M., and Emili A. (2012) A census of human soluble protein complexes. Cell 150, 1068–1081 22939629 10.1016/j.cell.2012.08.011 PMC3477804

[17] Heide H., Bleier L., Steger M., Ackermann J., Drose S., Schwamb B., Zornig M., Reichert A. S., Koch I., Wittig I., and Brandt U. (2012) Complexome profiling identifies TMEM126B as a component of the mitochondrial complex I assembly complex. Cell Metab. 16, 538–549 22982022 10.1016/j.cmet.2012.08.009

[18] Kristensen A. R., Gsponer J., and Foster L. J. (2012) A high-throughput approach for measuring temporal changes in the interactome. Nat. Methods 9, 907–909 22863883 10.1038/nmeth.2131 PMC3954081

[19] Wan C., Borgeson B., Phanse S., Tu F., Drew K., Clark G., Xiong X., Kagan O., Kwan J., Bezginov A., Chessman K., Pal S., Cromar G., Papoulas O., Ni Z., Boutz D. R., Stoilova S., Havugimana P. C., Guo X., Malty R. H., Sarov M., Greenblatt J., Babu M., Derry W. B., Tillier E. R., Wallingford J. B., Parkinson J., Marcotte E. M., and Emili A. (2015) Panorama of ancient metazoan macromolecular complexes. Nature 525, 339–344 26344197 10.1038/nature14877 PMC5036527

[20] Garczarek F., Dong M., Typke D., Witkowska H. E., Hazen T. C., Nogales E., Biggin M. D., and Glaeser R. M. (2007) Octomeric pyruvate-ferredoxin oxidoreductase from Desulfovibrio vulgaris. J. Struct. Biol. 159, 9–18 17400475 10.1016/j.jsb.2007.01.020

[21] Papac D. I., Briggs J. B., Chin E. T., and Jones A. J. (1998) A high-throughput microscale method to release N-linked oligosaccharides from glycoproteins for matrix-assisted laser desorption/ionization time-of-flight mass spectrometric analysis. Glycobiology 8, 445–454 9597542 10.1093/glycob/8.5.445

[22] Basa L. J., Katta V., Haskins W. E., and Cochran P. K. (2005) Proceedings of the 53rd ASMS Conference on Mass Spectrometry and Allied Topics, San Antonio, TX.

[23] Liu H., Yang L., Khainovski N., Dong M., Hall S. C., Fisher S. J., Biggin M. D., Jin J., and Witkowska H. E. (2011) Automated iterative MS/MS acquisition: a tool for improving efficiency of protein identification using a LC-MALDI MS workflow. Anal. Chem. 83, 6286–6293 21761829 10.1021/ac200911v

[24] Shilov I. V., Seymour S. L., Patel A. A., Loboda A., Tang W. H., Keating S. P., Hunter C. L., Nuwaysir L. M., and Schaeffer D. A. (2007) The Paragon Algorithm, a next generation search engine that uses sequence temperature values and feature probabilities to identify peptides from tandem mass spectra. Mol. Cell. Proteomics 6, 1638–1655 17533153 10.1074/mcp.T600050-MCP200

[25] Hu P., Janga S. C., Babu M., Díaz-Mejía J. J., Butland G., Yang W., Pogoutse O., Guo X., Phanse S., Wong P., Chandran S., Christopoulos C., Nazarians-Armavil A., Nasseri N. K., Musso G., Ali M., Nazemof N., Eroukova V., Golshani A., Paccanaro A., Greenblatt J. F., Moreno-Hagelsieb G., and Emili A. (2009) Global functional atlas of Escherichia coli encompassing previously uncharacterized proteins. PLoS Biol. 7, e96 19402753 10.1371/journal.pbio.1000096 PMC2672614

[26] Arifuzzaman M., Maeda M., Itoh A., Nishikata K., Takita C., Saito R., Ara T., Nakahigashi K., Huang H. C., Hirai A., Tsuzuki K., Nakamura S., Altaf-Ul-Amin M., Oshima T., Baba T., Yamamoto N., Kawamura T., Ioka-Nakamichi T., Kitagawa M., Tomita M., Kanaya S., Wada C., and Mori H. (2006) Large-scale identification of protein-protein interaction of Escherichia coli K-12. Genome Res. 16, 686–691 16606699 10.1101/gr.4527806 PMC1457052

[27] Kühner S., van Noort V., Betts M. J., Leo-Macias A., Batisse C., Rode M., Yamada T., Maier T., Bader S., Beltran-Alvarez P., Castaño-Diez D., Chen W. H., Devos D., Güell M., Norambuena T., Racke I., Rybin V., Schmidt A., Yus E., Aebersold R., Herrmann R., Böttcher B., Frangakis A. S., Russell R. B., Serrano L., Bork P., and Gavin A. C. (2009) Proteome organization in a genome-reduced bacterium. Science 326, 1235–1240 19965468 10.1126/science.1176343

[28] Titz B., Rajagopala S. V., Goll J., Hauser R., McKevitt M. T., Palzkill T., and Uetz P. (2008) The binary protein interactome of Treponema pallidum–the syphilis spirochete. PLoS ONE 3, e2292 18509523 10.1371/journal.pone.0002292 PMC2386257

[29] Parrish J. R., Yu J., Liu G., Hines J. A., Chan J. E., Mangiola B. A., Zhang H., Pacifico S., Fotouhi F., DiRita V. J., Ideker T., Andrews P., and Finley R. L. Jr. (2007) A proteome-wide protein interaction map for Campylobacter jejuni. Genome Biol. 8, R130 17615063 10.1186/gb-2007-8-7-r130 PMC2323224

[30] Marchadier E., Carballido-Lopez R., Brinster S., Fabret C., Mervelet P., Bessieres P., Noirot-Gros M. F., Fromion V., and Noirot P. (2011) An expanded protein-protein interaction network in Bacillus subtilis reveals a group of hubs: Exploration by an integrative approach. Proteomics 11, 2981–2991 21630458 10.1002/pmic.201000791

[31] Rajagopala S. V., Sikorski P., Kumar A., Mosca R., Vlasblom J., Arnold R., Franca-Koh J., Pakala S. B., Phanse S., Ceol A., Hauser R., Siszler G., Wuchty S., Emili A., Babu M., Aloy P., Pieper R., and Uetz P. (2014) The binary protein-protein interaction landscape of Escherichia coli. Nat. Biotechnol. 32, 285–290 24561554 10.1038/nbt.2831 PMC4123855

[32] Hauser R., Ceol A., Rajagopala S. V., Mosca R., Siszler G., Wermke N., Sikorski P., Schwarz F., Schick M., Wuchty S., Aloy P., and Uetz P. (2014) A Second-generation Protein-Protein Interaction Network of Helicobacter pylori. Mol. Cell. Proteomics. 13, 1318–1329 24627523 10.1074/mcp.O113.033571 PMC4014287

[33] Sato S., Shimoda Y., Muraki A., Kohara M., Nakamura Y., and Tabata S. (2007) A large-scale protein protein interaction analysis in Synechocystis sp. PCC6803. DNA Res 14, 207–216 18000013 10.1093/dnares/dsm021 PMC2779905

[34] Wodak S. J., Vlasblom J., Turinsky A. L., and Pu S. (2013) Protein-protein interaction networks: the puzzling riches. Current Opinion Structural Biol. 23, 941–953 10.1016/j.sbi.2013.08.002 24007795

[35] Franceschini A., Szklarczyk D., Frankild S., Kuhn M., Simonovic M., Roth A., Lin J., Minguez P., Bork P., von Mering C., and Jensen L. J. (2013) STRING v9.1: protein-protein interaction networks, with increased coverage and integration. Nucleic Acids Res. 41, D808–815 23203871 10.1093/nar/gks1094 PMC3531103

[36] Park Y., and Marcotte E. M. (2012) Flaws in evaluation schemes for pair-input computational predictions. Nat. Methods 9, 1134–1136 23223166 10.1038/nmeth.2259 PMC3531800

[37] Ruepp A., Waegele B., Lechner M., Brauner B., Dunger-Kaltenbach I., Fobo G., Frishman G., Montrone C., and Mewes H. W. (2010) CORUM: the comprehensive resource of mammalian protein complexes–2009. Nucleic Acids Res. 38, D497–501 19884131 10.1093/nar/gkp914 PMC2808912

[38] Stark C., Breitkreutz B. J., Reguly T., Boucher L., Breitkreutz A., and Tyers M. (2006) BioGRID: a general repository for interaction datasets. Nucleic Acids Res. 34, D535–539 16381927 10.1093/nar/gkj109 PMC1347471

[39] Sharma V., Eckels J., Taylor G. K., Shulman N. J., Stergachis A. B., Joyner S. A., Yan P., Whiteaker J. R., Halusa G. N., Schilling B., Gibson B. W., Colangelo C. M., Paulovich A. G., Carr S. A., Jaffe J. D., MacCoss M. J., and MacLean B. (2014) Panorama: a targeted proteomics knowledge base. J. Proteome Res. 13, 4205–4210 25102069 10.1021/pr5006636 PMC4156235

[40] Ross P. L., Huang Y. N., Marchese J. N., Williamson B., Parker K., Hattan S., Khainovski N., Pillai S., Dey S., Daniels S., Purkayastha S., Juhasz P., Martin S., Bartlet-Jones M., He F., Jacobson A., and Pappin D. J. (2004) Multiplexed protein quantitation in Saccharomyces cerevisiae using amine-reactive isobaric tagging reagents. Mol. Cell. Proteomics 3, 1154–1169 15385600 10.1074/mcp.M400129-MCP200

[41] Karp P. D., Riley M., Saier M., Paulsen I. T., Collado-Vides J., Paley S. M., Pellegrini-Toole A., Bonavides C., and Gama-Castro S. (2002) The EcoCyc Database. Nucleic Acids Res. 30, 56–58 11752253 10.1093/nar/30.1.56 PMC99147

[42] Rolland T., Tasan M., Charloteaux B., Pevzner S. J., Zhong Q., Sahni N., Yi S., Lemmens I., Fontanillo C., Mosca R., Kamburov A., Ghiassian S. D., Yang X., Ghamsari L., Balcha D., Begg B. E., Braun P., Brehme M., Broly M. P., Carvunis A. R., Convery-Zupan D., Corominas R., Coulombe-Huntington J., Dann E., Dreze M., Dricot A., Fan C., Franzosa E., Gebreab F., Gutierrez B. J., Hardy M. F., Jin M., Kang S., Kiros R., Lin G. N., Luck K., MacWilliams A., Menche J., Murray R. R., Palagi A., Poulin M. M., Rambout X., Rasla J., Reichert P., Romero V., Ruyssinck E., Sahalie J. M., Scholz A., Shah A. A., Sharma A., Shen Y., Spirohn K., Tam S., Tejeda A. O., Trigg S. A., Twizere J. C., Vega K., Walsh J., Cusick M. E., Xia Y., Barabasi A. L., Iakoucheva L. M., Aloy P., De Las Rivas J., Tavernier J., Calderwood M. A., Hill D. E., Hao T., Roth F. P., and Vidal M. (2014) A proteome-scale map of the human interactome network. Cell 159, 1212–1226 25416956 10.1016/j.cell.2014.10.050 PMC4266588

[43] Landry C. R., Levy E. D., Abd Rabbo D., Tarassov K., and Michnick S. W. (2013) Extracting insight from noisy cellular networks. Cell 155, 983–989 24267884 10.1016/j.cell.2013.11.003

[44] Braun P., Tasan M., Dreze M., Barrios-Rodiles M., Lemmens I., Yu H., Sahalie J. M., Murray R. R., Roncari L., de Smet A. S., Venkatesan K., Rual J. F., Vandenhaute J., Cusick M. E., Pawson T., Hill D. E., Tavernier J., Wrana J. L., Roth F. P., and Vidal M. (2009) An experimentally derived confidence score for binary protein-protein interactions. Nat. Methods 6, 91–97 19060903 10.1038/nmeth.1281 PMC2976677

[45] Estojak J., Brent R., and Golemis E. A. (1995) Correlation of two-hybrid affinity data with in vitro measurements. Mol. Cell. Biol. 15, 5820–5829 7565735 10.1128/mcb.15.10.5820 PMC230834

[46] Yu H., Braun P., Yildirim M. A., Lemmens I., Venkatesan K., Sahalie J., Hirozane-Kishikawa T., Gebreab F., Li N., Simonis N., Hao T., Rual J. F., Dricot A., Vazquez A., Murray R. R., Simon C., Tardivo L., Tam S., Svrzikapa N., Fan C., de Smet A. S., Motyl A., Hudson M. E., Park J., Xin X., Cusick M. E., Moore T., Boone C., Snyder M., Roth F. P., Barabasi A. L., Tavernier J., Hill D. E., and Vidal M. (2008) High-quality binary protein interaction map of the yeast interactome network. Science 322, 104–110 18719252 10.1126/science.1158684 PMC2746753

[47] Murali T., Pacifico S., Yu J., Guest S., Roberts G. G. 3rd, and Finley R. L. Jr. (2011) DroID 2011: a comprehensive, integrated resource for protein, transcription factor, RNA and gene interactions for Drosophila. Nucleic Acids Res. 39, D736–743 21036869 10.1093/nar/gkq1092 PMC3013689

[48] Simonis N., Rual J. F., Carvunis A. R., Tasan M., Lemmens I., Hirozane-Kishikawa T., Hao T., Sahalie J. M., Venkatesan K., Gebreab F., Cevik S., Klitgord N., Fan C., Braun P., Li N., Ayivi-Guedehoussou N., Dann E., Bertin N., Szeto D., Dricot A., Yildirim M. A., Lin C., de Smet A. S., Kao H. L., Simon C., Smolyar A., Ahn J. S., Tewari M., Boxem M., Milstein S., Yu H., Dreze M., Vandenhaute J., Gunsalus K. C., Cusick M. E., Hill D. E., Tavernier J., Roth F. P., and Vidal M. (2009) Empirically controlled mapping of the Caenorhabditis elegans protein-protein interactome network. Nat. Methods 6, 47–54 19123269 10.1038/nmeth.1279 PMC3057923

[49] Gavin A. C., Aloy P., Grandi P., Krause R., Boesche M., Marzioch M., Rau C., Jensen L. J., Bastuck S., Dumpelfeld B., Edelmann A., Heurtier M. A., Hoffman V., Hoefert C., Klein K., Hudak M., Michon A. M., Schelder M., Schirle M., Remor M., Rudi T., Hooper S., Bauer A., Bouwmeester T., Casari G., Drewes G., Neubauer G., Rick J. M., Kuster B., Bork P., Russell R. B., and Superti-Furga G. (2006) Proteome survey reveals modularity of the yeast cell machinery. Nature 440, 631–636 16429126 10.1038/nature04532

[50] Krogan N. J., Cagney G., Yu H., Zhong G., Guo X., Ignatchenko A., Li J., Pu S., Datta N., Tikuisis A. P., Punna T., Peregrín-Alvarez J. M., Shales M., Zhang X., Davey M., Robinson M. D., Paccanaro A., Bray J. E., Sheung A., Beattie B., Richards D. P., Canadien V., Lalev A., Mena F., Wong P., Starostine A., Canete M. M., Vlasblom J., Wu S., Orsi C., Collins S. R., Chandran S., Haw R., Rilstone J. J., Gandi K., Thompson N. J., Musso G., St Onge P., Ghanny S., Lam M. H. Y., Butland G., Altaf-Ul A. M., Kanaya S., Shilatifard A., O'Shea E., Weissman J. S., Ingles C. J., Hughes T. R., Parkinson J., Gerstein M., Wodak S. J., Emili A., and Greenblatt J. F. (2006) Global landscape of protein complexes in the yeast Saccharomyces cerevisiae. Nature 440, 637–643 16554755 10.1038/nature04670

[51] Guruharsha K. G., Rual J.-F., Zhai B., Mintseris J., Vaidya P., Vaidya N., Beekman C., Wong C., Rhee D. Y., Cenaj O., McKillip E., Shah S., Stapleton M., Wan K. H., Yu C., Parsa B., Carlson J. W., Chen X., Kapadia B., VijayRaghavan K., Gygi S. P., Celniker S. E., Obar R. A., and Artavanis-Tsakonas S. (2011) A protein complex network of Drosophila melanogaster. Cell 147, 690–703 22036573 10.1016/j.cell.2011.08.047 PMC3319048

[52] Malovannaya A., Lanz R. B., Jung S. Y., Bulynko Y., Le N. T., Chan D. W., Ding C., Shi Y., Yucer N., Krenciute G., Kim B.-J., Li C., Chen R., Li W., Wang Y., O'Malley B. W., and Qin J. (2011) Analysis of the human endogenous coregulator complexome. Cell 145, 787–799 21620140 10.1016/j.cell.2011.05.006 PMC3131083

