# Supplementary material for: Quantitative Tagless Copurification: A Method to Validate and Identify Protein-Protein Interactions
Source: Mol Cell Proteomics. 2016 Apr 20;15(6):2186–202. doi: 10.1074/mcp.M115.057117 (PMC5083090; doi:10.1074/mcp.M115.057117)
Supplement: Supplemental Data [file supp_15_6_2186__index.html]

Quantitative tagless co-purification: a method to validate and identify protein-protein interactions — Quantitative Tagless Copurification: A Method to Validate and Identify Protein-Protein Interactions — Tagless Validation and Identification — Supplemental Data 

# Quantitative Tagless Copurification: A Method to Validate and Identify Protein-Protein Interactions

## Supplemental Data

- supplemental information (.pdf, 2.6 MB) - supplemental information
- Dataset S1 (.xlsx, 13.3 MB) - Dataset S1
- Dataset S2 (.xlsx, 18.9 MB) - Dataset S2
- Dataset S3 (.xlsx, 25.7 MB) - Dataset S3
- Dataset S4 (.xls, 100 KB) - Dataset S4
- Dataset S5 (.xls, 152 KB) - Dataset S5
- Dataset S6 (.xlsx, 6.2 MB) - Dataset S6
- Dataset S7 (.txt, 21.1 MB) - Dataset S7
- Dataset S8 (.txt, 13.4 MB) - Dataset S8
- Dataset S9 (.pdf, 5.9 MB) - Dataset S9
