# Supplementary material for: Quantitative Tagless Copurification: A Method to Validate and Identify Protein-Protein Interactions
Source: Mol Cell Proteomics. 2016 Apr 20;15(6):2186–202. doi: 10.1074/mcp.M115.057117 (PMC5083090; doi:10.1074/mcp.M115.057117)
Supplement: Supplemental Data [file 10.1074_M115.057117_mcp.M115.057117-1.pdf]

SUPPLEMENTAL INFORMATION

Quantitative tagless co-purification: a method to validate and identify protein-protein interactions

Maxim Shatsky[1,#], Ming Dong[2,#], Haichuan Liu[3,#], Lee Lisheng Yang[4], Megan Choi[2], Mary E. Singer[2], Jil T. Geller[2], Susan J. Fisher[3], Steven C. Hall[3], Terry C.Hazen[5,6], Steven E. Brenner[1,7], Gareth Butland[8], Jian Jin[4], H. Ewa Witkowska[3], John-Marc Chandonia[1,*], and Mark D. Biggin[2,*]

[1] Physical Biosciences Division, Lawrence Berkeley National Laboratory, Berkeley, CA 94720, USA

[2] Genomics Division, Lawrence Berkeley National Laboratory, Berkeley, CA 94720, USA

[3] OB/GYN Department, University of California San Francisco-Sandler-Moore Mass Spectrometry Core Facility, University of California, San Francisco, CA 94143, USA

[4] Engineering Division, Lawrence Berkeley National Laboratory, Berkeley, CA 94720, USA

[5] Department of Civil and Environmental Engineering, University of Tennessee, Knoxville, TN 37996, USA

[6] Biosciences Division, Oak Ridge National Laboratory, Oak Ridge, TN 37831, USA

[7] Department of Plant and Microbial Biology, University of California, Berkeley, CA 94720, USA

[8] Life Sciences Division, Lawrence Berkeley National Laboratory, Berkeley, CA 94720, USA

[#]These authors contributed equally to this work.

[*]Corresponding authors: mdbiggin@lbl.gov and JMChandonia@lbl.gov

supplemental Text S1 pages 2-5

supplemental Tables S1-S5 pages 6-9

supplemental Figures S1-S15 pages 10-20

SUPPLEMENTAL TEXT S1

Discussion of individual protein complexes from the combined interactome for *D. vulgaris*

The protein complexes discussed here are part of the combined, high confidence interactome for *D. vulgaris* that includes the 200 PPIs from our tagless screen and the 459 PPIs from our AP-MS dataset (Fig. 6; supplemental Fig. S15; supplemental Dataset S5). This combined interactome contains 599 PPIs. We have previously described protein complexes identified in the AP-MS dataset (1). Here we discuss the additional PPIs that were identified as part of our tagless screen. These interactions can be divided into three classes; i) those that support interactions previously identified by AP-MS; ii) those that provide additional interactions within complexes identified by AP-MS or that link together complexes identified by AP-MS; iii) those that are within complexes determined solely by the tagless method and which in some cases are supported by data other than our AP-MS screen.

i. Tagless PPIs that support interactions previously identified by AP-MS

The 60 PPIs identified by both AP-MS and tagless methods include many for large multi-component protein complexes well characterized in other experiments: e.g. ATP synthase, dissimilatory sulfite reductase (DsrABCD), Qmo oxioreductase (QmoABCD) and RNA polymerase (supplemental Fig. S15 a). Many other interactions that have been characterized in the literature were also observed in both screens, including carbomyl phosphate synthase (CarAB), pyruvate:ferredoxin oxidoreductase (PorAB), the aspartate carbamoyltransferase – dihydroorotase complex (PyrBC), dihydroorotate dehydrogenase and its cognate electron transfer partner subunit (PyrDK), two uncharacterized complexes with homology to pyruvate ferredoxin oxidoreductase (DVU3347-50) and indolepyruvate ferredoxin oxidoreductase (DVU1950-DVU1951) respectively, glutamate synthase (DVU1821-23) and several members of the flavin oxioreductase and heterodisulfide reductase complexes (FlxAB(CD)-HdrABC) (supplemental Fig. S15 a and b).

ii. Tagless PPIs that provide additional interactions within complexes identified by AP-MS or that link together complexes identified by AP-MS.

We identified many examples where interactions predicted by the tagless method built on or linked together *D. vulgaris* complexes identified by AP-MS. One interesting set of such “add-on” interactions involve carbomyl phosphate synthase (CarA-CarB) and members of the two known carbomly phosphate dependent pathways: *de novo* pyrimidine biosynthesis and arginine biosynthesis (supplemental Fig. S15 b). The tagless method identified interactions between the AP-MS predicted CarAB, PyrB-PyrC-PyrR, and PyrD-PyrK complexes. These linking interactions were not found by AP-MS but are plausible given that PyrB utilizes carbomyl phosphate and acts immediately upstream of PyrC and PyrDK. Strikingly, CarB was also predicted to associate with ArgF and ArgG, which catalyze the transfer of carbomyl phosphate to L-ornithine.

A second series of add-on interactions identified by the tagless method were associated with heme biosynthesis (2). These include interactions with the alternative heme biosynthesis (Ahb) pathway, i.e. the conversion of sirohaem to haem via 12,18-didecarboxysirohaem (DDSH) and Fe-coproporphyrin III catalyzed by AhbAB, AhbC, and AhbD (3, 4). Whereas AP-MS identified interactions between AhbA and AhbB with an uncharacterized protease, suggesting a potential mechanism of post-translational regulation, the tagless method identifies a series of interactions between AhbA, AhbB, AhbC and AhbD (supplemental Fig. S15 b).

A third example where the tagless method extended AP-MS predicted PPIs is among the HypBCDE hydrogenase maturation proteins (supplemental Fig. S15 b). While a HypC-HypD interaction was identified by both AP-MS and tagless, the HypD-HypE and HypB-HypD interactions were only observed using the tagless method. In this case, the HypC-HypD and HypD-HypE interactions are also supported by independent reports in the literature (5, 6).

A fourth example is amongst leucine biosynthesis enzymes (supplemental Fig. S15 b). The LeuCD complex is supported by both our AP-MS and tagless screens as well as previous observations. The tagless method, however, predicts additional interactions between LeuA-LeuC and LeuB-LeuC. Importantly, these interactions are supported by the fact that in *E. coli*, LeuA and LeuB respectively catalyze the enzymatic steps of leucine biosynthesis immediately preceding and following LeuCD (7).

Finally, the tagless method identified additional interactions associated with the Qmo oxioreductase subunit QmoB: namely QmoB-ApsA, QmoB-Sat and Sat-ApsA (supplemental Fig. S15 b). The adenyl sulphate reductase alpha subunit (ApsA) is a component of the 5'-adenylylsulfate (APS) reductase which has been functionally linked to the Qmo complex, and Sat is a sulfate adenylyltransferase which catalyzes the activation of sulfate to form APS (the substrate for ApsAB) and diphosphate (8, 9).

iii. PPIs within complexes determined solely by the tagless method.

Many complexes are defined by only the tagless method and not by our *D. vulgaris* AP-MS screen (1). For example, the tagless method predicts interactions associated with molybdopterin cofactor biosynthesis i.e. MobA-MobB, MobB-MoeA2 and MoeA2-MoeA1 (supplemental Fig. S15 c). These interactions are supported by previous findings in *E. coli* using bacterial two-hybrid screening (10).

A second series of interactions identified only by the tagless method are between DVU0734, a bifunctional Uroporphyrinogen III synthase and methyltransferase, and three "early heme biosynthesis" enzymes preceding it, namely *hemA*, *hemL* and *hemB* (2) (supplemental Fig. S15 c). Expression of a complete set of early heme biosynthesis enzymes from *D. vulgaris* in *E. coli* has been reported to result in the highly efficient production of Precorrin-2, the expected product of DVU0734 (11). The observed interactions could therefore be consistent with substrate channeling of labile biosynthetic intermediates or represent a regulatory mechanism.

A third interesting example identified only by tagless co-purification are interactions associated with the purine biosynthetic pathway. The predicted interactions include PurL-PurQ (aka phosphoribosylformylglycinamidine (FGAM) synthases I and II), PurL-PurM, PurL-PurF and PurM-PurD (supplemental Fig. S15 c). Significantly, most gram-negative bacteria and eukaryotes contain a single, large PurL polypeptide that includes FGAM synthase. In contrast, gram-positive bacteria and archaea largely contain a FGAM synthase composed of three proteins: a smaller version of PurL as well as PurQ and PurS (12, 13). *D. vulgaris* appears to diverge from both of these cases and encodes two proteins, a hybrid PurL (which contains an N-terminal domain homologous to PurS) and PurQ which our data predict associate. Our data also predict that PurL associates with PurM, the aminoimidazole ribonucleotide (AIR) synthase that catalyzes the subsequent step in purine biosynthesis. Our data also predict that PurM itself associates with PurD the glycinamide ribonucleotide synthase which catalyzes a reaction two steps before PurL (7). We note that PurF, the phosphoribosylpyrophosphate (PRPP) amidotransferase which catalyzes the step immediately preceding PurD, is also predicted by our data to associate with PurL, which functions two steps later in the biosynthetic pathway. These

data are consistent with the hypothesis that our predicted PPIs may represent fragments of larger complexes between several of the *de novo* purine biosynthesis enzymes.

Finally, beyond the above the larger, functionally related sets of interactions noted above, a series of binary interactions are also predicted only by the tagless co-purification approach (supplemental Fig. S15 c). These interactions include one between DVU2748(CbiF/CobM) and DVU3169(CbiG), orthologs of which have been reported to form part of an anaerobic vitamin B12 biosynthesis pathway and catalyze the conversion of cobalt-precorrin-4 to cobalt-precorrin-5A, via CbiF, and its subsequent conversion to cobalt-precorrin-5B, via CbiG (14, 15). Additional interactions include one between DVU0823(ArgJ) and DVU2347(ArgD), where ArgJ is a bifunctional enzyme catalyzing the first and fifth reactions of the arginine biosynthetic pathway and ArgD encodes the fourth step of the arginine biosynthetic pathway (16, 17); Flagella proteins FliS and FlaB1 (18); and a formate dehydrogenase alpha subunit DVU2482 (FdnG) with a formate dehydrogenase formation protein DVU0577 (FdhE; (19)). While many of the other interactions detected by only one of our two *D. vulgaris* screens are not supported by other physical or functional evidence, the overall enrichment for high PPI quality scores imply that many of these interactions are also *bona fide* PPIs.

References

[1] Shatsky, M., Allen, S., Gold, B. L., Liu, N. L., Juba, T. R., Reveco, S. A., Elias, D. A., Prathapam, R., He, J., Yang, W., Szakal, E. D., Liu, H., Singer, M. E., Geller, J. T., Lam, B. R., Saini, A., Trotter, V. V., Hall, S. C., Fisher, S. J., Brenner, S. E., Chhabra, S. R., Hazen, T. C., Wall, J. D., Witkowska, H. E., Biggin, M. D., Chandonia, J.-M., and Butland, G. (2016) Bacterial interactomes: interacting protein partners share similar function and are validated in independent assays more frequently than previously reported. *Mol. Cell. Proteomics* **in press**.

[2] Lobo, S. A., Warren, M. J., and Saraiva, L. M. (2012) Sulfate-reducing bacteria reveal a new branch of tetrapyrrole metabolism. *Adv Microb Physiol* **61**, 267-295.

[3] Lobo, S. A., Lawrence, A. D., Romao, C. V., Warren, M. J., Teixeira, M., and Saraiva, L. M. (2014) Characterisation of Desulfovibrio vulgaris haem b synthase, a radical SAM family member. *Biochim Biophys Acta* **1844**, 1238-1247.

[4] Palmer, D. J., Schroeder, S., Lawrence, A. D., Deery, E., Lobo, S. A., Saraiva, L. M., McLean, K. J., Munro, A. W., Ferguson, S. J., Pickersgill, R. W., Brown, D. G., and Warren, M. J. (2014) The structure, function and properties of sirohaem decarboxylase--an enzyme with structural homology to a transcription factor family that is part of the alternative haem biosynthesis pathway. *Mol Microbiol* **93**, 247-261.

[5] Watanabe, S., Matsumi, R., Atomi, H., Imanaka, T., and Miki, K. (2012) Crystal structures of the HypCD complex and the HypCDE ternary complex: transient intermediate complexes during [NiFe] hydrogenase maturation. *Structure* **20**, 2124-2137.

[6] Lacasse, M. J., and Zamble, D. B. (2016) [NiFe]-Hydrogenase Maturation. *Biochemistry*.

[7] Keseler, I. M., Mackie, A., Peralta-Gil, M., Santos-Zavaleta, A., Gama-Castro, S., Bonavides-Martinez, C., Fulcher, C., Huerta, A. M., Kothari, A., Krummenacker, M., Latendresse, M., Muniz-Rascado, L., Ong, Q., Paley, S., Schroder, I., Shearer, A. G., Subhraveti, P., Travers, M., Weerasinghe, D., Weiss, V., Collado-Vides, J., Gunsalus, R. P., Paulsen, I., and Karp, P. D. (2013) EcoCyc: fusing model organism databases with systems biology. *Nucleic Acids Res* **41**, D605-612.

[8] Grein, F., Ramos, A. R., Venceslau, S. S., and Pereira, I. A. (2013) Unifying concepts in anaerobic respiration: insights from dissimilatory sulfur metabolism. *Biochim Biophys Acta* **1827**, 145-160.

[9] Krumholz, L. R., Wang, L., Beck, D. A., Wang, T., Hackett, M., Mooney, B., Juba, T. R., McInerney, M. J., Meyer, B., Wall, J. D., and Stahl, D. A. (2013) Membrane protein complex of APS reductase and Qmo is present in Desulfovibrio vulgaris and Desulfovibrio alaskensis. *Microbiology* **159**, 2162-2168.
[10] Magalon, A., Frixon, C., Pommier, J., Giordano, G., and Blasco, F. (2002) In vivo interactions between gene products involved in the final stages of molybdenum cofactor biosynthesis in Escherichia coli. *J Biol Chem* **277**, 48199-48204.
[11] Lobo, S. A., Brindley, A., Warren, M. J., and Saraiva, L. M. (2009) Functional characterization of the early steps of tetrapyrrole biosynthesis and modification in Desulfovibrio vulgaris Hildenborough. *Biochem J* **420**, 317-325.
[12] Anand, R., Hoskins, A. A., Stubbe, J., and Ealick, S. E. (2004) Domain organization of Salmonella typhimurium formylglycinamide ribonucleotide amidotransferase revealed by X-ray crystallography. *Biochemistry* **43**, 10328-10342.
[13] Morar, M., Hoskins, A. A., Stubbe, J., and Ealick, S. E. (2008) Formylglycinamide ribonucleotide amidotransferase from Thermotoga maritima: structural insights into complex formation. *Biochemistry* **47**, 7816-7830.
[14] Kajiwara, Y., Santander, P. J., Roessner, C. A., Perez, L. M., and Scott, A. I. (2006) Genetically engineered synthesis and structural characterization of cobalt-precorrin 5A and -5B, two new intermediates on the anaerobic pathway to vitamin B12: definition of the roles of the CbiF and CbiG enzymes. *J Am Chem Soc* **128**, 9971-9978.
[15] Moore, S. J., Lawrence, A. D., Biedendieck, R., Deery, E., Frank, S., Howard, M. J., Rigby, S. E., and Warren, M. J. (2013) Elucidation of the anaerobic pathway for the corrin component of cobalamin (vitamin B12). *Proc Natl Acad Sci U S A* **110**, 14906-14911.
[16] Sakanyan, V., Charlier, D., Legrain, C., Kochikyan, A., Mett, I., Pierard, A., and Glansdorff, N. (1993) Primary structure, partial purification and regulation of key enzymes of the acetyl cycle of arginine biosynthesis in Bacillus stearothermophilus: dual function of ornithine acetyltransferase. *J Gen Microbiol* **139**, 393-402.
[17] Xu, Y., Labedan, B., and Glansdorff, N. (2007) Surprising arginine biosynthesis: a reappraisal of the enzymology and evolution of the pathway in microorganisms. *Microbiol Mol Biol Rev* **71**, 36-47.
[18] Wu, L., Wang, J., Tang, P., Chen, H., and Gao, H. (2011) Genetic and molecular characterization of flagellar assembly in Shewanella oneidensis. *PLoS One* **6**, e21479.
[19] Luke, I., Butland, G., Moore, K., Buchanan, G., Lyall, V., Fairhurst, S. A., Greenblatt, J. F., Emili, A., Palmer, T., and Sargent, F. (2008) Biosynthesis of the respiratory formate dehydrogenases from Escherichia coli: characterization of the FdhE protein. *Arch Microbiol* **190**, 685-696.

Dataset[1]	Species[2]	# pairs both detected[3]	# pairs co-occurring[4]	% pairs co-occurring[5]	# of pairs CC>0.85[6]	% pairs CC>0.85[7]	# species pairs both detected[8]	# species pairs CC>0.85[9]	% species pairs CC>0.85[10]	Fold enrichment[11]
**Benchmark**										
EcoCyc	*E. coli*	60	31	51.7%	14	23.3%	170,820	3,941	2.3%	10.1
AP-MS reciprocal	multiple	60	28	46.7%	10	16.7%	172,680	4,000	2.3%	7.2
Y2H reciprocal	multiple	22	11	50%	5	22.7%	273,618	6,493	2.4%	9.6
**Our AP-MS interactomes**										
AP-MS Shatsky	*D. vulgaris*	284	162	57%	79	27.8%	770,661	13,692	1.8%	15.7
AP-MS Hu rev.	*E.coli*	41	23	56.1%	8	19.5%	170,820	3,941	2.3%	8.5
**Non-interacting PPIs**										
Gold negative	*E.coli*	7,074	1,496	21.1%	151	2.1%	170,820	3,941	2.3%	0.9
All possible pairs	*D. vulgaris*	770,661	146,792	19%	13,692	1.8%	770,661	13,693	1.8%	1.0
**Other interactomes**										
AP-MS Hu	*E. coli*	399	124	31.1%	17	4.3%	170,820	3,941	2.3%	1.8
AP-MS Arif.	*E. coli*	478	154	32.2%	29	6.1%	170,820	3,941	2.3%	2.6
AP-MS Kuhner	*M. pneumoniae*	98	43	43.9%	13	13.3%	8,646	270	3.1%	4.2
Y2H Rajagopala	*E. coli*	70	19	27.1%	5	7.1%	170,820	3,941	2.3%	3.1
Y2H Hauser	*H. pylori*	50	11	22.0%	5	10%	68,265	1,985	2.9%	3.4
Y2H Marchadier	*B. subtilis*	25	4	16.0%	1	4%	134,421	3,531	2.6%	1.5
Y2H Titz	*T. pallidum*	95	24	25.3%	2	2.1%	24,531	629	2.6%	0.8
Y2H Parrish	*C. jejuni*	1595	515	32.3%	59	3.7%	94,830	3,076	3.2%	1.1
Y2H Sato	*Synechocystis*	66	15	22.7%	2	3%	125,250	3,759	3%	1.0

Table S1. Enrichment of highly correlated, co-occurring protein pairs among different datasets. Information is shown, from top to bottom, for three benchmark PPI datasets; our two AP-MS interactomes; two sets of largely non-interacting protein pairs; and the nine other published bacterial interactomes (Experimental Procedures). Note that in this case PPIs from our *D. vulgaris* AP-MS interactome have been excluded from the AP-MS reciprocal set. The columns show from left to right [1] the name and type of dataset; [2] the species; [3] the number of protein pairs for which homologs of both proteins are detected among the 1,242 protein in the tagless fractions; [4] the number of pairs defined in column 3 that co-occur in at least one multiplex; [5] the percent of pairs defined in column 3 that co-occur in at least one multiplex; [6] the number of pairs defined in column 3 that have maximum CC values >0.85 in both the HIC and SEC dimensions; [7] the percent of pairs defined in column 3 that have a maximum CC values >0.85 in both the HIC and SEC dimensions; [8] the number interologs of the species for which both proteins are detected among the 1,242 protein in the tagless fractions, without regard to any evidence that the pair interact; [9] the number of pairs defined in column 8 that have maximum CC values >0.85 in both the HIC and SEC dimensions; [10] the percentage of pairs defined in column 8 that have maximum CC values >0.85 in both the HIC and SEC dimensions; [11] the ratio of the percentages in column 7 / column 10.

Dataset[1]	# protein pairs[3]	% GS positives[2]	% GS negatives[3]	% same operon[4]	Fold same TIGR role[5]	% pairs in DvH AP-MS[6]	% pairs in other species AP-MS[7]	% pairs in other species Y2H[8]
**Benchmark**								
EcoCyc	1,549	NA	NA	54.0%	10	48% (24/50)	14% (122/884)	11% (77/670)
AP-MS Reciprocals	389	15%	6.8%	29%	6.4	62%	19%	22%
Y2H Reciprocals	224	12%	3.2%	18%	6.5	81%	44%	57%
**Tagless MS only**								
top 51	51	12%	0%	25%	6.6	85% (17/20)	29% (4/14)	22% (5/23)
top 51 and MS+STRING top 200	18	33%	0%	72%	12	100% (16/16)	67% (4/6)	50% (5/10)
top 51 not MS+STRING top 200	33	0%	0%	0%	2.2	25% (1/4)	NA (0/0)	0% (0/13)
top 52-201	150	0%	0.67%	4%	1.6	26% (7/27)	0% (0/42)	2% (1/51)
**Tagless MS+STRING**								
top 200	200	9%	2.5%	46%	8.8	65% (60/93)	18% (15/85)	17% (17/99)
top 201–300	100	0%	2%	16%	7.6	16% (4/25)	9.1% (5/55)	0% (0/53)

Table S2. PPI metric scores for benchmark datasets and high and lower confidence *D. vulgaris* tagless protein pair sets. The top three rows show metrics for benchmark PPI datasets and the remaining rows show metrics for different subsets of protein pairs from our analysis of the *D. vulgaris* tagless dataset. Note that here the reciprocal AP-MS benchmark includes PPIs from our *D. vulgaris* AP-MS interactome. The columns give from left to right: [1] the dataset name; [2] the percent of protein pairs in our gold positive set; [3] the percent of protein pairs in our gold negative set; [4] the percent of protein pairs whose members are encoded in the same operon; [5] the fold enrichment of protein pairs for which both members have the same TIGR role over than expected among randomly chosen pairs of proteins; [6] the percent of protein pairs identified by AP-MS in *D. vulgaris*, except in the case of the AP-MS reciprocal PPIs, where same study protein pairs are excluded; [7] The percent of protein pairs that are also found in the *E. coli* and *M. pneumoniae* AP-MS datasets except in the case of the AP-MS reciprocal PPIs, where same study protein pairs are excluded; [8] The percent of protein pairs whose homologs are also found in the sum of all six Y2H datasets, except in the case of the Y2H reciprocal PPIs, where same study protein pairs are excluded.

Study[1]	Species[2]	# pairs in high conf. set[3]	# proteins in high conf. set (percent of all proteins)[4]	% of high conf. set proteins in dimers[5]	Connected component size (std)[6]
**Benchmark**					
EcoCyc	*E. coli*	1,549	710 (17%)	20%	3.9 (4.45)
**Our interactomes**					
Tagless	*D. vulgaris*	200	262 (8%)	40%	3.1 (2.4)
AP-MS	*D. vulgaris*	459	469 (14%)	36%	3.39 (8.33)
Combined	*D. vulgaris*	599	585 (17%)	28%	4.4 (16)
AP-MS Hu revised	*E. coli*	391	386 (9.3%)	35%	3.6 (5.6)
**Previous interactomes**					
AP-MS Hu	*E. coli*	5,993	1,757 (42%)	4%	47 (271)
AP-MS Arifuzzaman	*E. coli*	11,172	2,962 (71%)	0.1%	740 (1,279)
AP-MS Kuhner	*M. pneumoniae*	1,058	410 (60%)	0.1%	137 (190)
Y2H Titz	*T. pallidum*	979	578 (56%)	2%	72 (185)
Y2H Parrish	*C. jejuni*	2926	1108 (68%)	1%	48 (220)
Y2H Marchadier	*B. subtilis*	704	278 (7%)	3%	56 (107)
Y2H Rajagopala	*E. coli*	1776	1218 (29%)	10%	8.7 (81)
Y2H Hauser	*H. pylori*	728	526 (33%)	9%	18 (84)
Y2H Sato	*Synechocystis*	736	915 (26%)	25%	4.8 (22)

Table S3. The connectivity of proposed bacterial interactomes vs that of EcoCyc PPIs. PPI data are taken from the EcoCyc database; our studies of *D. vulgaris* and *E. coli* and published interactomes for other studies (Experimental Procedures). The columns give from left to right: [1] the name of the study; [2] the species; [3] the number of protein pairs in the high confidence network; [4] the total number of proteins that are in high confidence pairs, i.e. excluding baits not part of any high confidence pair (the percent of high confidence protein pairs vs the total proteome); [5] the percent of proteins that are part of a high confidence protein pair that are linked to only one other protein; [6] the mean size of connected components within the high confidence interactome, i.e. excluding proteins not participating in a high confidence pair.

BioGrid dataset	Havugimana et al., Tagless–only PPIs		Havugimana et al., High confidence PPIs	
	Gold positive PPIs (4,143)	***Novel protein pairs (31,813)***	***Gold positive PPIs (4,596)***	***Novel protein pairs (9,395)***
AP-MS (53,338)	16.99%	0.68%	23.47%	2.17%
Y2H (26,108)	3.76%	0.39%	5.07%	1.61%
Other physical (46,525)	8.36%	0.5%	14.7%	2.6%
All (114,754)	21.02%	0.98%	29.84%	3.56%

Table S4 overlap between BioGrid databases and Havugimana et al.'s interactome. The top three rows are for sets of PPIs derived from the BioGrid Databases for AP-MS experiments, Y2H experiments and other physical interaction studies. The non-redundant union of these three databases was used to calculated the values in the bottom row. The percent of protein pairs in portions of Havugimana et al's interactome are given in the four right hand columns. The tagless-only and high confidence PPIs were each divided into those found in the gold positive datasets used during training to identify that set, and those remaining, novel protein pairs. The number of protein pairs in each dataset are given in brackets.

BioGrid dataset	Wan et al., High confidence PPIs	
	Gold positive PPIs (4,176)	***Novel protein pairs (12,479)***
AP-MS (53,338)	27.44%	3.29%
Y2H (26,108)	6.41%	1.81%
Other physical (46,525)	18.95%	3.94%
All (114,754)	35.41%	5.15%

Table S5 overlap between BioGrid databases and Wan et al's interactome. The rows are as described in Table S4. The percent of protein pairs in Wan et al's interactome are given in the two right hand columns. The number of protein pairs in each dataset are given in brackets.

SUPPLEMENTAL FIGURES

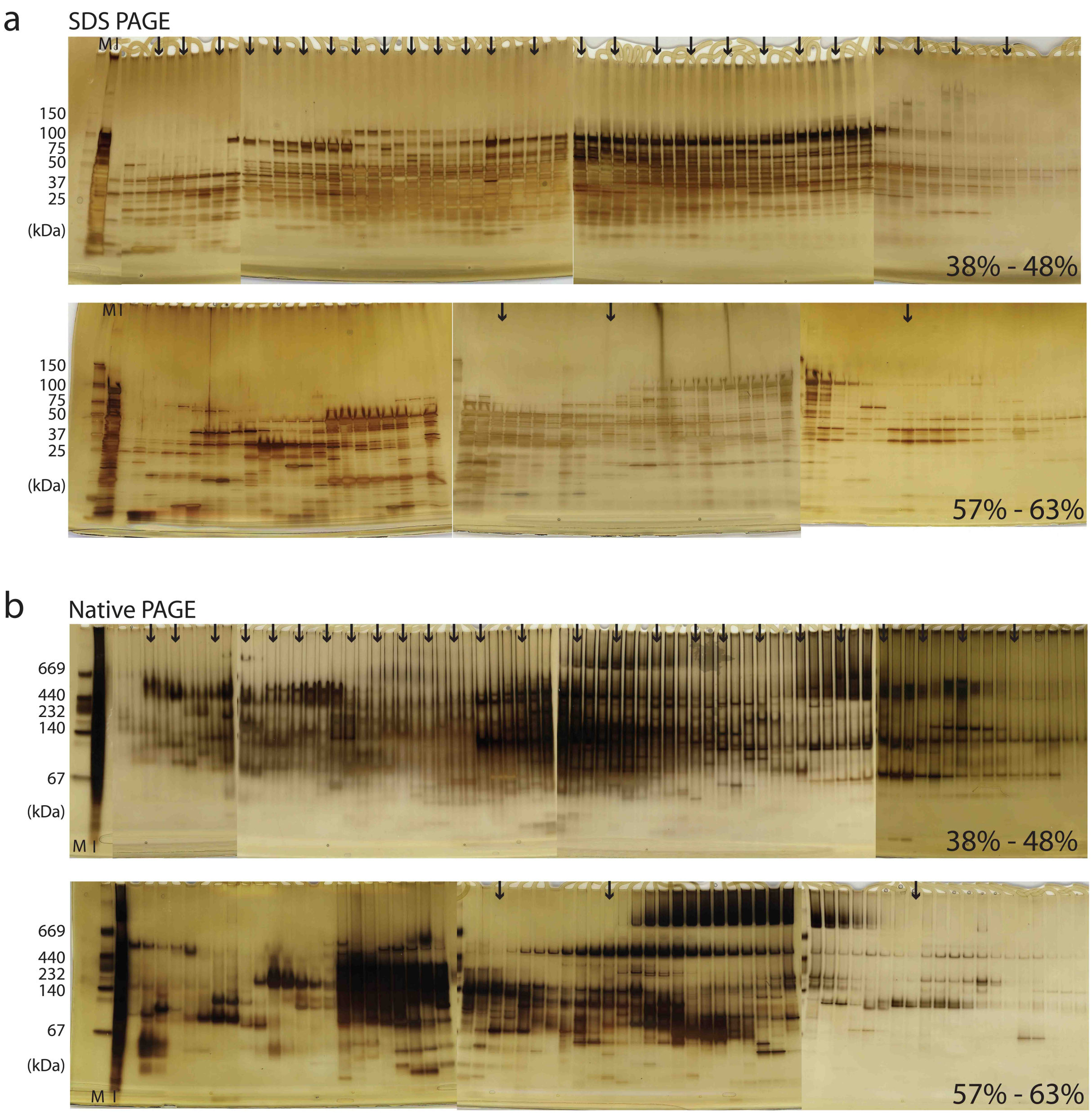

Figure S1. The proteins in Q-IEC fractions. Fractions from mono Q Ion Exchange Chromatography (Q-IEC) columns used to separate 38%-48% and 57%-63% ammonium sulfate cuts. (a) Proteins separated by SDS PAGE (4-20% acrylamide). (b) Proteins separated by native PAGE (4-15% acrylamide). Arrows show the fractions subsequently fractionated by Hydrophobic Interaction Chromatography (HIC).

(a)

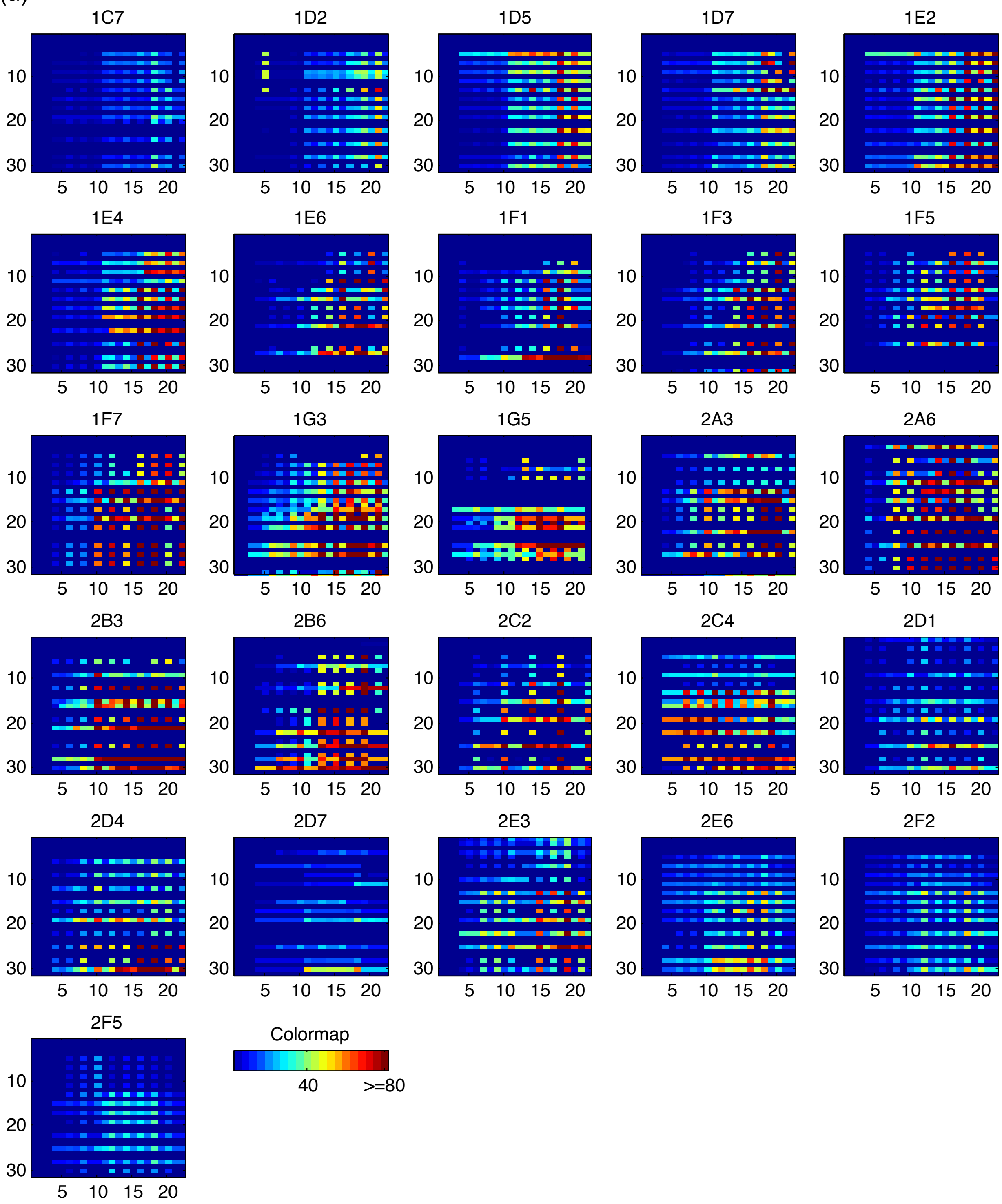

1C7
1D2
1D5
1D7
1E2
1E4
1E6
1F1
1F3
1F5
1F7
1G3
1G5
2A3
2A6
2B3
2B6
2C2
2C4
2D1
2D4
2D7
2E3
2E6
2F2
2F5
Colormap
40
>=80

(b)

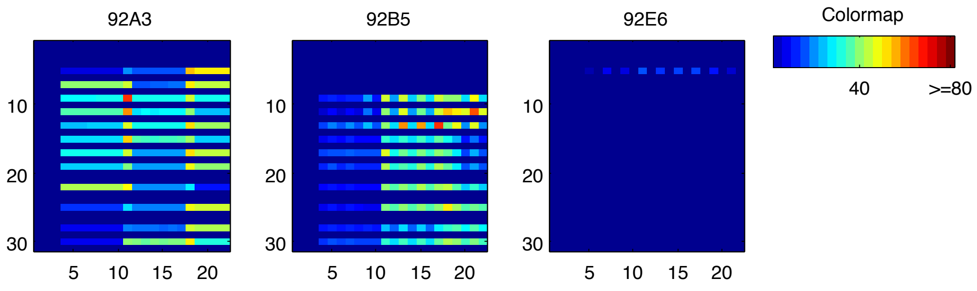

Figure S2. Fraction space analyzed. Each Q-IEC fraction is represented in one block, e.g. 2F5. Each block shows information for the HIC (y-axis) and SEC (x-axis) dimensions derived from a given Q-IEC fraction. The color bar shows the number of proteins found in each SEC fraction. (a) The fractionation of the 38% - 48% Ammonium Sulphate cut. (b) The fractionation of the 53% – 57% Ammonium Sulphate cut.

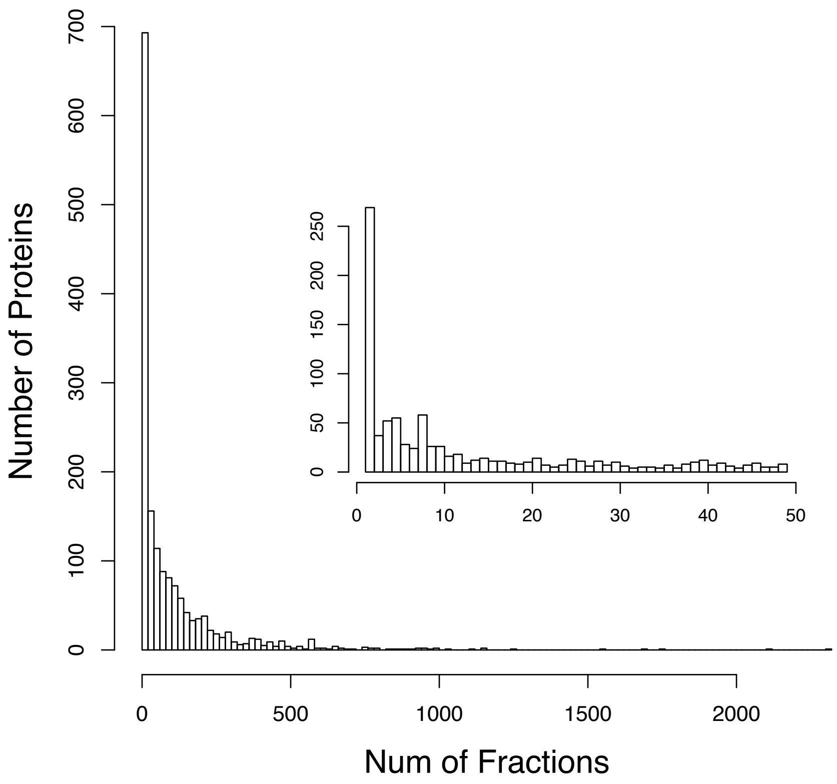

Figure S3. Histogram of the number of fractions a given protein appears in. 61 proteins were detected in at least 500 of the 5,273 tagless fractions assayed, while 915 proteins appeared in less than 50 fractions.

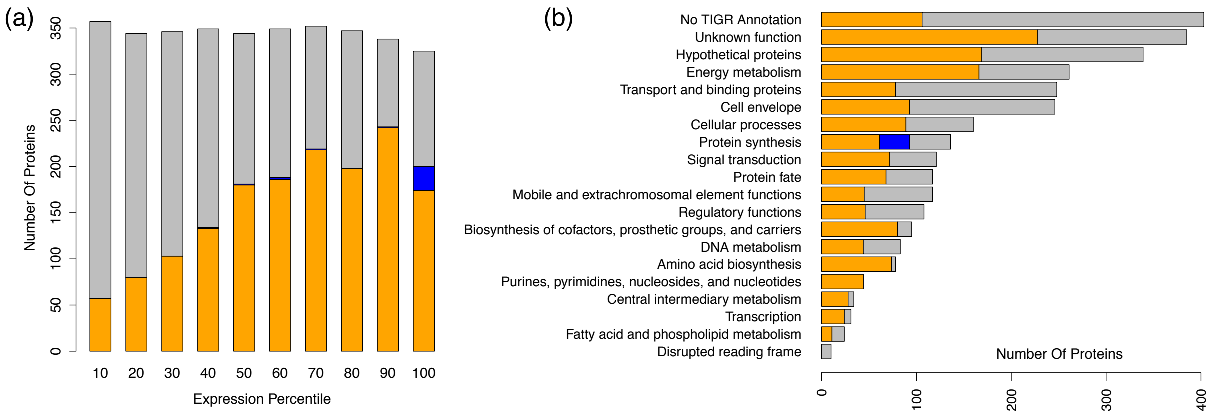

Figure S4. mRNA expression levels and TIGR role annotation of mass spectrometry identified proteins from the tagless fractionation. (a) The shaded sections of each bar show the number of genes encoding non ribosomal proteins successfully fractionated and detected by MS (orange), ribosomal proteins detected by MS (blue), and all other *D. vulgaris* protein-encoding genes not detected in the MS data (grey). The data are plotted as a function of mRNA microarray expression percentile, with highly expressed genes to the right and genes expressed at low or undetectable levels to the left. **(b)** TIGR roles of MS detected proteins (orange), MS detected ribosomal proteins (blue), and all other *D. vulgaris* proteins (grey). The mRNA microarray expression data are from Price et al. who grew *D. vulgaris* under similar conditions (Price et al. (2011) *Journal of Bacteriology* **193**, 5716-5727).

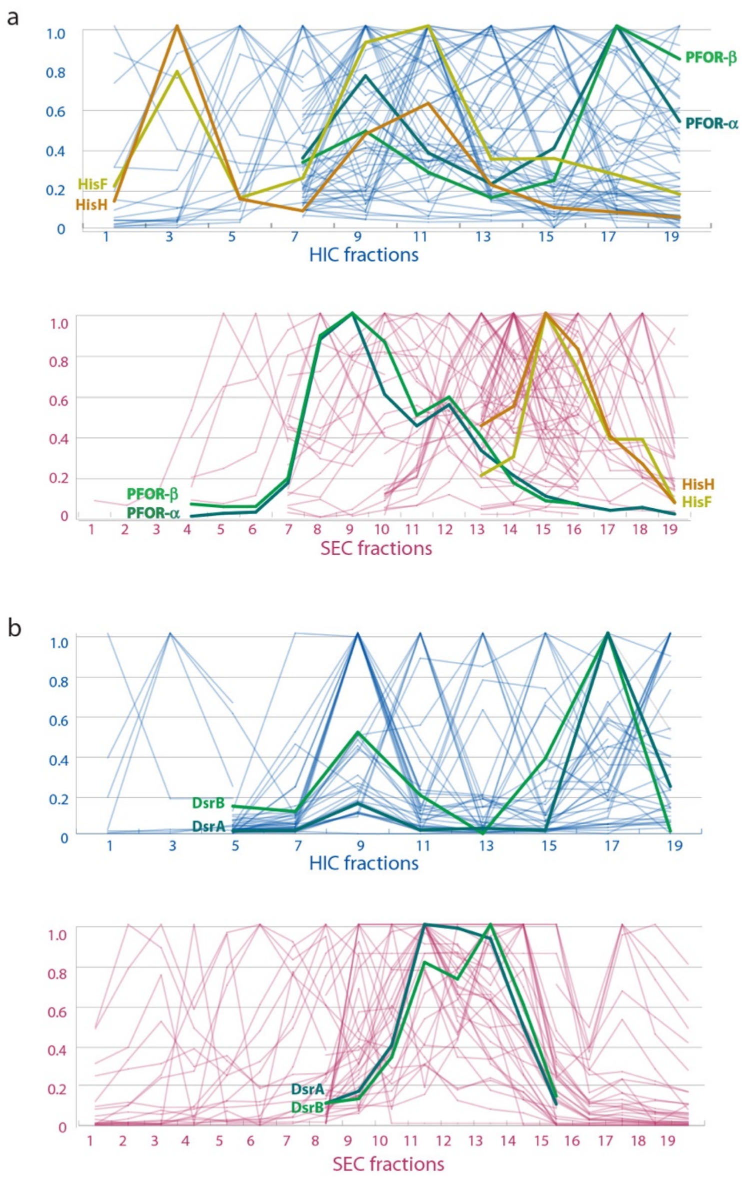

Figure S5. Example iTRAQ elution profiles of proteins across the HIC and SEC dimensions. Profiles of known members of protein complexes are shown in bold lines. The profiles of all other proteins detected are shown in narrow red lines (SEC dimension) or narrow blue lines (HIC dimension). **(a)** Shows the cofractionation of two members of the imidazole glycerol phosphate synthase complex (HisH and HisF) and two members of the pyruvate ferredoxin/flavodoxin oxidoreductase complex (PROF–α and PFOR–β). **(b)** Shows the cofractionation of two members of the dissimilatory sulfite reductase complex (DsrA and DsrB). Panel (a) shows HIC and SEC dimensions from a single mono Q fraction; panel (b) shows HIC and SEC dimensions from a different MonoQ fraction.

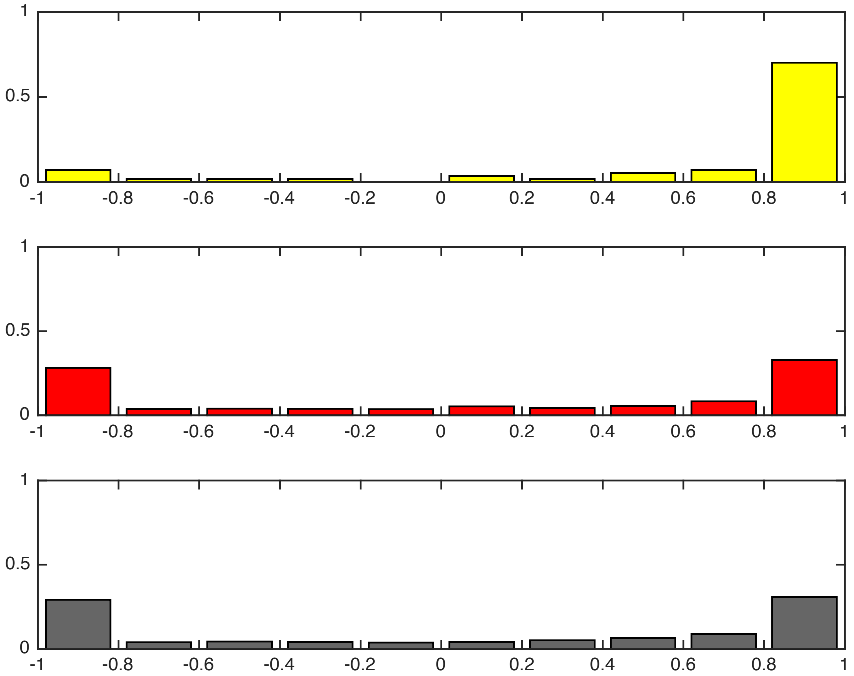

Figure S6. Distribution of max CC values along SEC dimension (feature (1)) (x-axis) for Gold Positive set (yellow), Gold Negative set (red), and for all pairs (grey).

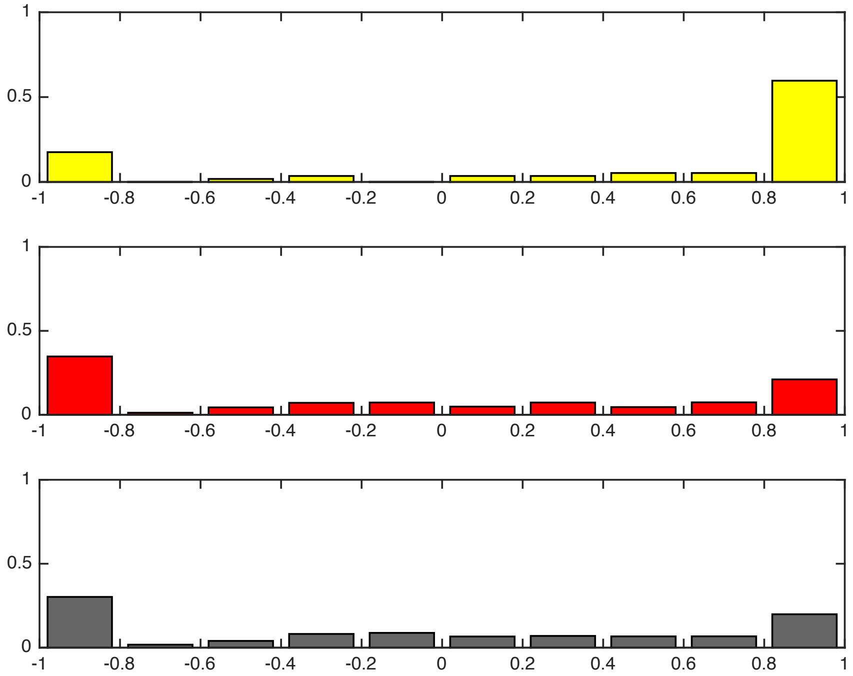

Figure S7. Distribution of max CC values along HIC dimension (feature (2)) (x-axis) for Gold Positive set (yellow), Gold Negative set (red), and for all pairs (grey).

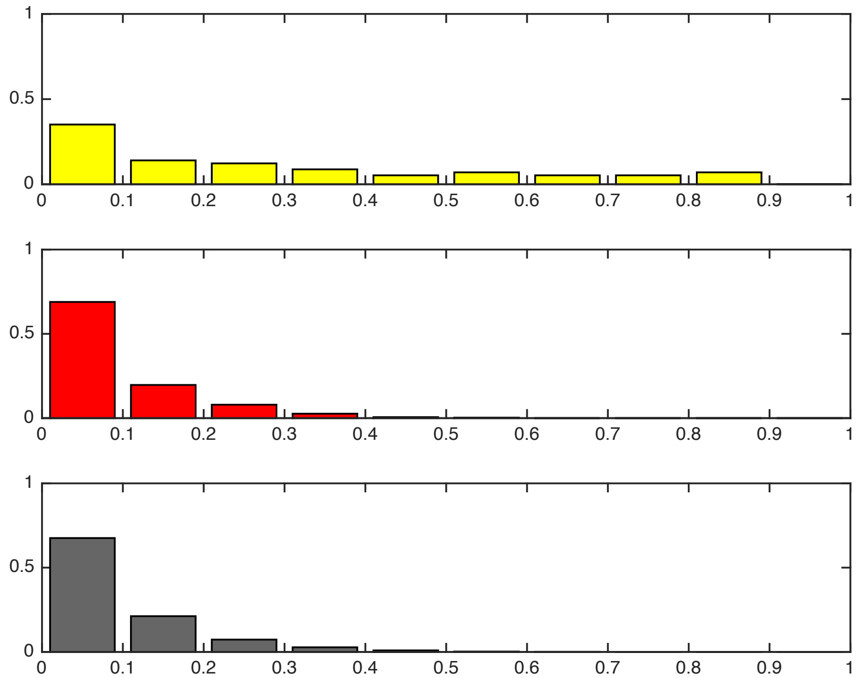

Figure S8. Distribution of dice's coefficients (feature (3)) (x-axis) for Gold Positive set (yellow), Gold Negative set (red), and for all pairs (grey).

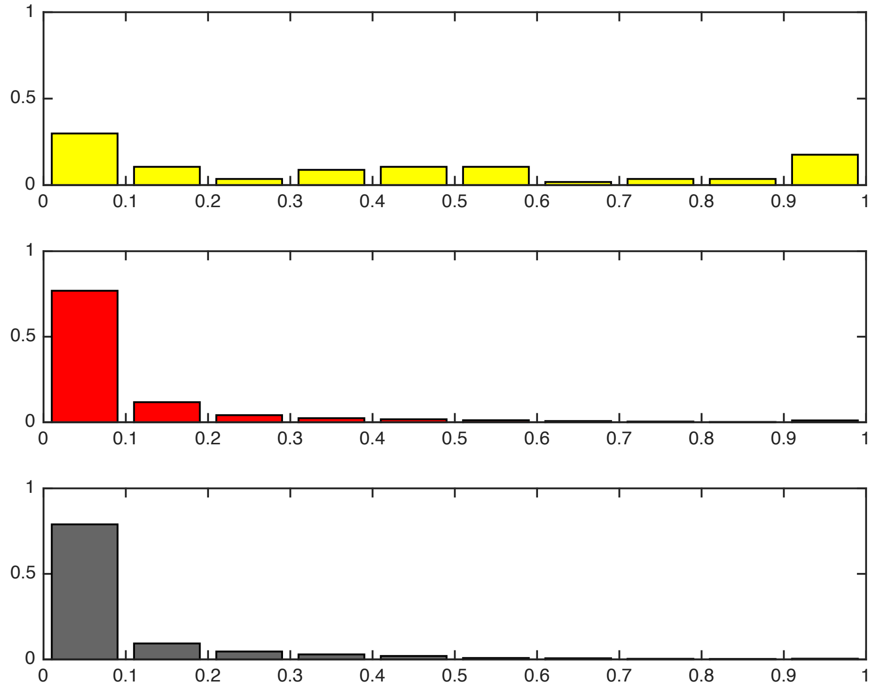

Figure S9. Distribution of peptide ratios (feature (4)) (x-axis) for Gold Positive set (yellow), Gold Negative set (red), and for all pairs (grey).

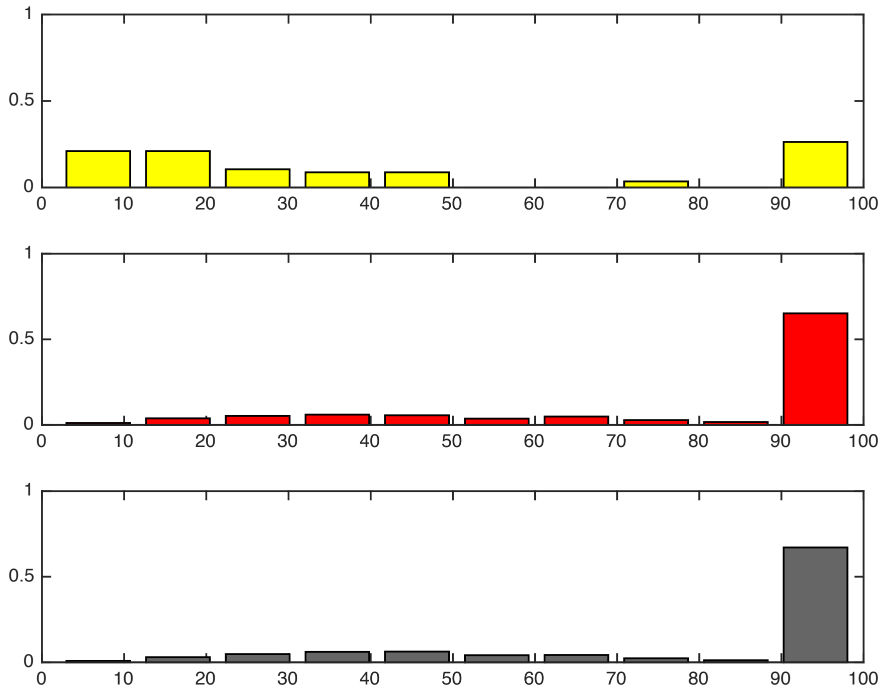

Figure S10. Distribution of minimal number of proteins found in a multiplex (x-axis) (feature (5)) for Gold Positive set (yellow), Gold Negative set (red), and for all pairs (grey).

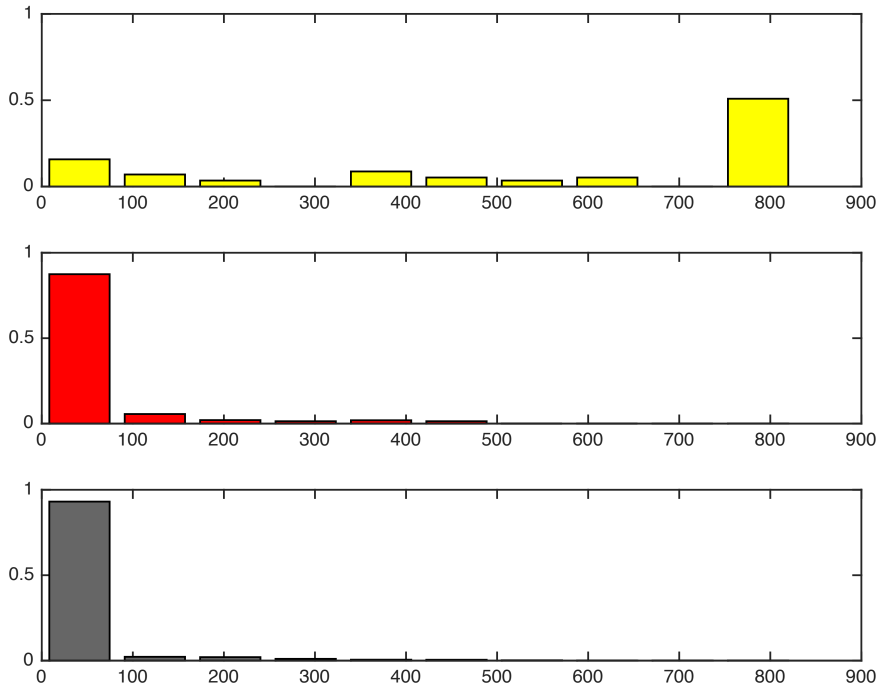

Figure S11. Distribution of neighborhood STRING enrichment scores (x-axis) (feature (6)) for Gold Positive set (yellow), Gold Negative set (red), and for all pairs (grey).

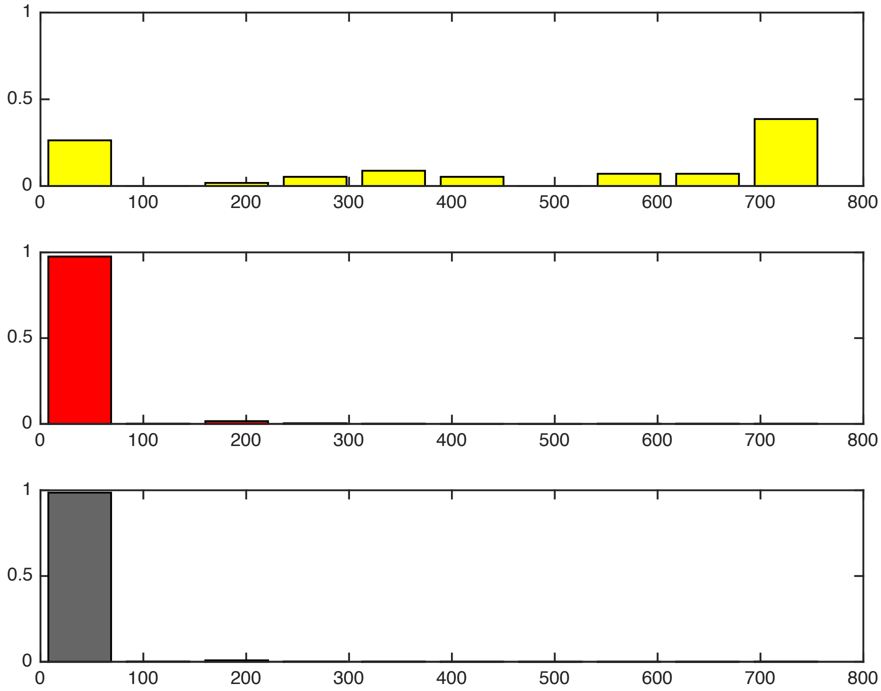

Figure S12. Distribution of co-occurrence STRING enrichment scores (x-axis) (feature (7)) for Gold Positive set (yellow), Gold Negative set (red), and for all pairs (grey).

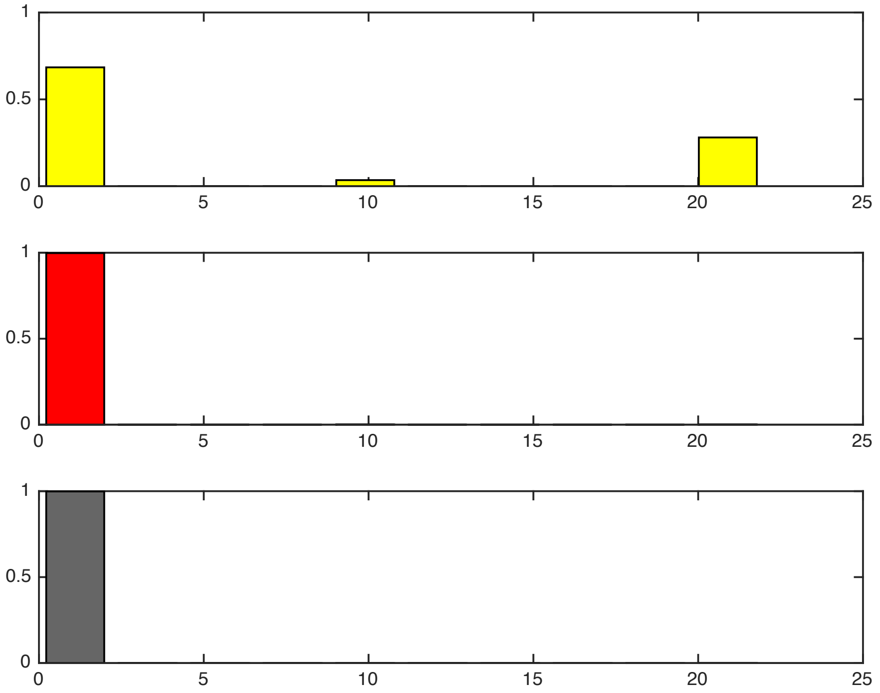

Figure S13. Distribution of fusion STRING enrichment scores (feature (8)) (x-axis) for Gold Positive set (yellow), Gold Negative set (red), and for all pairs (grey).

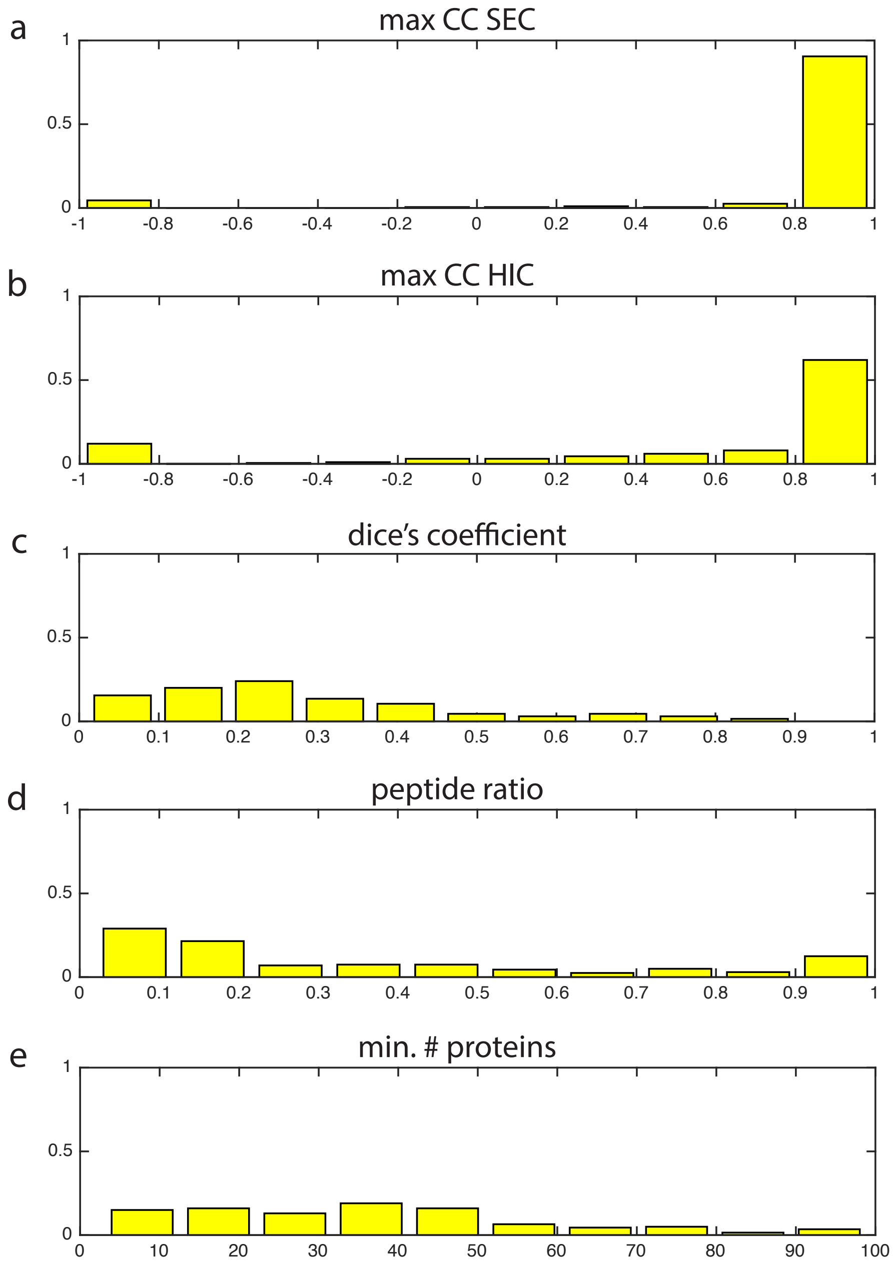

Figure S14. Distribution of the five MS only regression features among the 200 high confidence tagless PPIs. The distributions of regression feature scores (x-axis) are shown for **(a)** The maximum CC value in the SEC dimension (feature (1)). **(b)** The maximum CC value in the HIC dimension (feature (2)). **(c)** Dice's coefficient (feature (3)). **(d)** Peptide ratios (feature (4)). **(e)** Minimal number of proteins found in a multiplex (feature (5)).

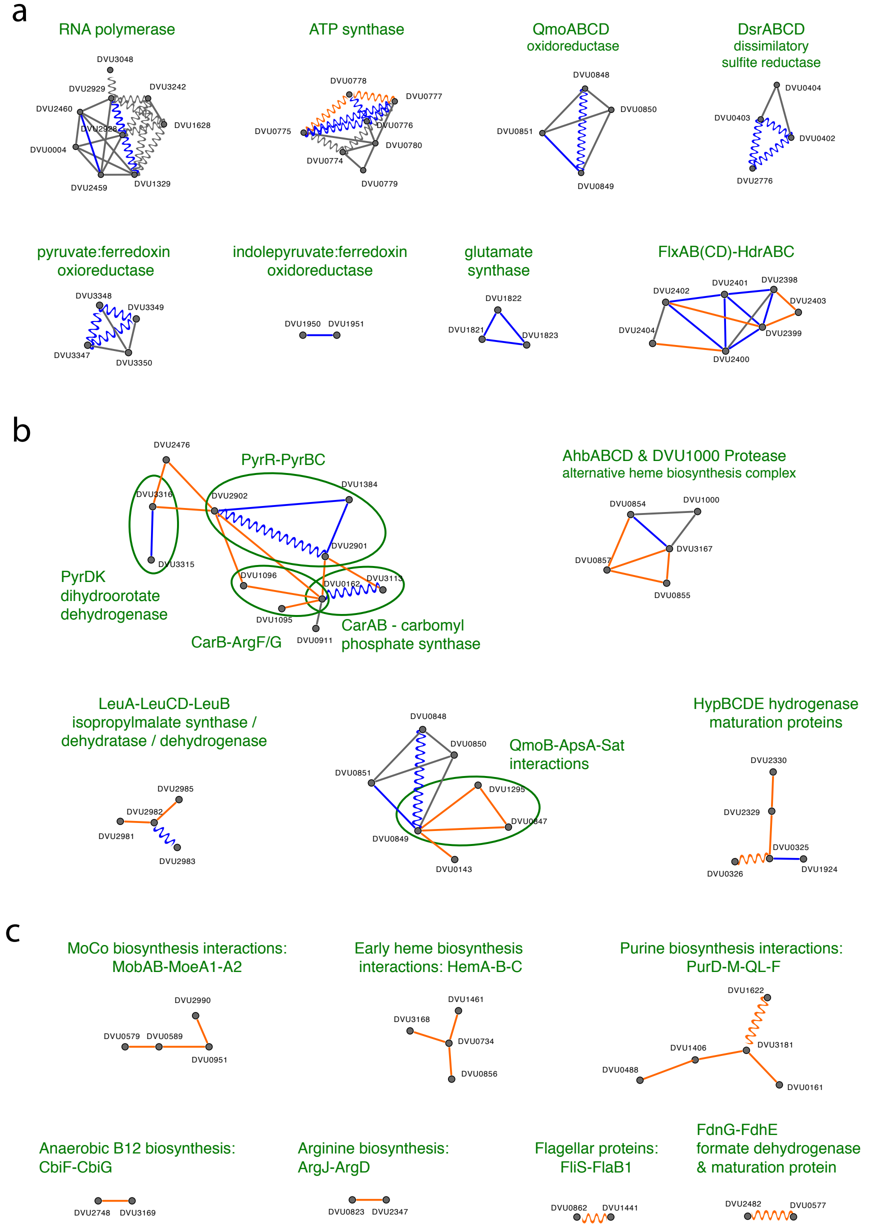

Figure S15. Selected protein complexes from the combined, high confidence D. vulgaris interactome. PPIs in both the AP-MS and tagless interactomes are shown in blue; PPIs only present in the tagless interactome are shown in orange; and PPIs only in the AP-MS interactome are shown in grey. PPIs also supported by additional evidence from gold standard positives or from AP-MS or Y2H screens in other bacteria are shown by wavy lines. **(a)** Complexes that include tagless PPIs that support interactions previously identified by AP-MS. **(b)** Tagless PPIs that provide additional interactions within complexes identified by AP-MS or that link together complexes identified by AP-MS. **(c)** Complexes not supported by our AP-MS screen.
