# Supplementary material for: Quantitative Tagless Copurification: A Method to Validate and Identify Protein-Protein Interactions
Source: Mol Cell Proteomics. 2016 Apr 20;15(6):2186–202. doi: 10.1074/mcp.M115.057117 (PMC5083090; doi:10.1074/mcp.M115.057117)
Supplement: Supplemental Data [file 10.1074_M115.057117_mcp.M115.057117-10.pdf]

Dataset S9

MS/MS Spectra for Proteins Identified on the Basis of Single Peptide

Shatsky et al.

Legend

- Spectra representing proteins identified on the basis of single peptide that are not included in the Skyline spectral library are shown.
- Annotated spectra were extracted from ProteinPilot™ result (.group) files.
- The following information is provided at the top of the spectra: protein name_peptide sequence; m/z value; modifications; missed cleavages; non-tryptic cleavages, if present; match confidence (with 0.99 being the highest possible). Alternative matches for the same spectrum are reported, if applicable. The last lane identifies the source .group file.
- Spectra that are annotated as representing "Competitor Proteins" refer to proteins that were identified on the basis spectral evidence for which multiple sequence matches are possible (with identical or very similar confidence). For those cases, an information on a primary match is provided underneath.
- The cases when the same peptide sequence is present in multiple proteins are annotated as "Multiple IDs".
- Annotation of *y*- and *b*-type product ions is provided.

Competitor Protein: DVU0139_LDEMER

m/z 1096.612 iTRAQ8plex@0; confidence 0.984

Primary ID: DVU0964A_IDLFER

2D1_C10_C12_S5_278_279_06092009_(2_3_2).group

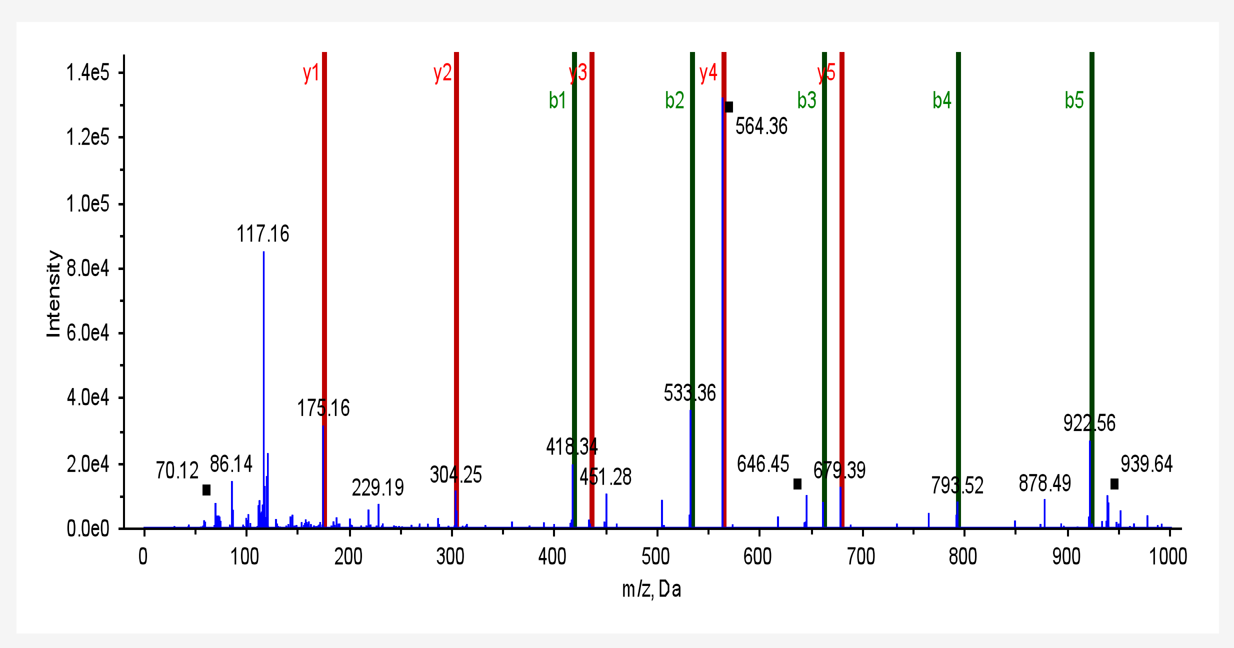

Residue	b	y
L	418.30	1096.56
D	533.32	679.27
E	662.37	564.24
M	793.41	435.20
E	922.45	304.16
R	1078.55	175.12

DVU0152_SLIDFFR

m/z 1201.624 iTRAQ8plex@0; cleaved M-S@N-term confidence 0.9697

1F5_D4_2A6_B11_S5_216&231_03-19-09_(2_3_8).group

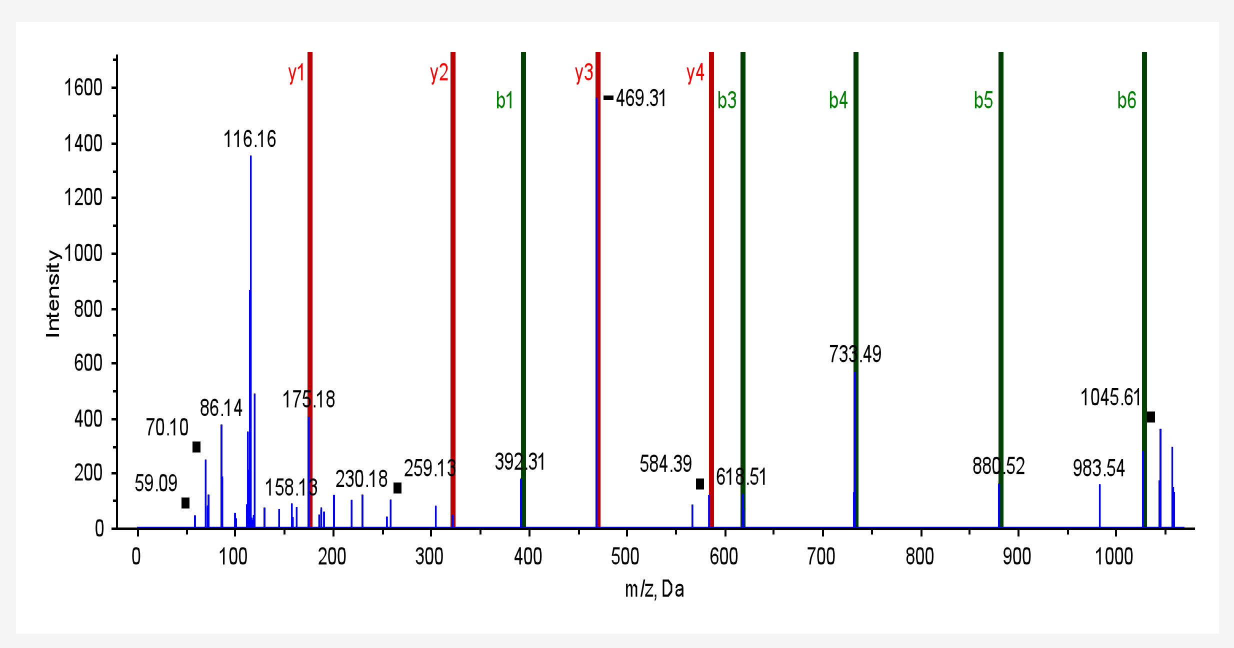

Residue	b	y
S	392.24	1201.69
L	505.33	810.45
I	618.41	697.37
D	733.44	584.28
F	880.51	469.26
F	1027.58	322.19
R	1183.68	175.12

Multiple IDs for [L/I][L/I]SK

m/z 1068.722 iTRAQ8plex@4;iTRAQ8plex@0; semitryptic; confidence 0.9766

DVU0156, DVU0299, DVU0922, DVU0950, DVU1237, DVU1244, DVU2016, DVU2090, DVU2243, DVU2309, DVUA0041, DVUA0138, DVUA0143

S5_381-383_8plex_1-3_02-09-10_(2_3_3).group

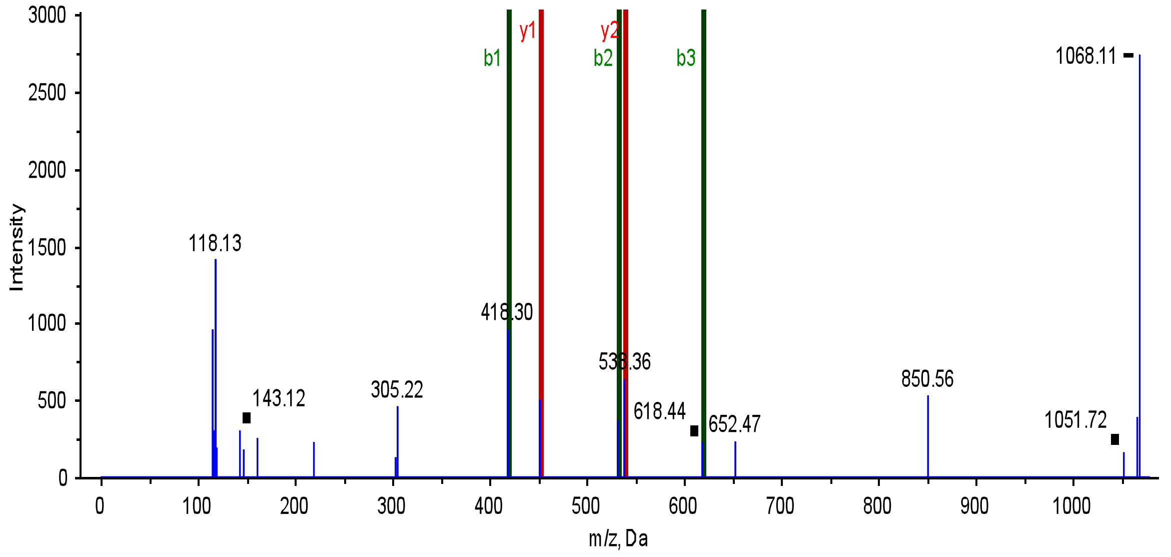

Residue	b	y
L	418.30	1068.72
I	531.38	651.43
S	618.41	538.35
K[IT8]	1050.71	451.32

Competitor Protein: DVU0244_IMSTVR

m/z 1010.594 iTRAQ8plex@0; confidence 0.9622

Primary ID: DVU3294A_LAFTVR

S5_144_reun_01-10-2009_(2_3_5)_(2_3_5).group

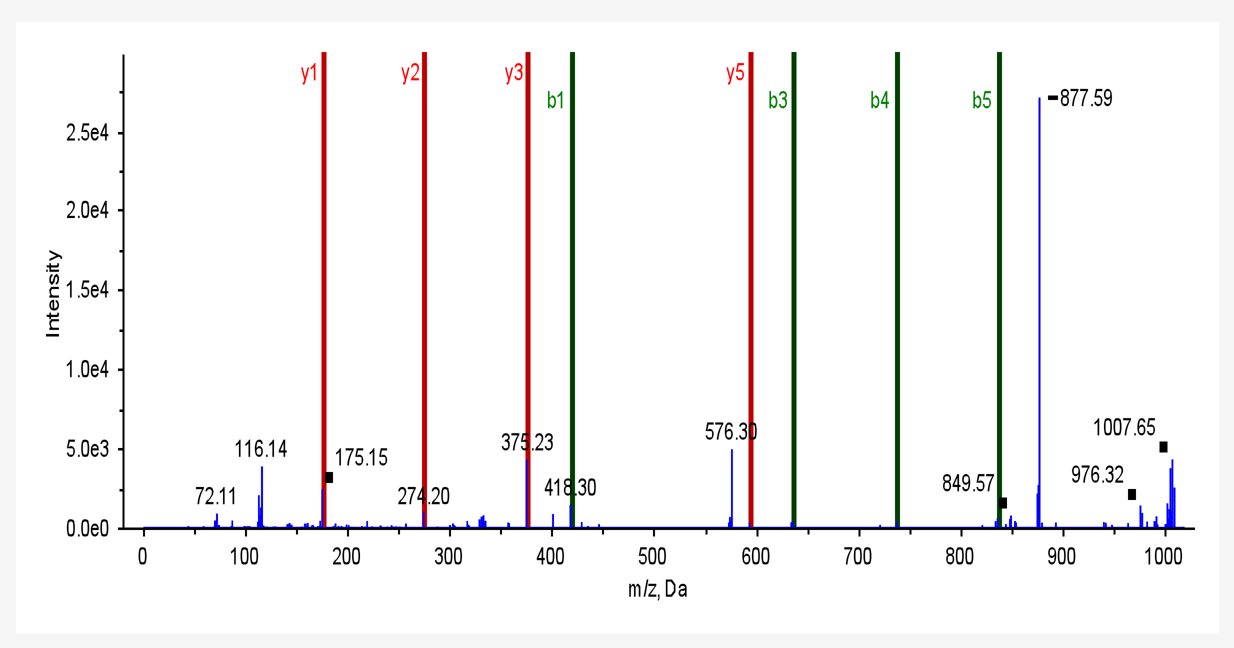

Residue	b	y
I	418.30	1010.60
M	549.34	593.31
S	636.37	462.27
T	737.42	375.24
V	836.49	274.19
R	992.59	175.12

Multiple IDs for YDLL

DVU0545, DVU0667, DVU0692, DVU0700, DVU1660, DVU1866, DVU1958, DVU1968, DVU2277, DVU3132, DVU3174, DVU3193

m/z 827.4309 iTRAQ8plex@0; confidence 0.99

1D5_2D_SEC_B13-BC4_B9_#2_rerun_09242010_(1_2_8)_(2_3_8)_(2_3_5).group

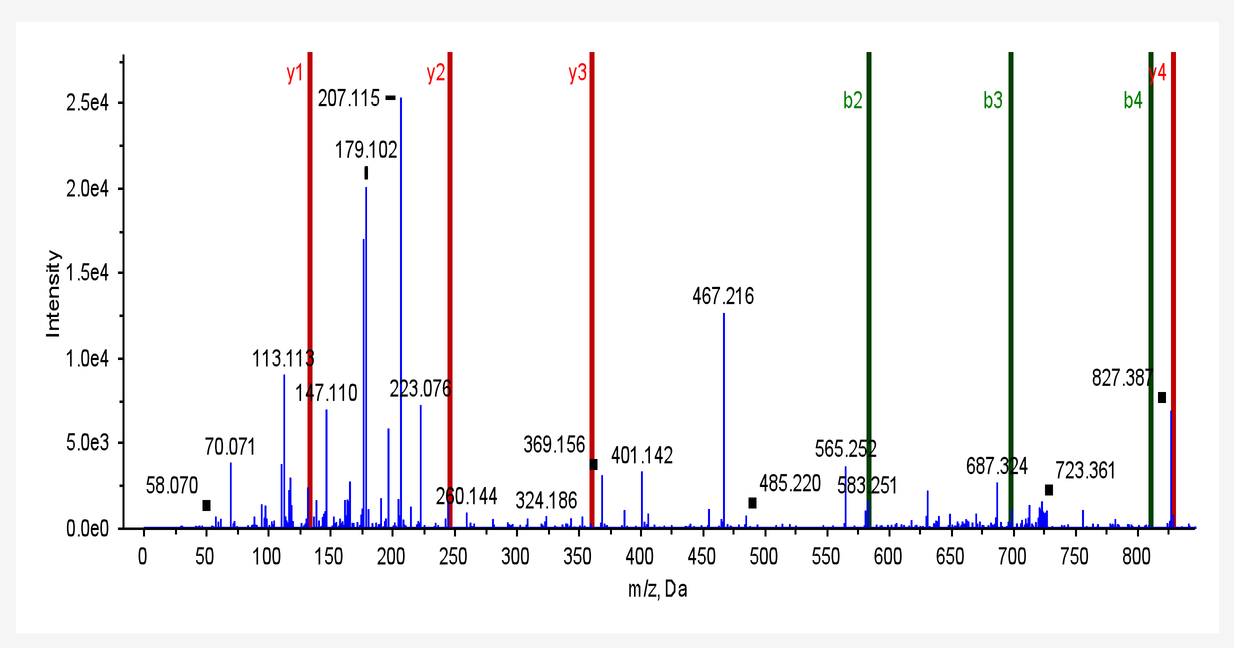

Residue	b	y
Y	468.28	827.48
D	583.30	360.21
L	696.39	245.19
L	809.47	132.10

Competitor Protein: DVU0561_LLDLAR

m/z 1004.645 iTRAQ8plex@0; cleaved W-L@N-term confidence 0.99

Primary ID: DVU1411A_ILDIAR

G3_D14_normal_3-7_mini_7_07-24-08_(2_3_7).group

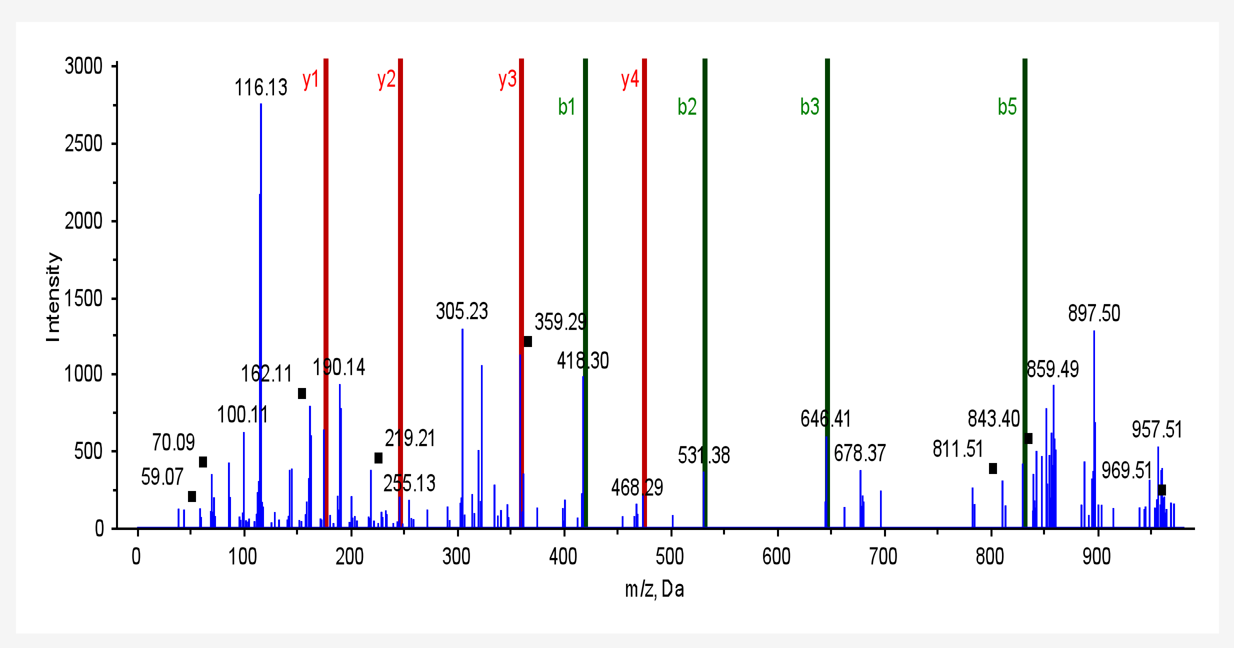

Residue	b	y
L	418.30	1004.64
L	531.38	587.35
D	646.41	474.27
L	759.49	359.24
A	830.53	246.16
R	986.63	175.12

DVU0561_NFDLLLRALR

m/z 1535.842 Deamidated@1;iTRAQ8plex@0; missed R-A@7 confidence 0.99

1D7_2D_tagless_SEC_B3-B11_04082010_(2_3_1).group

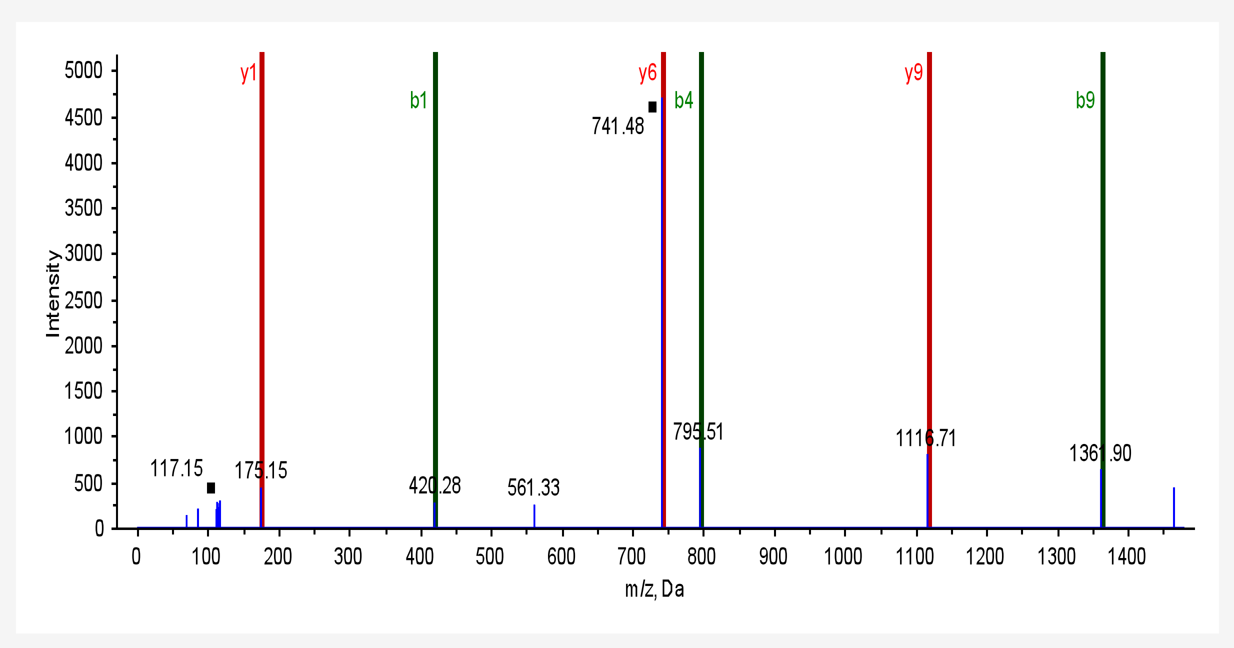

Residue	b	y
N[Dea]	420.24	1535.92
F	567.31	1116.69
D	682.33	969.62
L	795.42	854.59
L	908.50	741.51
L	1021.59	628.43
R	1177.69	515.34
A	1248.73	359.24
L	1361.81	288.20
R	1517.91	175.12

Multiple IDs for LFLLYR:

m/z 1128.754 iTRAQ8plex@0; semitryptic confidence 0.99

DVU0570, DVU0670

2A3_C8_C10_S5_221_222_02-24-2009_(2_3_2)_(2_3_2).group

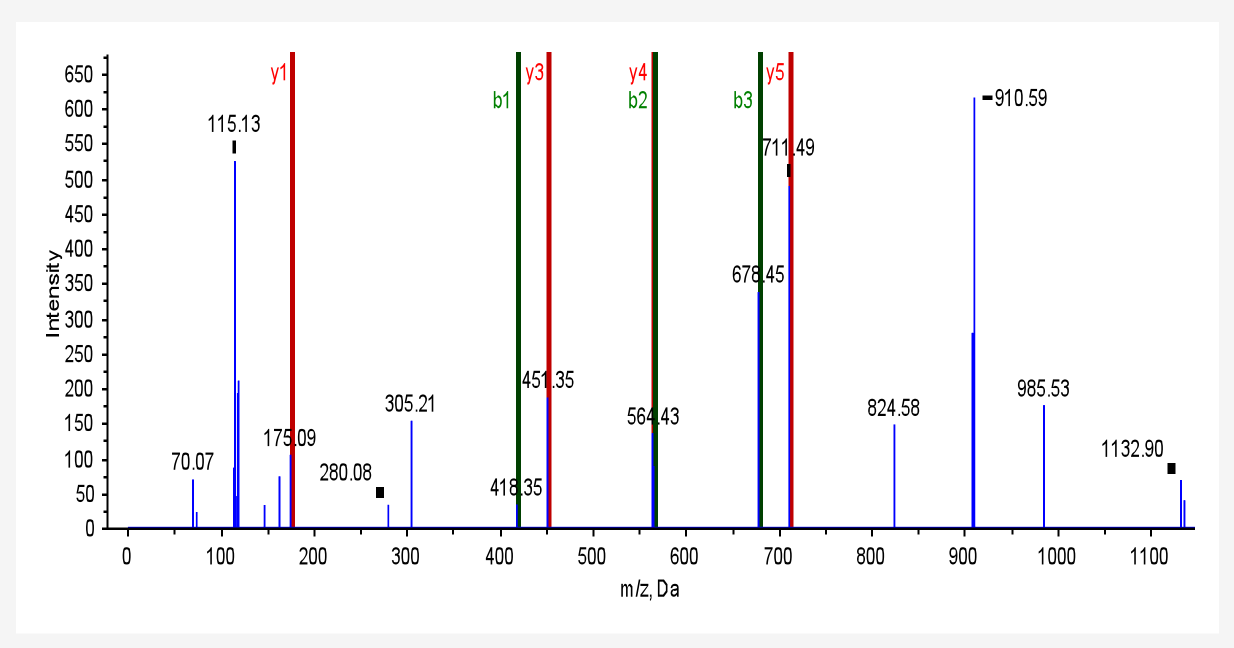

Residue	b	y
L	418.30	1128.71
F	565.37	711.42
L	678.45	564.35
L	791.53	451.27
Y	954.60	338.18
R	1110.70	175.12

Competitor Protein: DVU0608_EAEAARAQAENARR

m/z 1847.933 Deamidated@11;iTRAQ8plex@0; missed R-A@6; missed R-R@13 confidence 0.98

Primary ID: DORF26948_VLEGASRGWCPRR 1F5_C8_C10_S5_211_212_20090219_(3_4_2)_(2_3_2).group

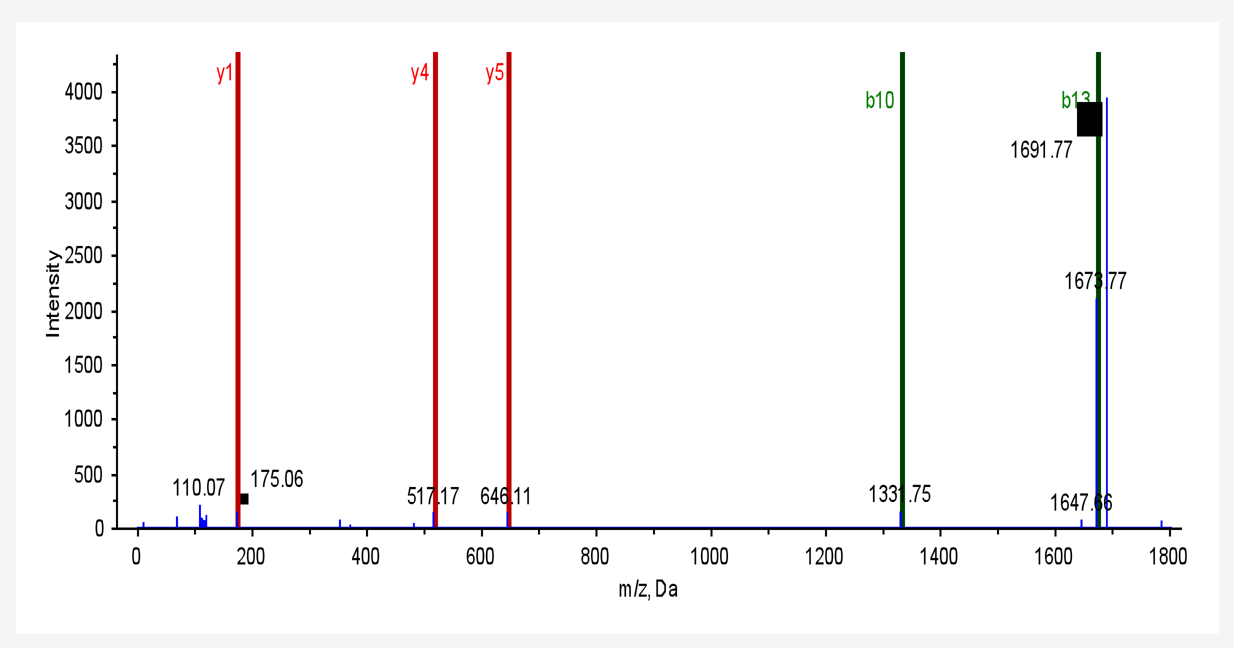

Residue	b	y
E	434.26	1847.96
A	505.29	1414.71
E	634.33	1343.68
A	705.37	1214.63
A	776.41	1143.60
R	932.51	1072.56
A	1003.55	916.46
Q	1131.61	845.42
A	1202.64	717.36
E	1331.69	646.33
N[Dea]	1446.71	517.28
A	1517.75	402.26
R	1673.85	331.22
R	1829.95	175.12

Competitor Protein: DVU0635_LLDGLGAPR

m/z 1215.74 iTRAQ8plex@0; confidence 0.9805

Primary ID: DVU0085A_LIGVEPAGR

MonoQ_2A3_S5_142_2D_SEC_B11-C2_120409_(2_3_7).group

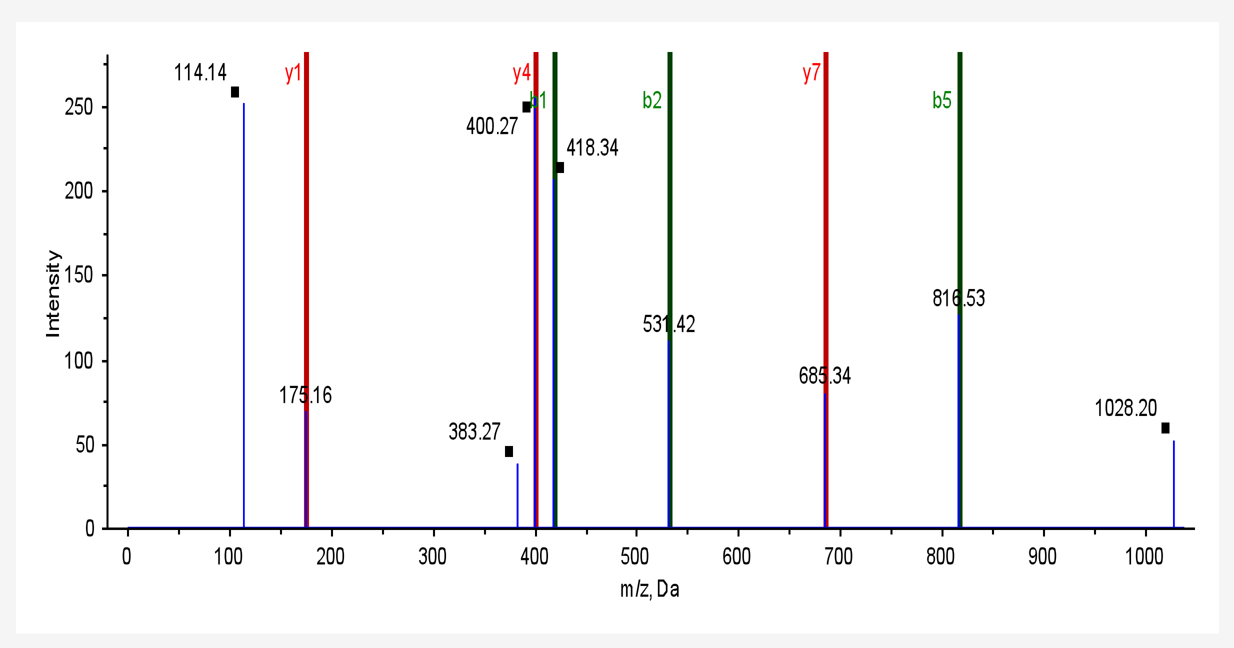

Residue	b	y
L	418.30	1215.74
L	531.38	798.45
D	646.41	685.36
G	703.43	570.34
L	816.51	513.31
G	873.53	400.23
A	944.57	343.21
P	1041.62	272.17
R	1197.73	175.12

Competitor Protein:DVU0653_YRWPGNVR

m/z 1351.755 iTRAQ8plex@0; cleaved A-Y@N-term; missed R-W@2 0.99

Primary ID: DVU2934_YRWPGNVR 2C2_C10_C12_S5_333_331_082509_(3_4_4).group

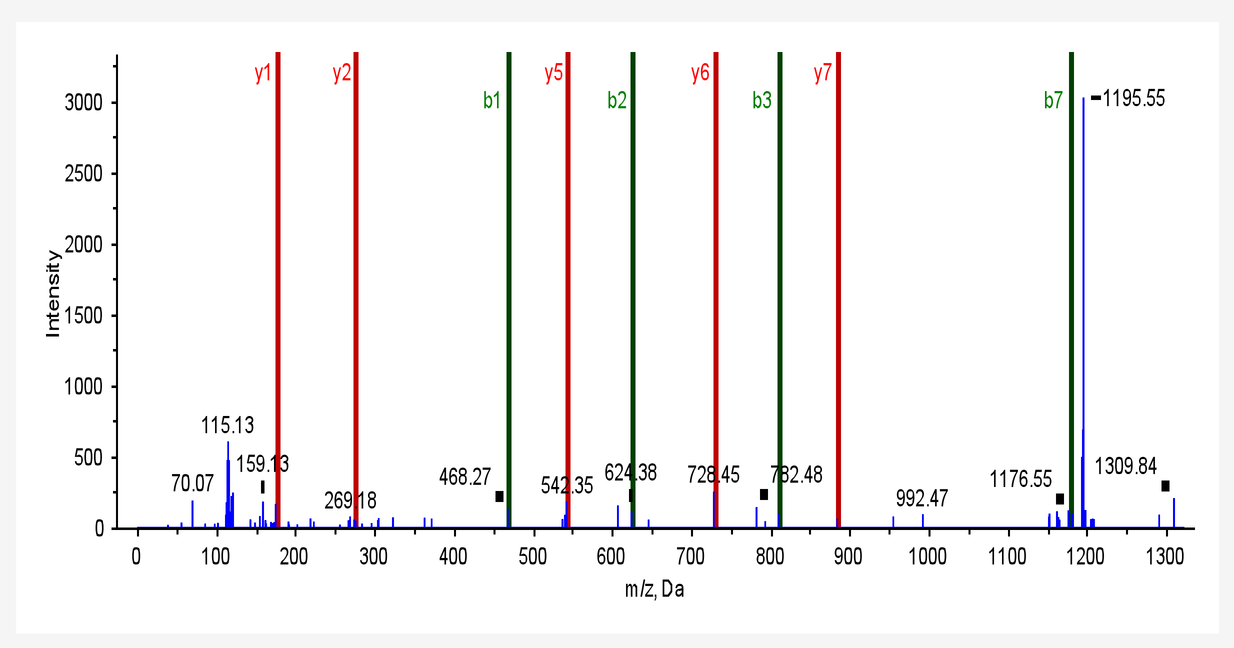

Residue	b	y
Y	468.28	1351.75
R	624.38	884.48
W	810.46	728.38
P	907.51	542.30
G	964.53	445.25
N	1078.57	388.23
V	1177.64	274.19
R	1333.74	175.12

Competitor Protein DVU0680_IDVFR

m/z 953.5557 iTRAQ8plex@0; cleaved A-I@N-term confidence 0.99

Primary ID: DVU1861_ IDVFR

S5_466-468_rerun_zt_111210_(2_3_6).group

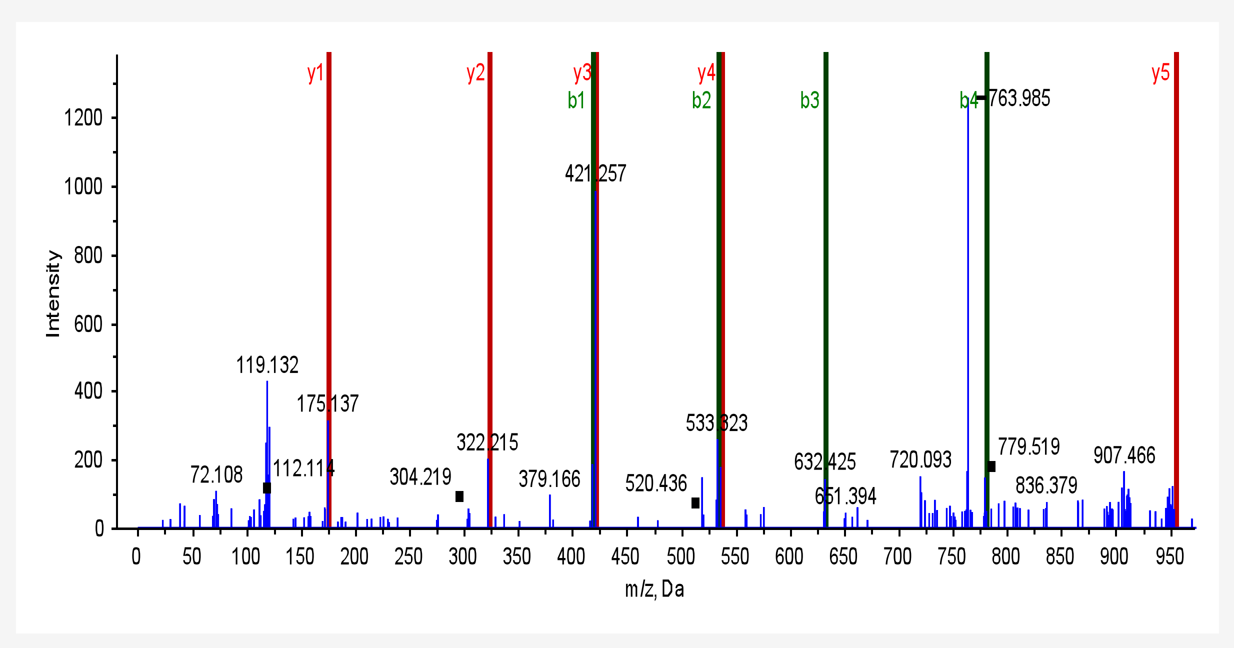

Residue	b	y
I	418.30	953.57
D	533.32	536.28
V	632.39	421.26
F	779.46	322.19
R	935.56	175.12

Competitor Protein:DVU0750_TIDSMR

m/z 1026.59 iTRAQ8plex@0; confidence 0.99

Primary ID: DVU1203_TIDFAR

2A6_B14_C2_S5_232_233_rerun_04-01-09_(2_3_4)_(2_3_5).group

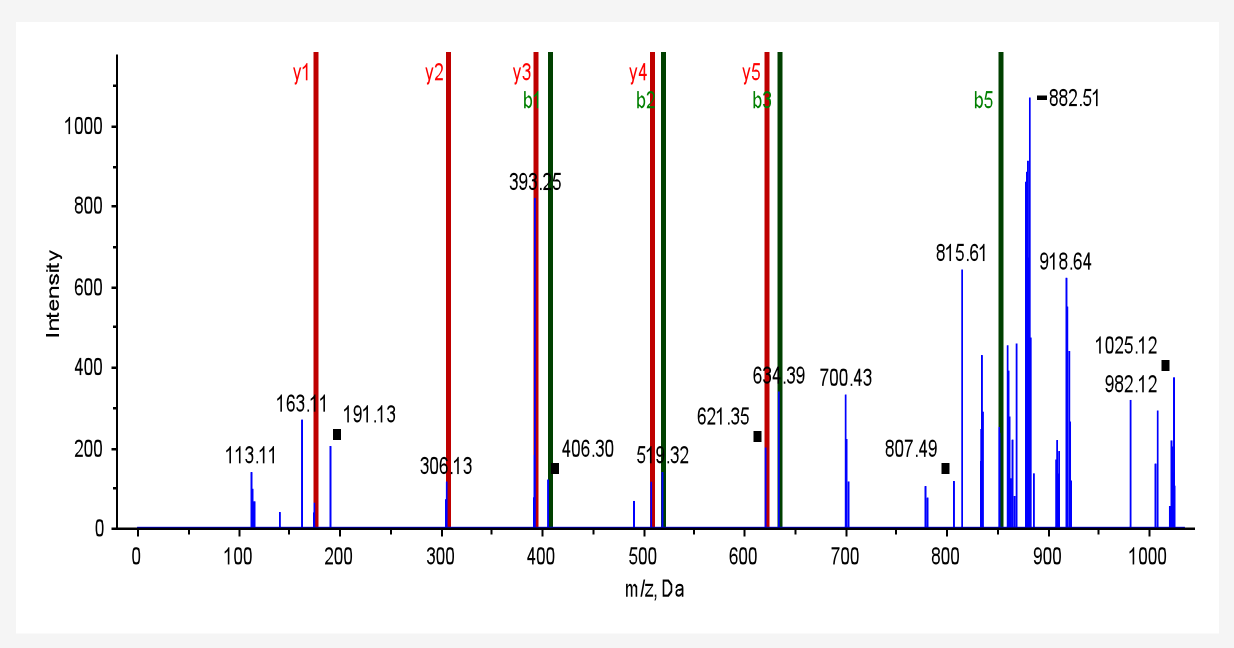

Residue	b	y
T	406.26	1026.56
I	519.34	621.30
D	634.37	508.22
S	721.40	393.19
M	852.44	306.16
R	1008.54	175.12

Competitor Protein: DVU0804_GAFRQDLLYR

m/z 1543.993 Deamidated@5;iTRAQ8plex@0; missed R-Q@4 confidence 0.99

Primary ID: DVU1300_KENLK

2D4_C5_S5_291_relabeled_092509_(2_3_12).group

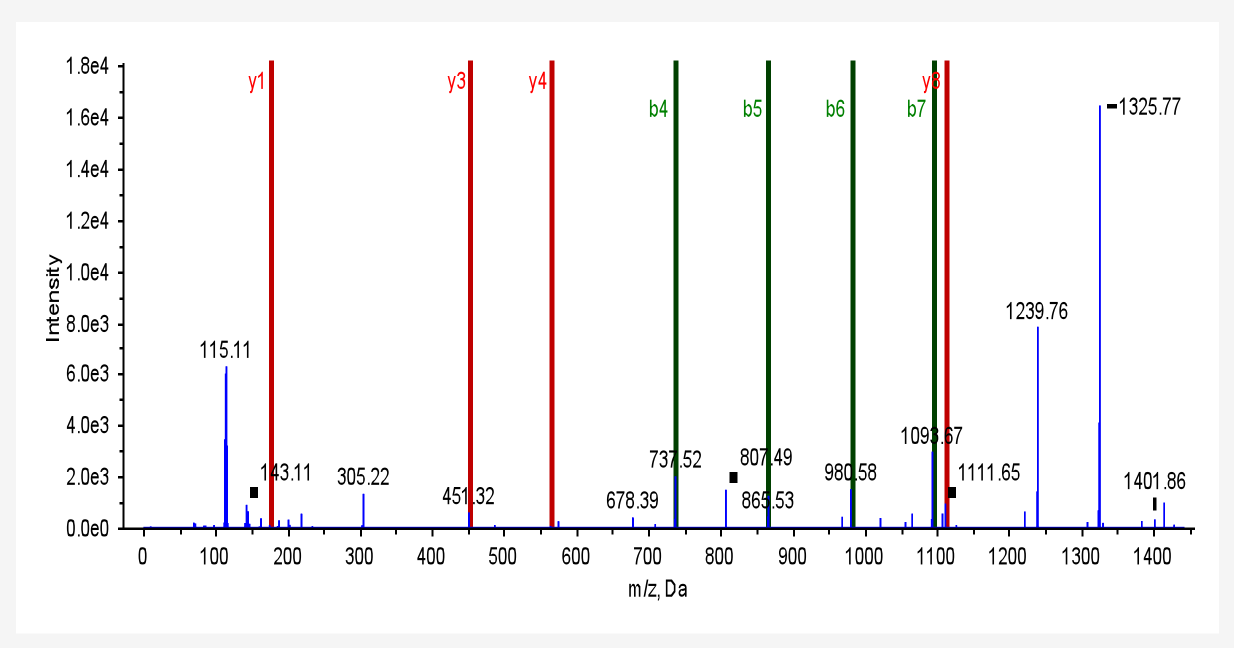

Residue	b	y
G	362.23	1543.85
A	433.27	1182.63
F	580.34	1111.59
R	736.44	964.52
Q[Dea]	865.48	808.42
D	980.51	679.38
L	1093.59	564.35
L	1206.68	451.27
Y	1369.74	338.18
R	1525.84	175.12

Competitor Protein: DVU0818_HAGTPTAAAR

m/z 1256.683 iTRAQ8plex@0; confidence 0.9756

Primary ID: DVU1419_HSWPGNVR

1F5_C12_S5_213_20090306_(2_3_6).group

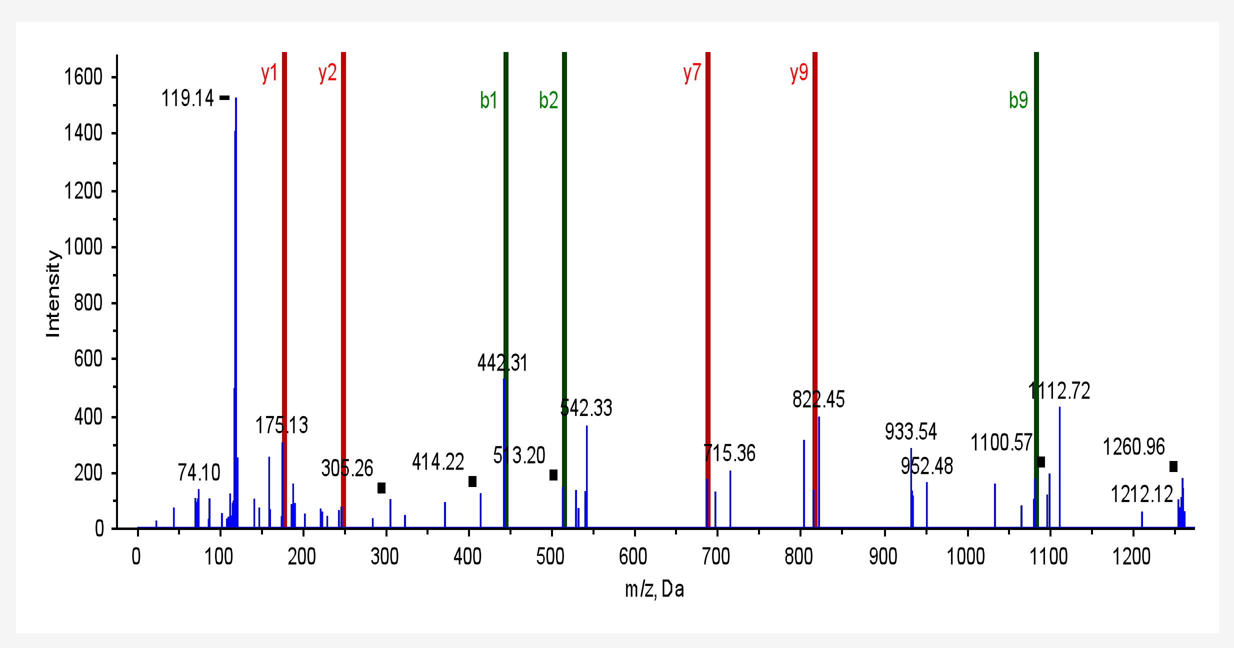

Residue	b	y
H	442.27	1256.70
A	513.31	815.44
G	570.33	744.40
T	671.38	687.38
P	768.43	586.33
T	869.48	489.28
A	940.52	388.23
A	1011.55	317.19
A	1082.59	246.16
R	1238.69	175.12

Competitor Protein: DVU0888_IANALK

m/z 1237.823 iTRAQ8plex@6;iTRAQ8plex@0; cleaved L-I@N-term

confidence 0.9716 Primary ID: DVU2250A_LANALK

AS2_613-615_05292012_(2_3_2).group

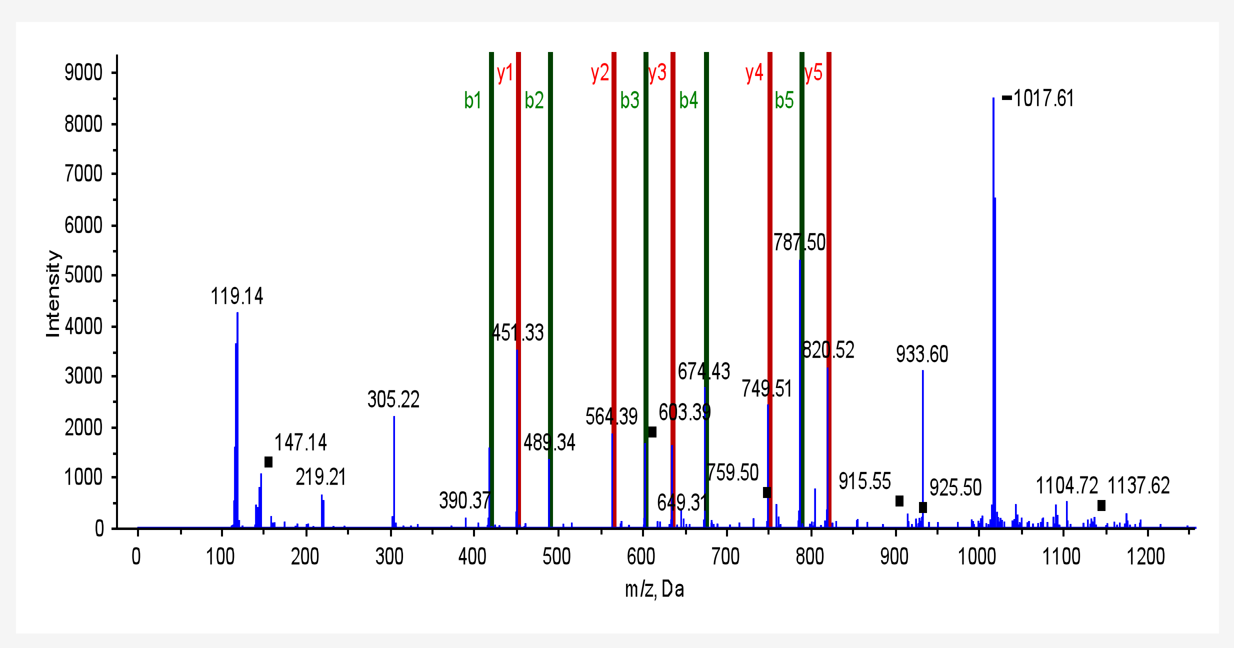

Residue	b	y
I	418.30	1237.81
A	489.33	820.52
N	603.38	749.48
A	674.41	635.44
L	787.50	564.40
K[IT8]	1219.80	451.32

Competitor Protein: DVU0947_SRILL

m/z 905.6853 iTRAQ8plex@0; cleaved L-S@N-term; cleaved L-G@C-term; missed R-I@2

confidence 0.99

Primary ID: DVU2336_AGDLIL

1E6_2D_SEC_B13-C6_08242010_(3_4_4)_(3_4_3).group

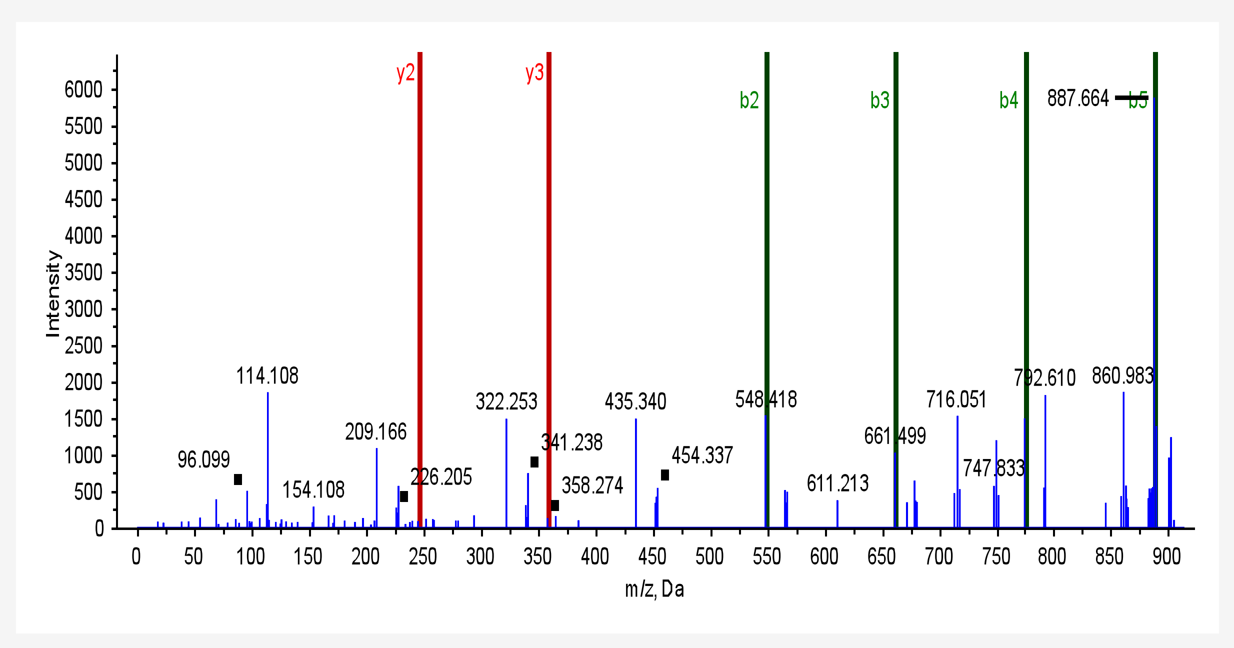

Residue	b	y
S	392.2447	905.6085
R	548.3458	514.3711
I	661.4298	358.27
L	774.5139	245.186
L	887.598	132.1019

Multiple Competitor Proteins for LLPDR

m/z 917.6387 iTRAQ8plex@0; semitryptic confidence 0.9601

DVU0998, DVU2396, DVU2501, DVU3267

Primary ID: DVU3294_LIVLR

S5_405-407_04232010_(2_3_6).group

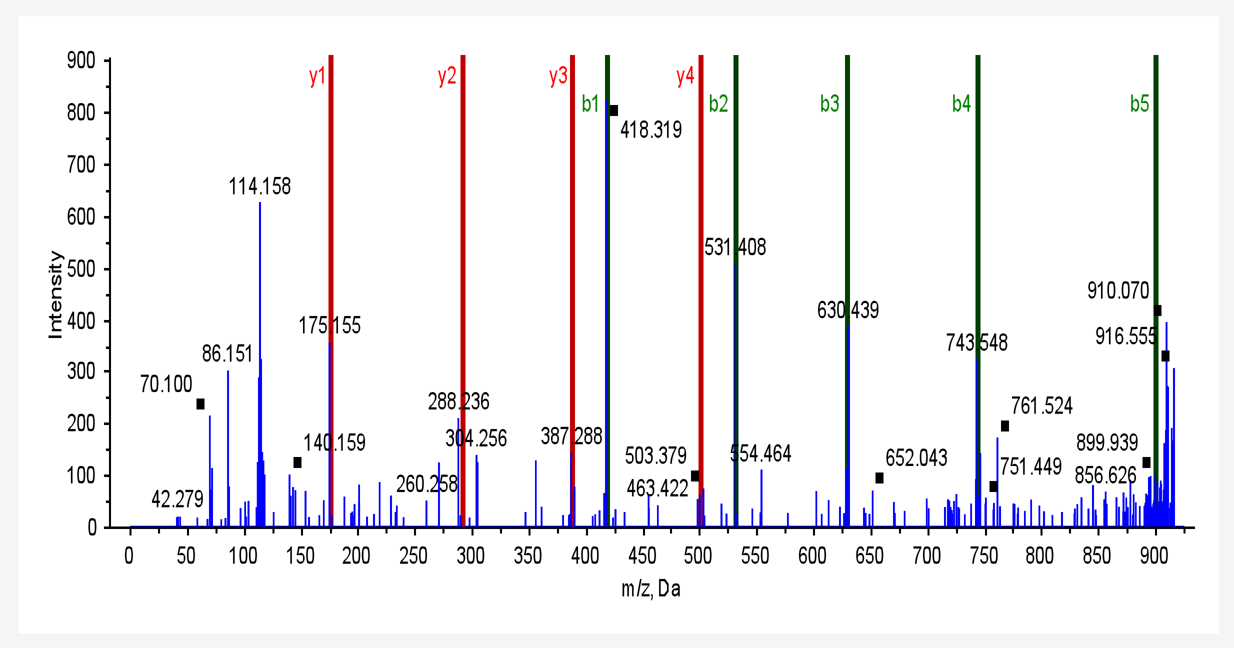

Residue	b	y
L	418.30	917.57
L	531.38	500.28
P	628.43	387.20
D	743.46	290.15
R	899.56	175.12

Competitor Protein: DVU1019_YFALK

m/z 1249.743 iTRAQ8plex@5;iTRAQ8plex@0; confidence 0.9717

Primary ID: DVU3025_YSMLK

2D1_2D_tagless_SEC_B13-C6_05-14-2010_(3_4_2).group

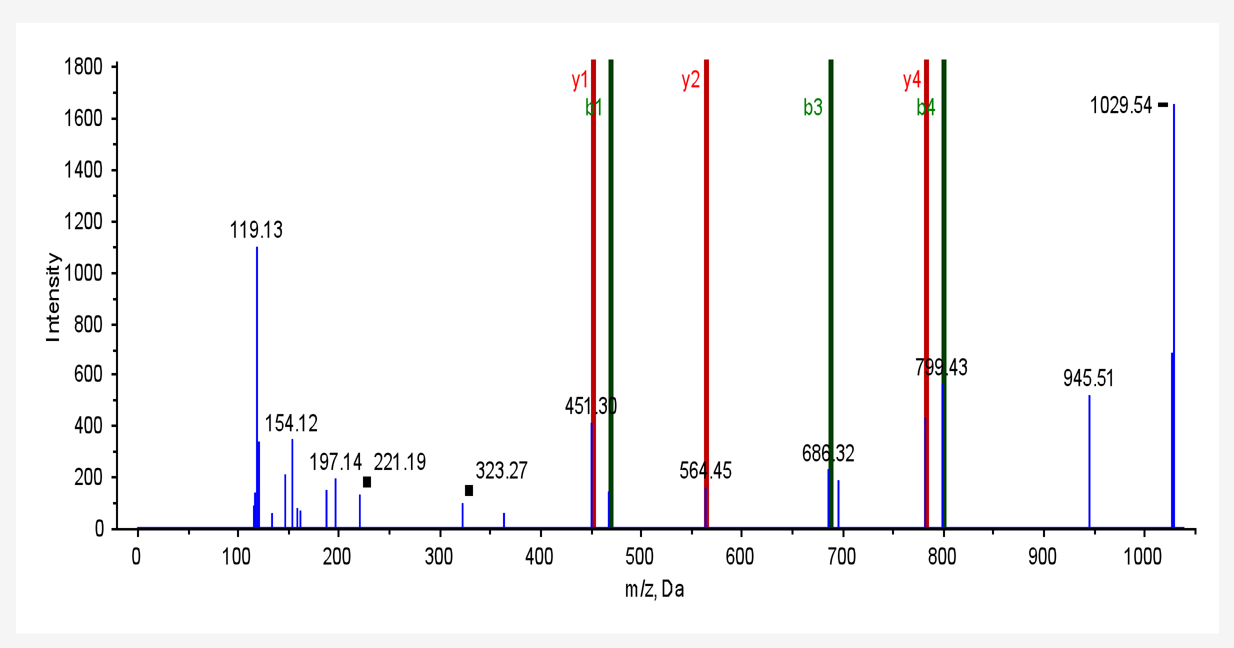

Residue	b	y
Y	468.28	1249.78
F	615.34	782.51
A	686.38	635.44
L	799.47	564.40
K[IT8]	1231.77	451.32

Competitor Protein: DVU1065_TLGLK

m/z 1139.755 iTRAQ8plex@5;iTRAQ8plex@0; cleaved A-T@N-term; cleaved K-P@C-term

confidence 0.9574

Primary ID: DVU1950_TLGLK

SEC_B11B13_&_C4C6_HIC_combos_B13-D7_091109_(2_3_4).group

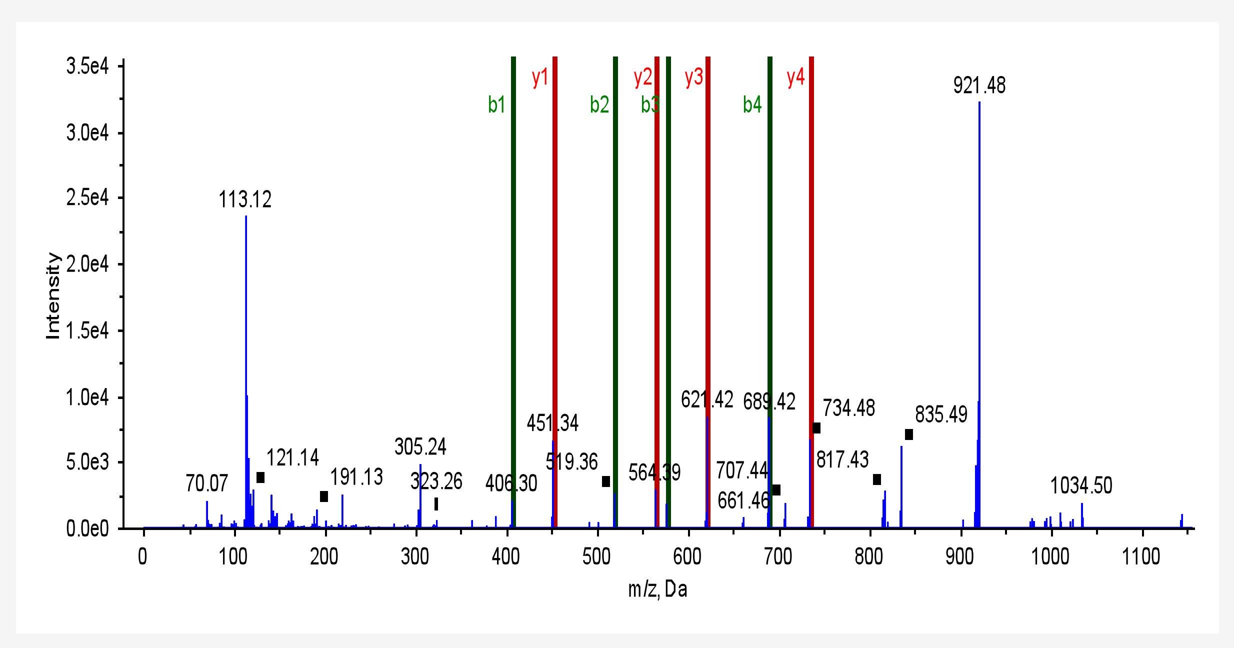

Residue	b	y
T	406.26	1139.76
L	519.34	734.51
G	576.37	621.42
L	689.45	564.40
K[IT8]	1121.75	451.32

DVU1290_NTSMEIQNGPR

m/z 1566.684 Oxidation@4;iTRAQ8plex@0; confidence 0.99

2B6_2D_SEC_B12-C5_03082011_(2_3_10).group

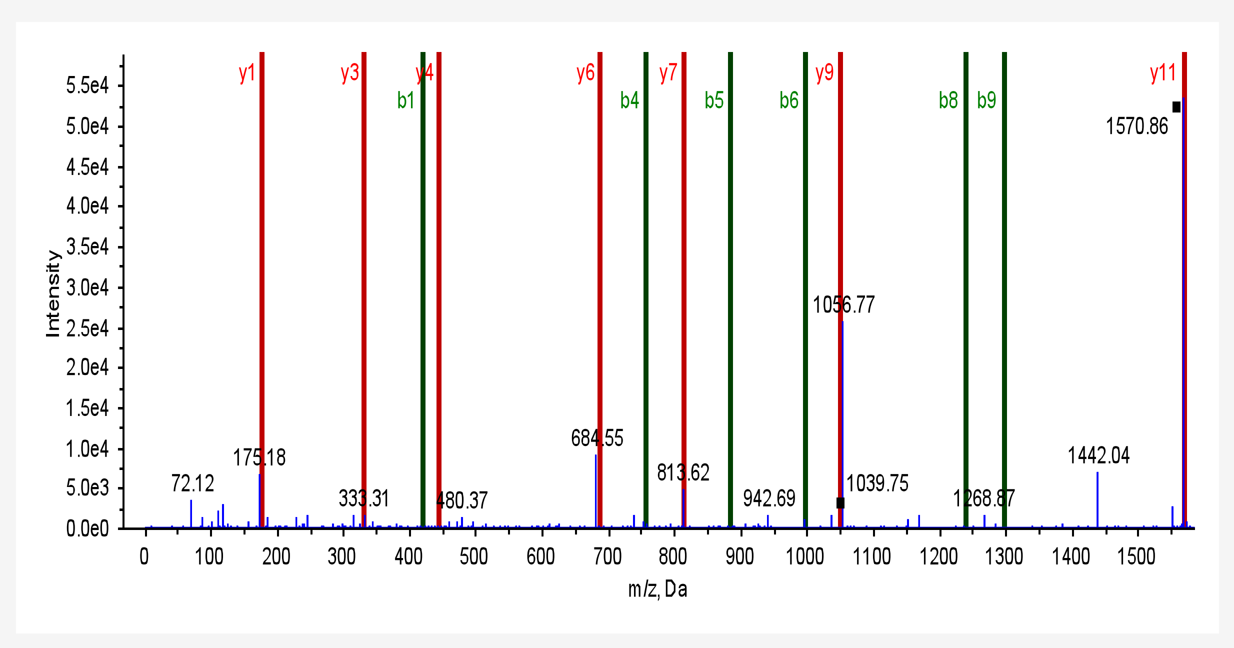

Residue	b	y
N	419.26	1566.78
T	520.30	1148.54
S	607.34	1047.49
M[Oxi]	754.37	960.46
E	883.41	813.42
I	996.50	684.38
Q	1124.56	571.29
N	1238.60	443.24
G	1295.62	329.19
P	1392.67	272.17
R	1548.77	175.12

Multiple IDs (confidence 0.99):

DVU1012_IDIK 1096.979 iTRAQ8plex@4;iTRAQ8plex@0; cleaved N-I@N-term

DVU2812_IDIK 1096.979 iTRAQ8plex@4;iTRAQ8plex@0; cleaved W-I@N-term

S5_256_258_261_265_selected_4plexes_relabeled_092509_(2_3_1).group

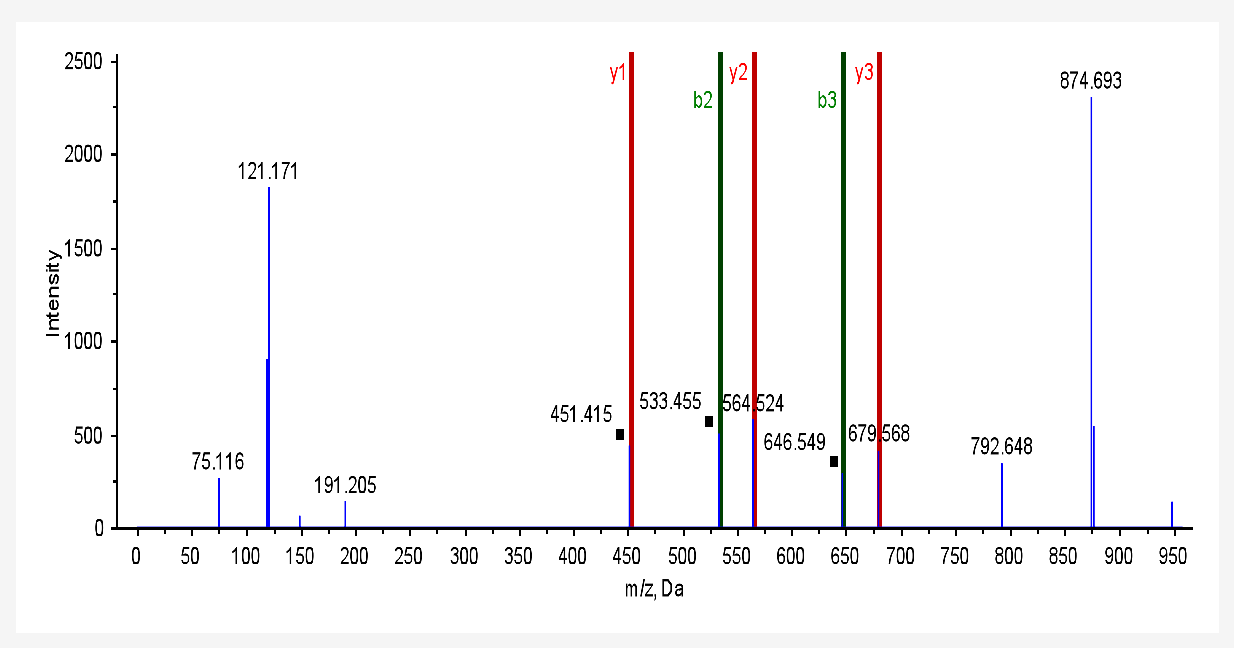

Residue	b	y
I	418.30	1096.72
D	533.32	679.43
I	646.41	564.40
K[IT8]	1078.71	451.32

Multiple IDs for SRLAERLDGPHR: DVU1398, DVU2030, DVU2178

m/z 1711.001 iTRAQ8plex@0; missed R-L@2; missed R-L@6 confidence 0.99

AS1_1D2_633_2D_SEC_C5_06182012_(3_4_2).group

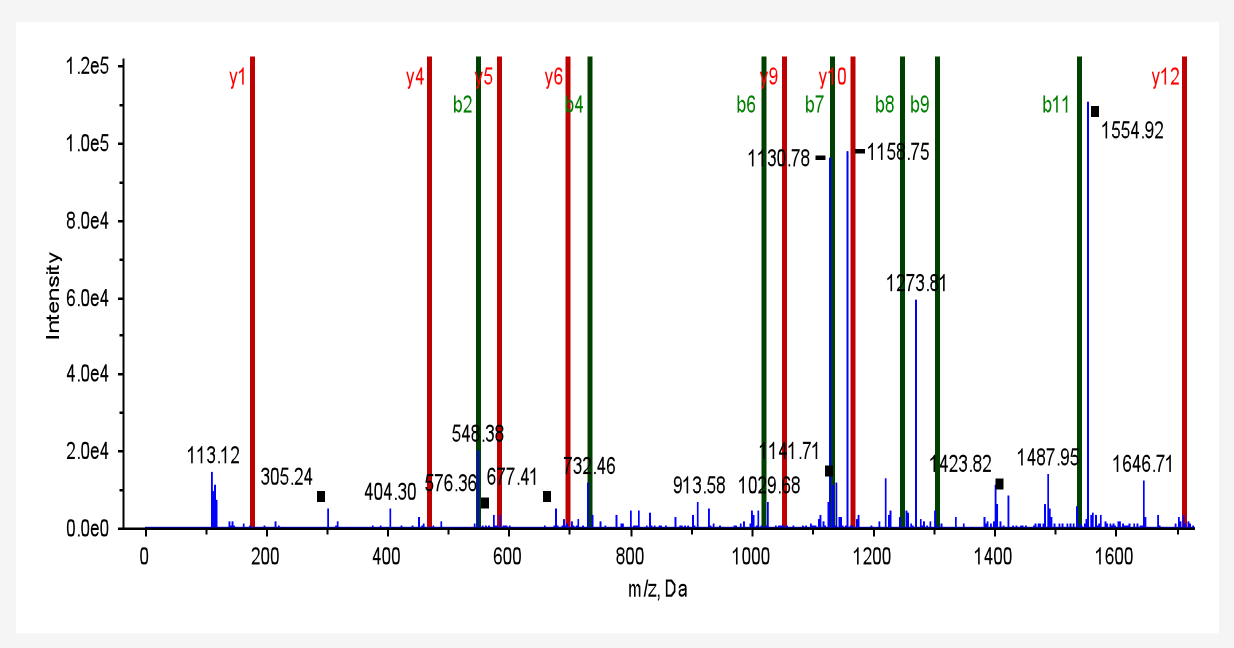

Residue	b	y
S	392.24	1710.97
R	548.35	1319.73
L	661.43	1163.63
A	732.47	1050.54
E	861.51	979.51
R	1017.61	850.46
L	1130.69	694.36
D	1245.72	581.28
G	1302.74	466.25
P	1399.8	409.23
H	1536.85	312.18
R	1692.96	175.12

Competitor Protein: DVU1435_AGAGGIIK

m/z 1294.85 iTRAQ8plex@8;iTRAQ8plex@0; confidence 0.99

Primary ID: DVU0881_AEILLK

2D4_C5_S5_291_relabeled_092509_(2_3_11).group

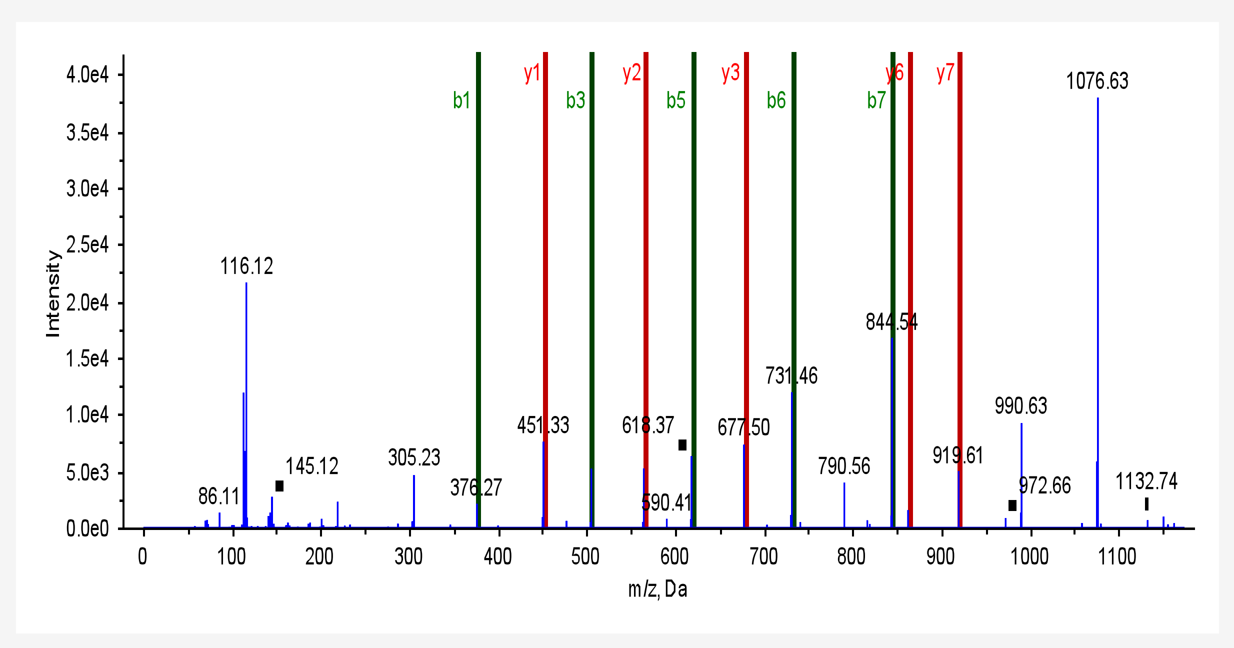

Residue	b	y
A	376.25	1294.83
G	433.27	919.59
A	504.31	862.57
G	561.33	791.53
G	618.35	734.51
I	731.44	677.49
I	844.52	564.40
K[IT8]	1276.82	451.32

Competitor Protein: DVU1516_DARALK

m/z 1281.779 iTRAQ8plex@6;iTRAQ8plex@0; cleaved T-D@N-term; missed R-A@3 confidence 0.9578

Primary ID: DVU1976_NPMAIK

2B6_D5_D7_S5_289_290_06162009_(2_3_4).group

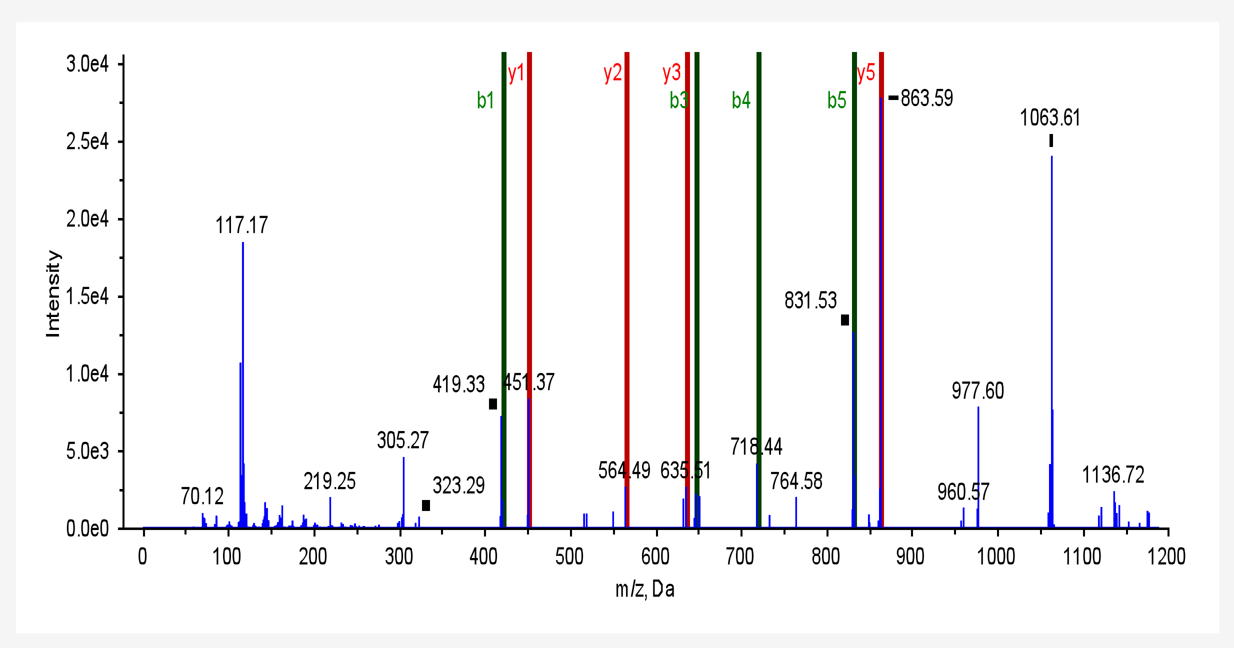

Residue	b	y
D	420.24	1281.81
A	491.28	862.58
R	647.38	791.54
A	718.41	635.44
L	831.50	564.40
K[IT8]	1263.80	451.32

Competitor Protein: DVU1553_ALLAER

m/z 976.6179 iTRAQ8plex@0; cleaved D-A@N-term confidence 0.9532

Primary ID: DVU1419_ALILSR

1F5_C8_C10_S5_211_212_20090219_(3_4_6)_(2_3_6).group

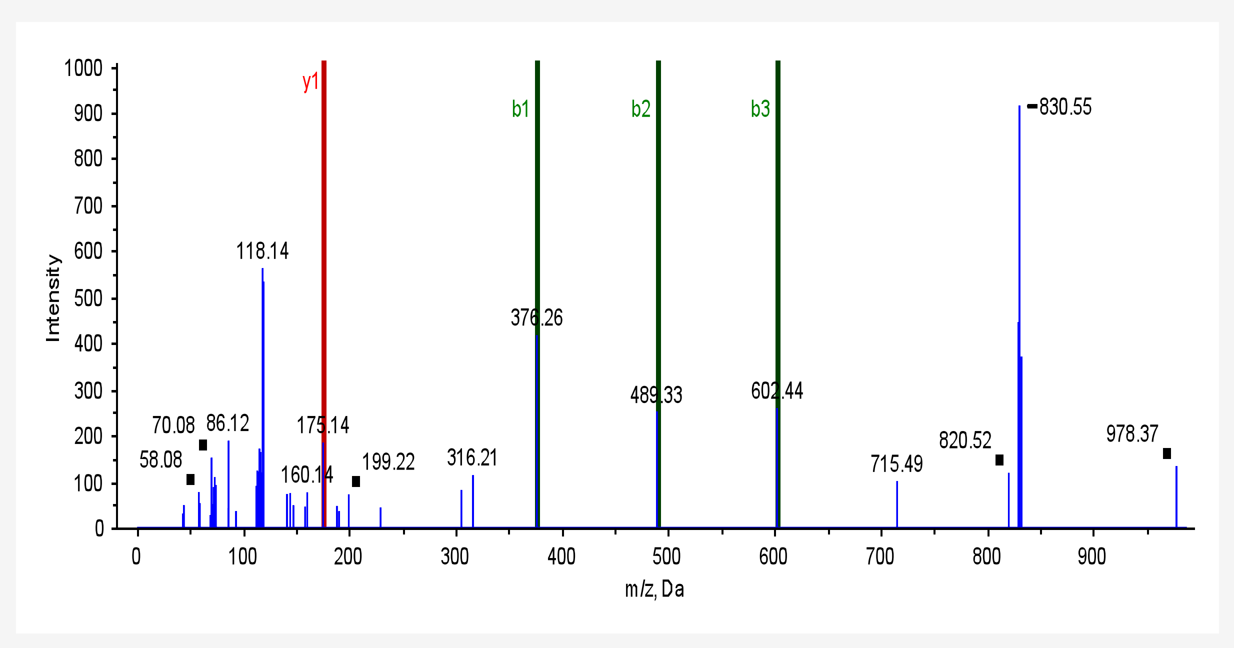

Residue	b	y
A	376.25	976.61
L	489.33	601.37
L	602.42	488.28
A	673.46	375.20
E	802.50	304.16
R	958.60	175.12

DVU1641_VDTMSRHIDLR

m/z 1646.913 iTRAQ8plex@0; missed R-H@6 confidence 0.99

2E6_2D_SEC_B11_#2_B13-C4_14Feb2011_(2_3_8).group

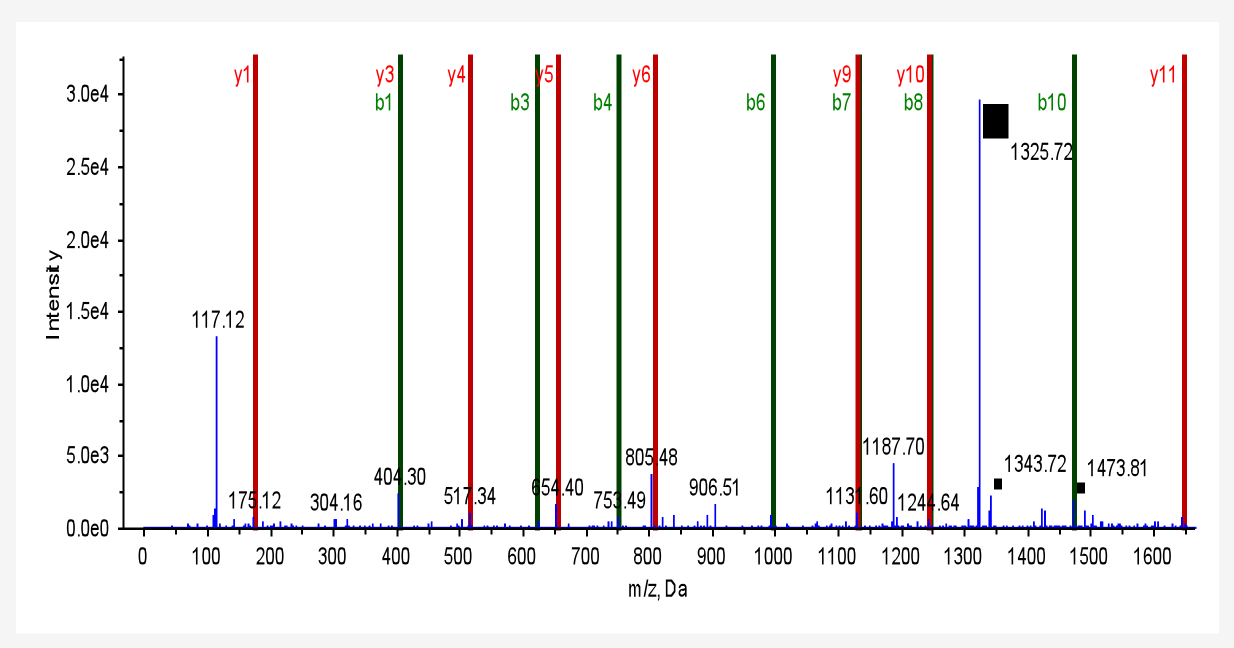

Residue	b	y
V	404.28	1646.9
D	519.31	1243.62
T	620.36	1128.59
M	751.4	1027.55
S	838.43	896.51
R	994.53	809.47
H	1131.59	653.37
I	1244.67	516.31
D	1359.7	403.23
L	1472.78	288.2
R	1628.88	175.12

Competitor Protein: DVU1672_DGEYLAR

m/z 1127.598 iTRAQ8plex@0; confidence 0.99

Primary ID: DVU0894A_DYGEIAR

2E3_C10_C12_S5_312_313_rerun_080509_(3_23_7)_(3_24_7)_(3_25_7)_(3_26_7)_(3_27_7)_(3_28_7).group

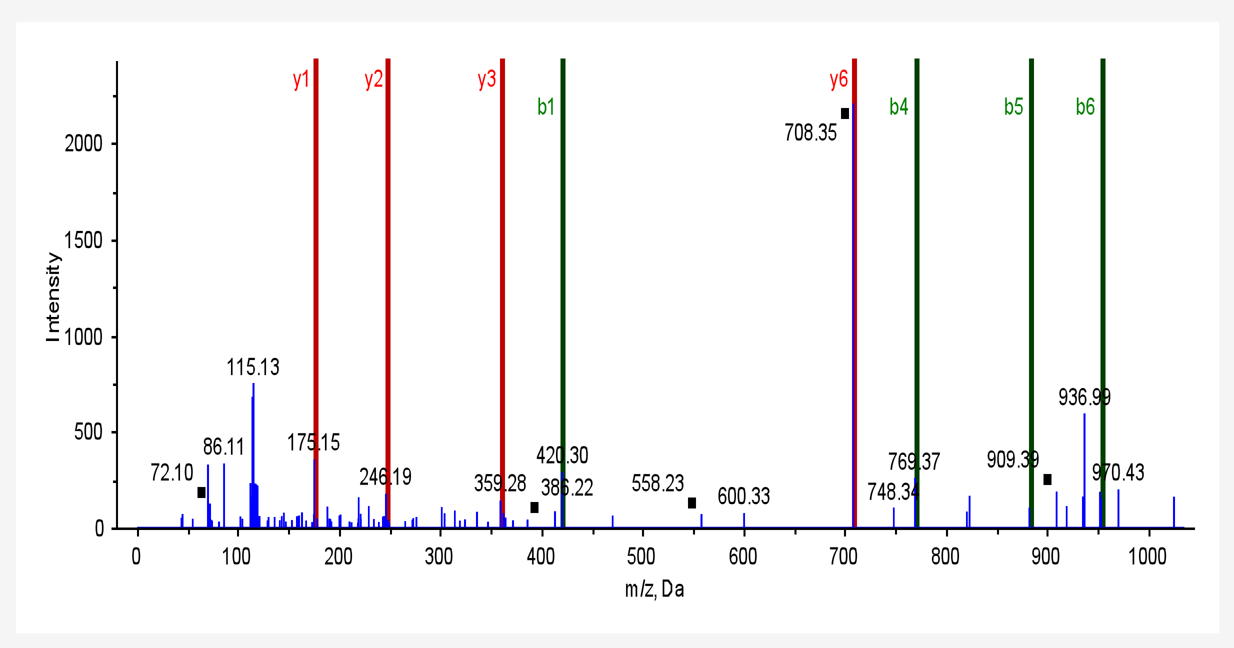

Residue	b	y
D	420.24	1127.6
G	477.26	708.37
E	606.3	651.35
Y	769.37	522.3
L	882.45	359.24
A	953.49	246.16
R	1109.59	175.12

Competitor Protein: DVU1758_VTIAGK

m/z 1196.771 iTRAQ8plex@6;iTRAQ8plex@0; confidence 0.99

Primary ID: DVU1623_VTIQK

MonoQ_2A3_S5_142_2D_SEC_B11-C2_120409_(2_3_6).group

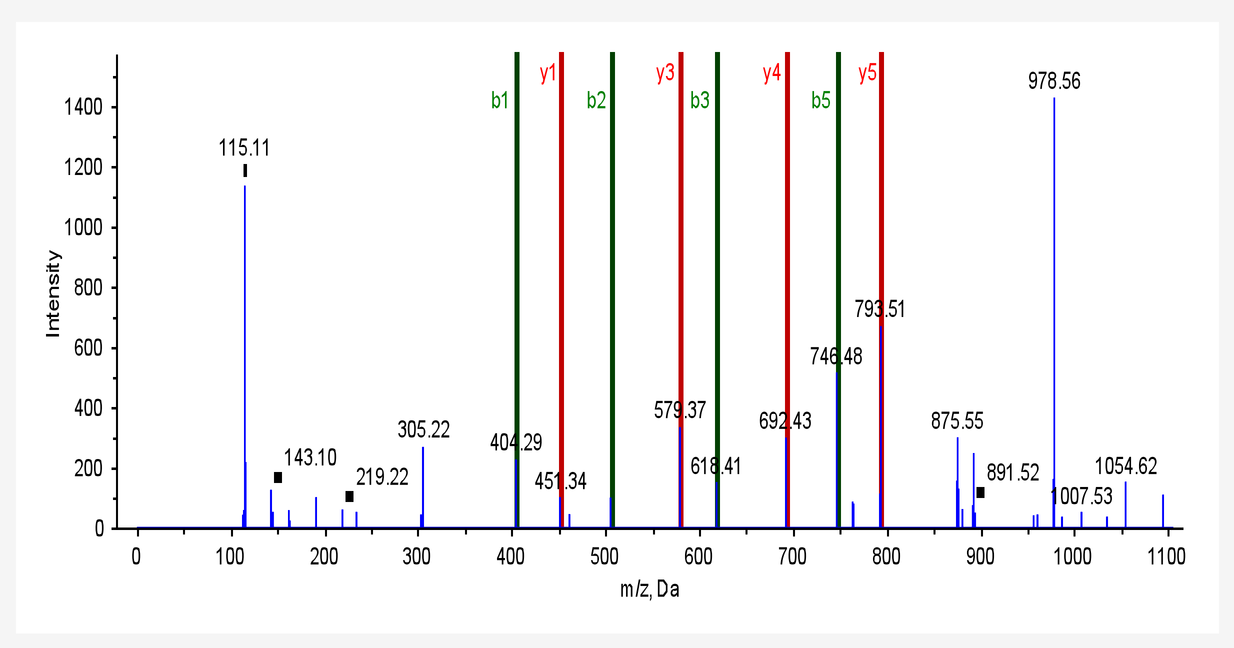

Residue	b	y
V	404.28	1196.78
T	505.33	793.51
I	618.41	692.46
A	689.45	579.38
G	746.47	508.34
K[IT8]	1178.77	451.32

Multiple IDs (confidence 0.99)
DVU0636_IAALLR m/z 960.6447 iTRAQ8plex@0; cleaved H-I@N-term
DVU0651_LAALLR m/z 960.6447 iTRAQ8plex@0; cleaved G-L@N-term
DVU0896_LAAILR m/z 960.6447iTRAQ8plex@0; cleaved S-L@N-term **DVU1772_LAALLR** m/z 960.6447 iTRAQ8plex@0; cleaved S-L@N-term
S5_143_2D_tagless_SEC_B13-C6_122109_(2_3_6).group

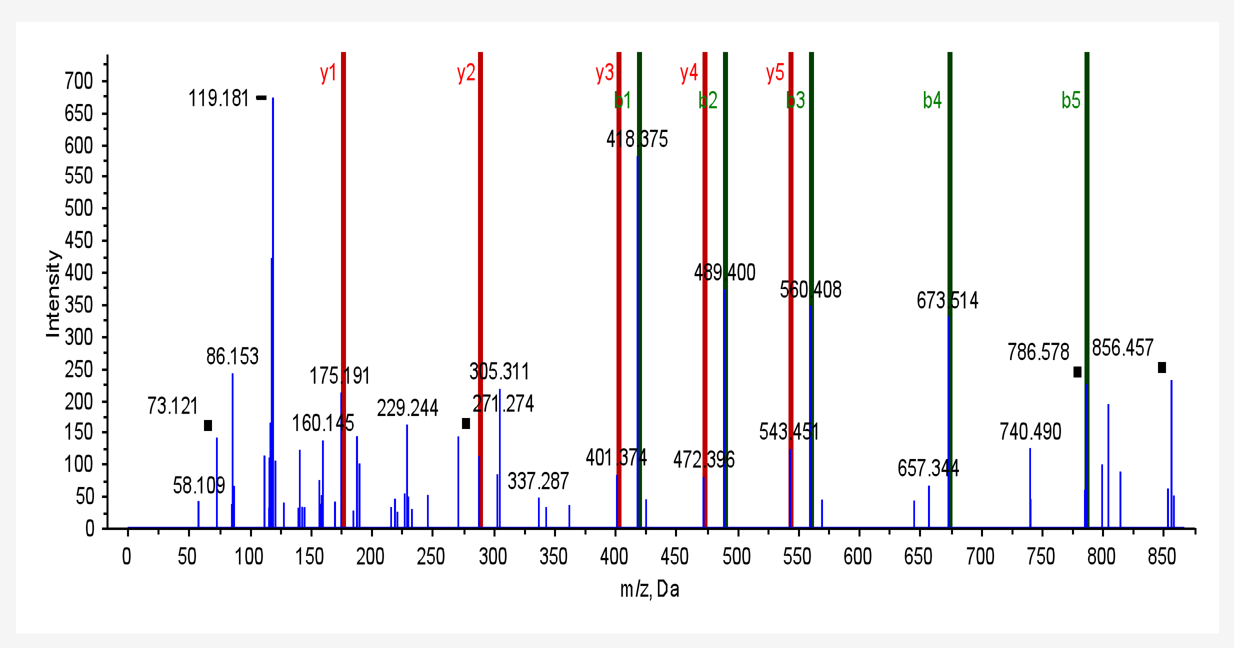

Residue	b	y
L/I	418.30	960.65
A	489.33	543.36
A	560.37	472.32
L/I	673.46	401.29
L/I	786.54	288.20
R	942.64	175.12

Multiple IDs for LLFLR:

m/z 965.642 iTRAQ8plex@0; semitryptic confidence 0.99

DVU1884, DVU2570, DVU2684, DVU3236

S5_405-407_04232010_(2_3_9).group

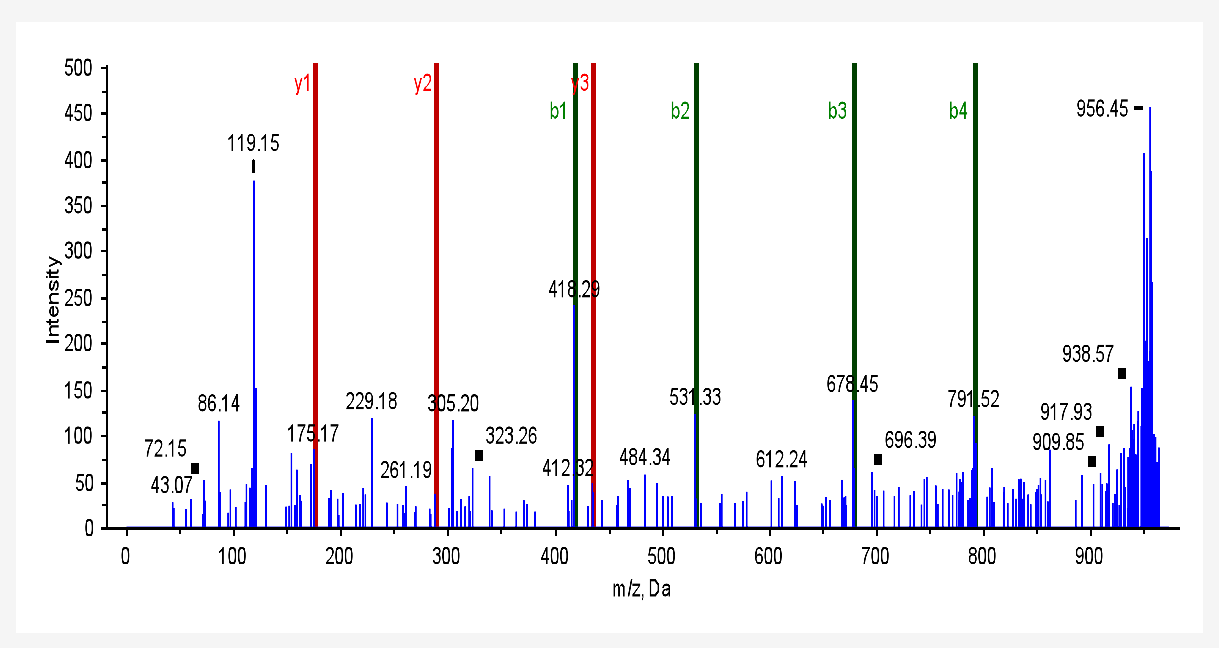

Residue	b	y
L	418.30	965.64
L	531.38	548.36
F	678.45	435.27
L	791.53	288.20
R	947.63	175.12

Competitor Protein: DVU1962_MVASATVLADSAR

m/z 1611.995 Oxidation@1;iTRAQ8plex@0; confidence 0.99

Primary ID: P01267_FVAPESLK

2D4_C5_S5_291_relabeled_092509_(2_3_12).group

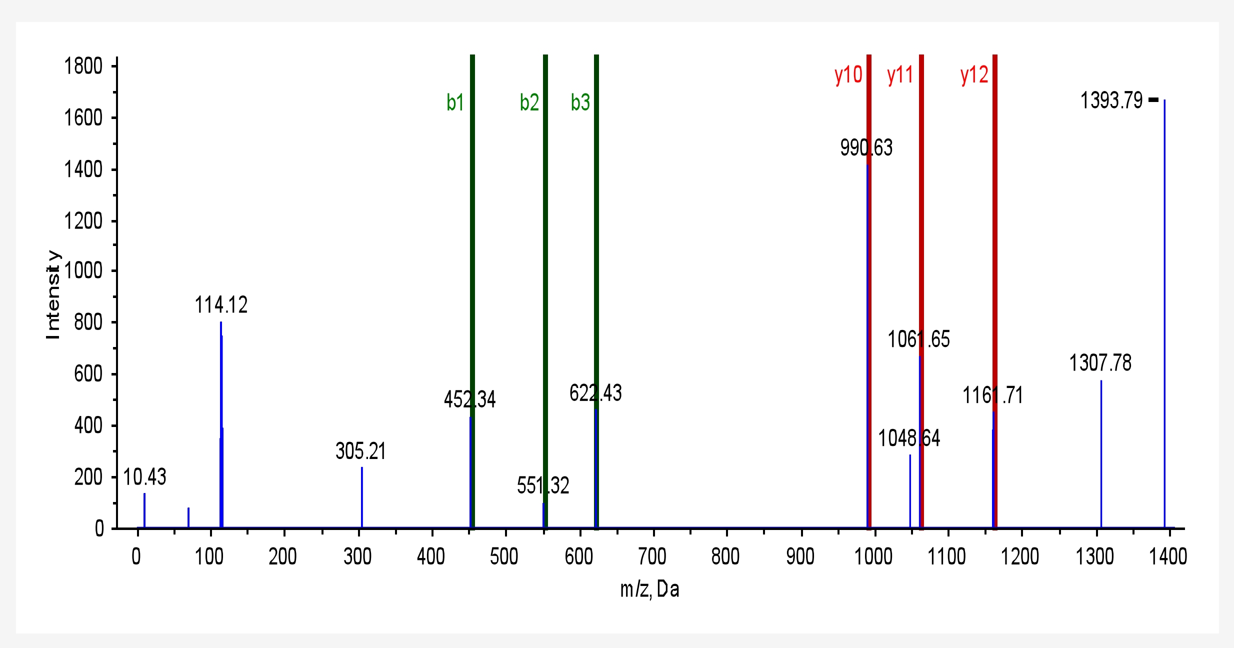

Residue	b	y
M[Oxi]	452.25	1611.87
V	551.32	1160.63
A	622.35	1061.56
S	709.39	990.52
A	780.42	903.49
T	881.47	832.45
V	980.54	731.40
L	1093.62	632.34
A	1164.66	519.25
D	1279.69	448.22
S	1366.72	333.19
A	1437.76	246.16
R	1593.86	175.12

Competitor Protein: DVU1987_KGDGALTLR

m/z 1538.911 iTRAQ8plex@1;iTRAQ8plex@0; missed K-G@1 confidence 0.9657

DVU1336_KGDGPSITR

2A3_C4_C6_S5_219_220_02-20-2009_(2_3_6).group

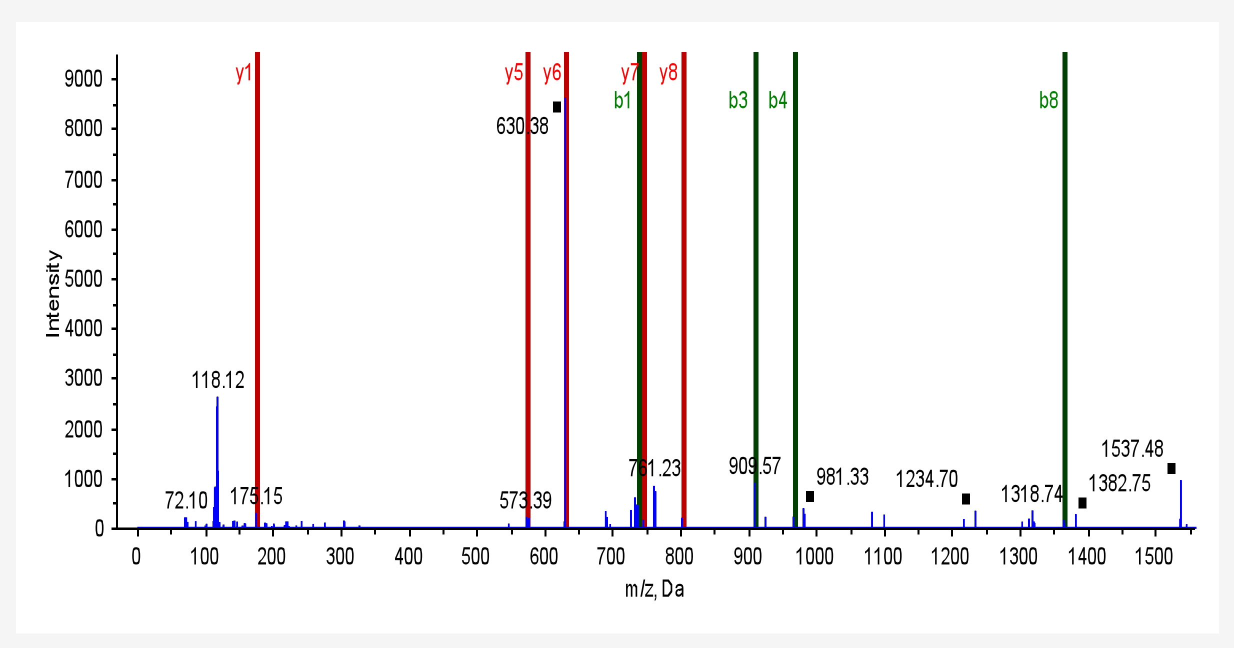

Residue	b	y
K[IT8]	737.51	1538.95
G	794.53	802.44
D	909.56	745.42
G	966.58	630.39
A	1037.62	573.37
L	1150.70	502.33
T	1251.75	389.25
L	1364.84	288.20
R	1520.94	175.12

Competitor Protein: DVU2101_ITGEGLR

m/z 1049.645 iTRAQ8plex@0; confidence 0.99

Primary ID: DVU0398_ITGWIR

2E3_D5_D7_S5_316_317_072409_(2_3_4).group

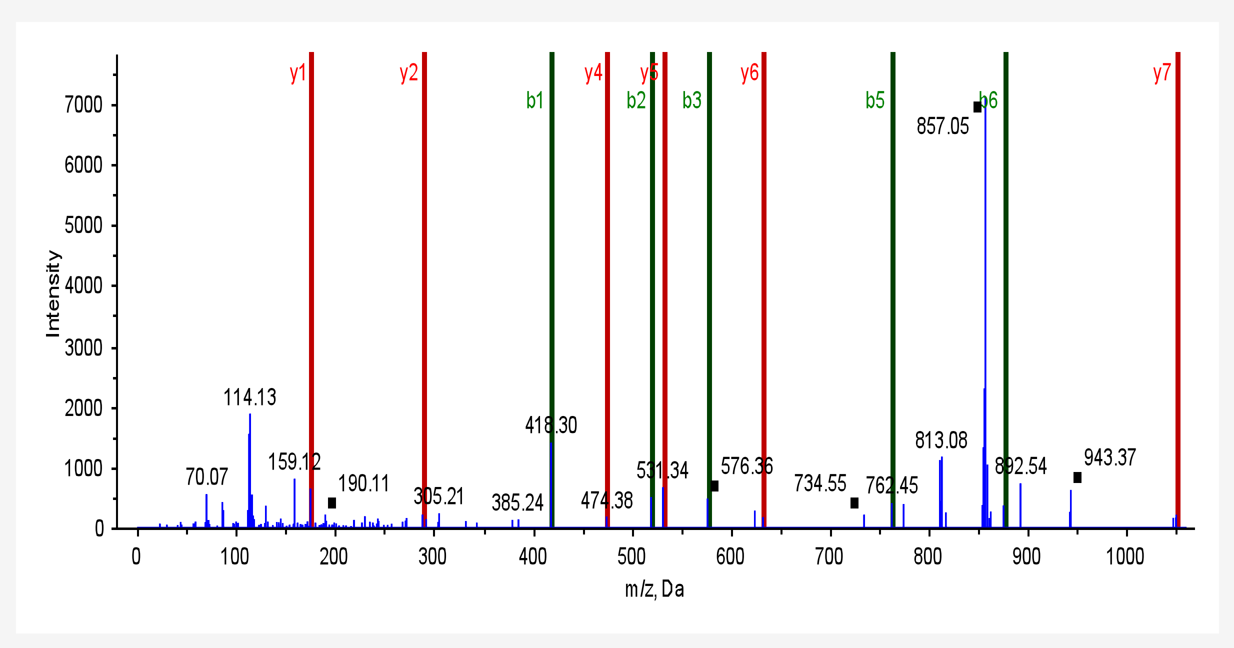

Residue	b	y
I	418.30	1049.63
T	519.34	632.34
G	576.37	531.29
E	705.41	474.27
G	762.43	345.22
L	875.51	288.20
R	1031.62	175.12

Multiple Competitor Proteins:

DVU2148_LAASTYSR **DVU2932_ALAEATHR** **DVUA0047_ALATEAHR**

m/z 1172.729 iTRAQ8plex@0; confidence 0.99

2A6_B14_C2_S5_232_233_rerun_04-01-09_(2_3_2)_(2_3_3).group

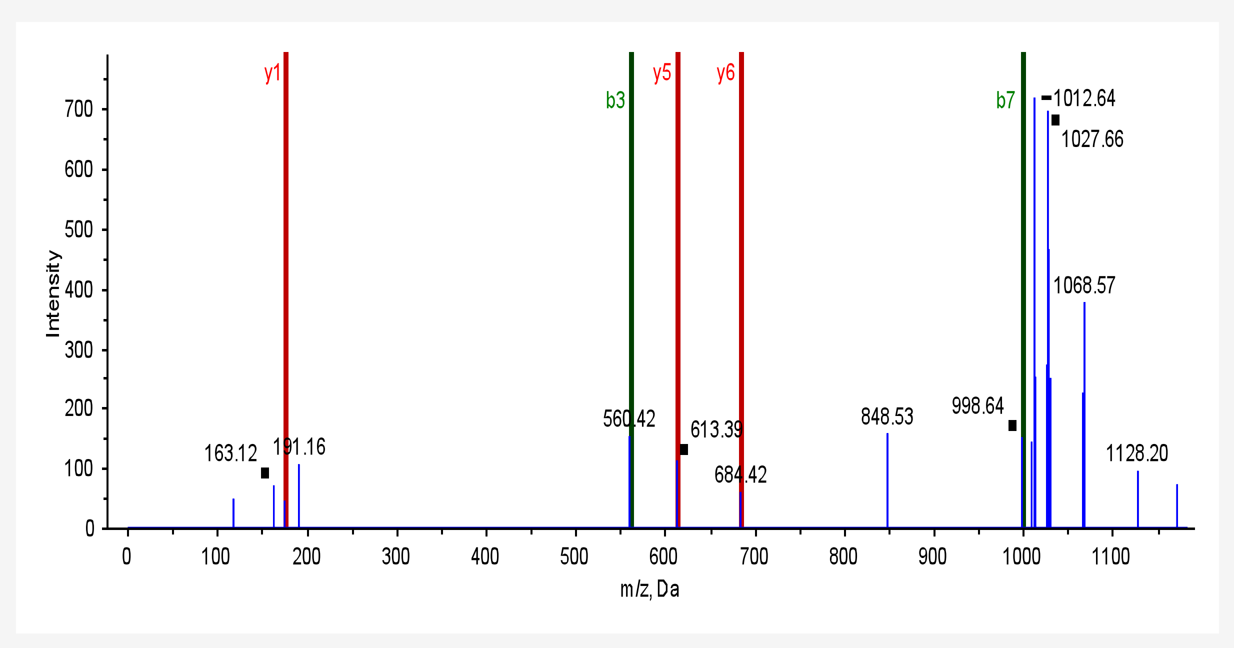

	Residue	b	y
DVU2148	L	418.30	1172.66
	A	489.33	755.37
	A	560.37	684.33
	S	647.40	613.29
	T	748.45	526.26
	Y	911.51	425.21
	S	998.55	262.15
	R	1154.65	175.12

	Residue	b	y
DVUA0047	A	376.25	1172.73
	L	489.33	797.49
	A	560.37	684.40
	P	657.42	613.37
	I	770.51	516.31
	V	869.58	403.23
	E	998.62	304.16
	R	1154.72	175.12

	Residue	b	y
DVU2932	A	376.25	1172.67
	L	489.33	797.43
	A	560.37	684.34
	T	661.42	613.31
	E	790.46	512.26
	A	861.50	383.22
	H	998.56	312.18
	R	1154.66	175.12

DVU2172_KQLQYYEGELER

m/z 2164.097 iTRAQ8plex@1;iTRAQ8plex@0; missed K-Q@1 confidence 0.99

S5_461_#2-3_462_463_102810_(2_3_3)_(2_3_9).group

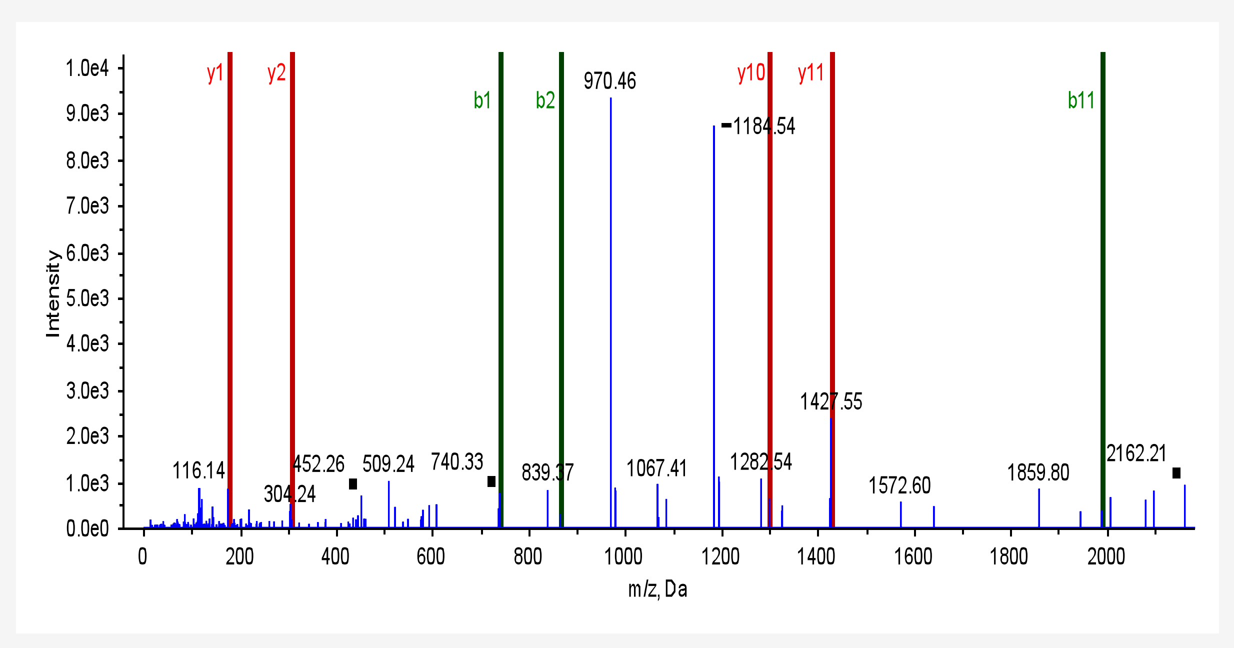

Residue	b	y
K[IT8]	737.51	2164.19
Q	865.57	1427.68
L	978.66	1299.62
Q	1106.71	1186.54
Y	1269.78	1058.48
Y	1432.84	895.42
E	1561.88	732.35
G	1618.90	603.31
E	1747.95	546.29
L	1861.03	417.25
E	1990.07	304.16
R	2146.18	175.12

Competitor Protein: DVU2309_ASGVISQSTDLASR

m/z 1695.943 iTRAQ8plex@0; confidence 0.99

Primary ID: DORF22580_CARWAWMPASR

S5_387_388_389_multiplexes_1-3_03092010_(2_3_3).group

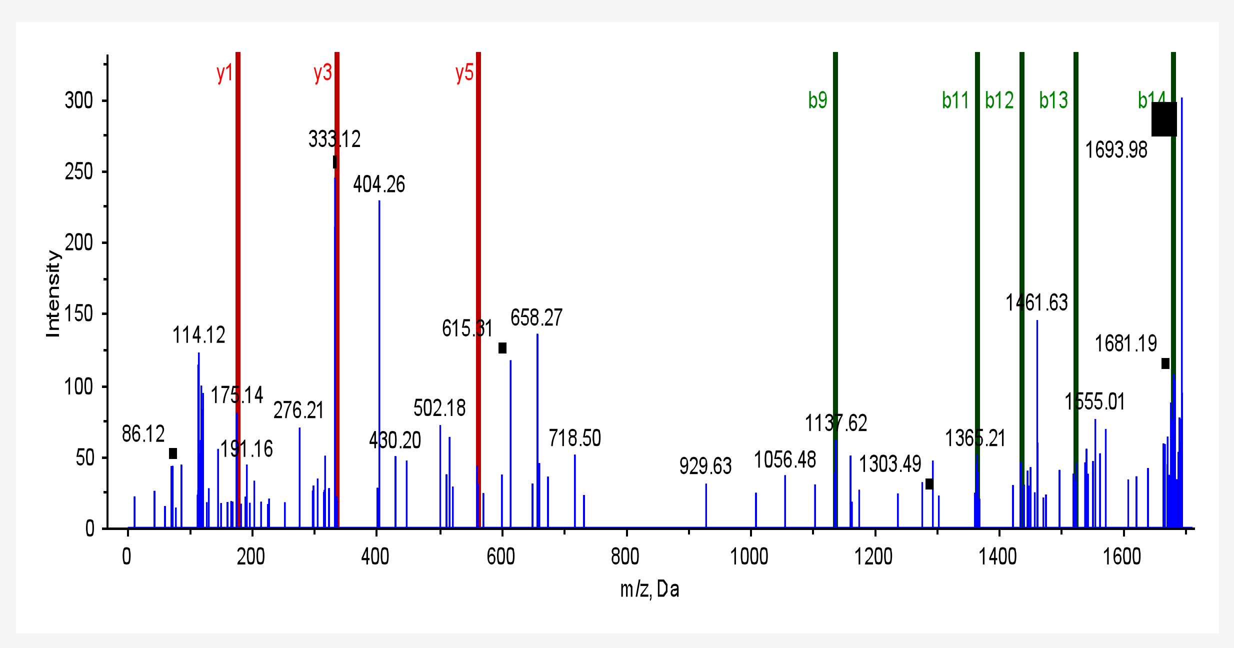

Residue	b	y
A	376.25	1695.92
S	463.28	1320.68
G	520.30	1233.64
V	619.37	1176.62
I	732.46	1077.55
S	819.49	964.47
Q	947.55	877.44
S	1034.58	749.38
T	1135.63	662.35
D	1250.65	561.30
L	1363.74	446.27
A	1434.77	333.19
S	1521.81	262.15
R	1677.91	175.12

Competitor Protein DVU2337_IALLESK

m/z 1381.816 iTRAQ8plex@7;iTRAQ8plex@0; confidence 0.99

Primary ID: DVU3025_IAPEESK

2D1_D5_D7_S5_283_284_plate_A_05-15-09_(2_3_4).group

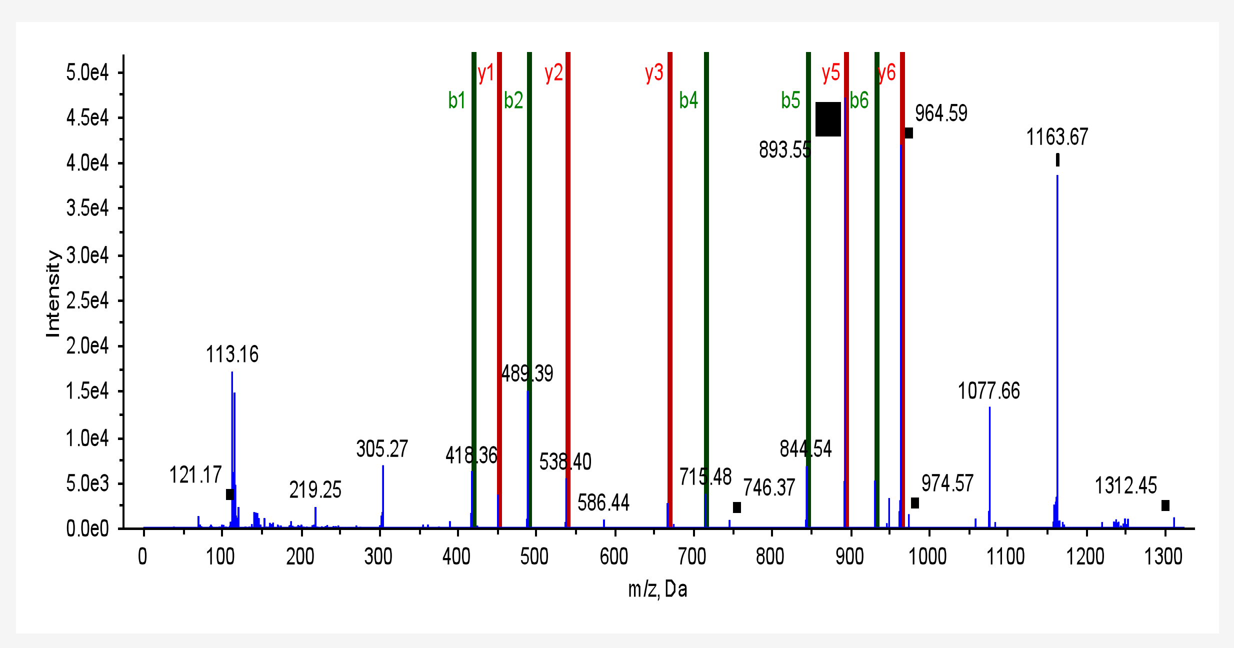

Residue	b	y
I	418.30	1381.89
A	489.33	964.60
L	602.42	893.56
L	715.50	780.48
E	844.54	667.39
S	931.58	538.35
K[IT8]	1363.88	451.32

DVU2460_ALIRIER

m/z 1174.667 iTRAQ8plex@0; missed R-I@4 confidence 0.9646

2D1_C15_D2_S5_280_282_05-29-09_(2_3_4).group

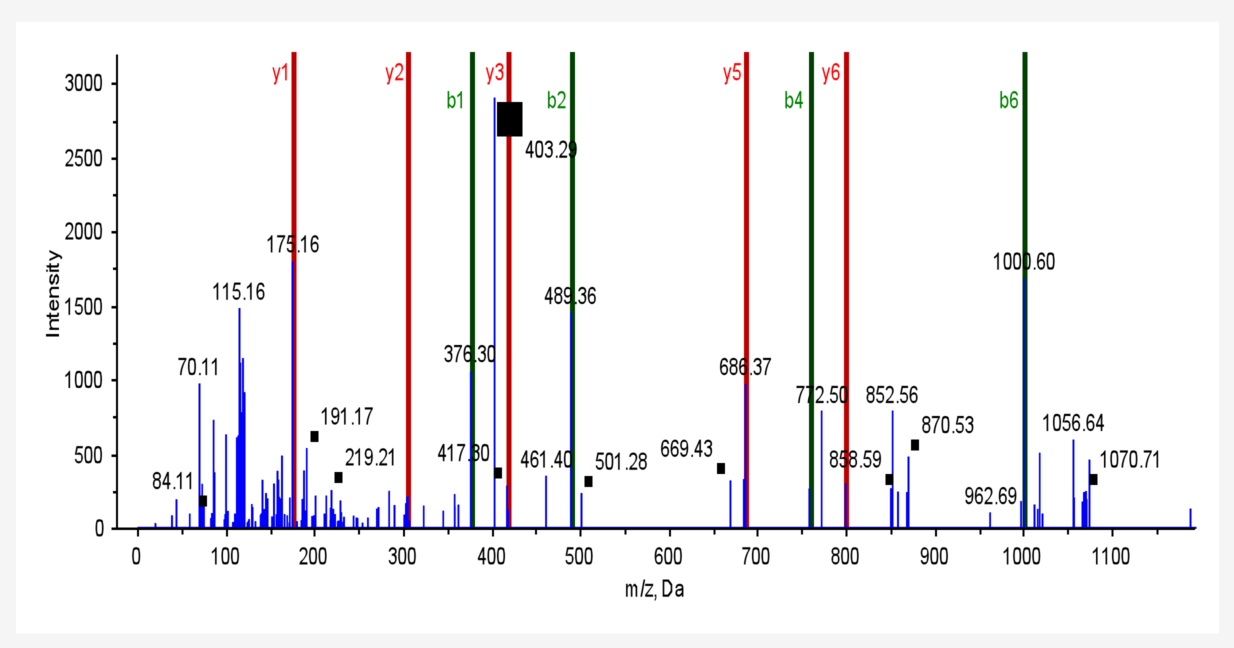

Residue	b	y
A	376.25	1174.76
L	489.33	799.51
I	602.42	686.43
R	758.52	573.35
I	871.60	417.25
E	1000.65	304.16
R	1156.75	175.12

DVU2535_KLWILR

m/z 1436.95 iTRAQ8plex@1;iTRAQ8plex@0; missed K-L@1 confidence 0.9759

2D4_SEC_B7-B15_05072010_(2_3_2).group

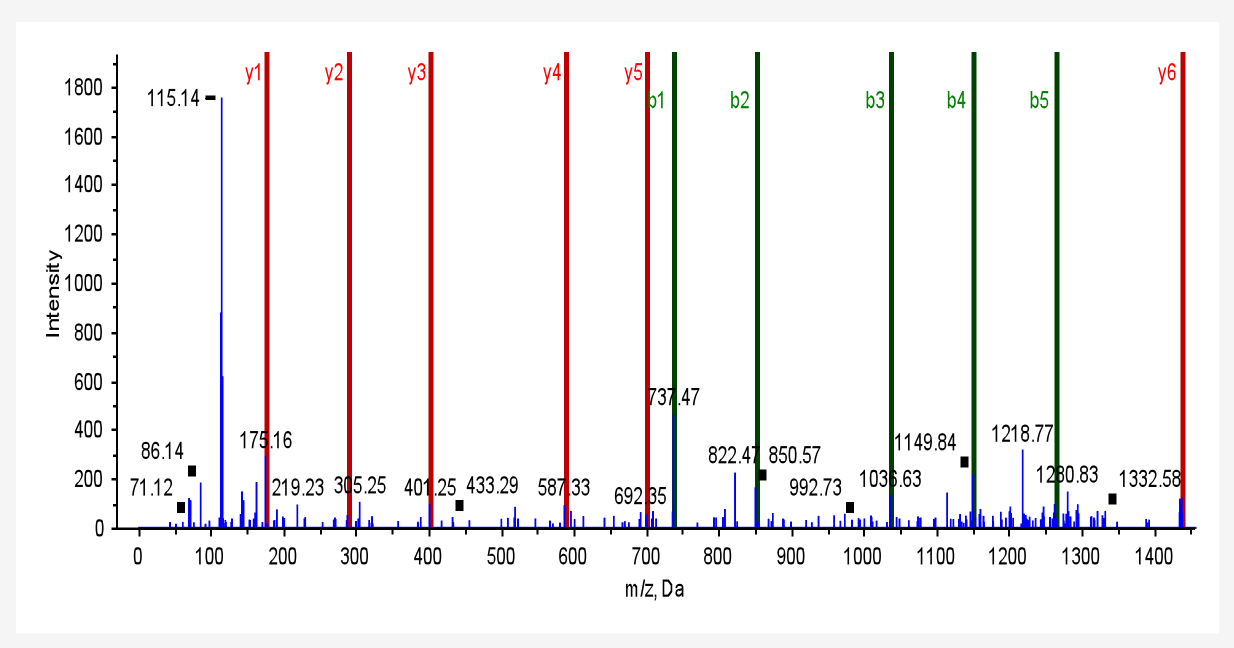

Residue	b	y
K[IT8]	737.51	1436.96
L	850.60	700.45
W	1036.68	587.37
I	1149.76	401.29
L	1262.84	288.20
R	1418.95	175.12

DVU2564_AAWAE

m/z 851.4802 iTRAQ8plex@0; cleaved E-A@N-term; cleaved E-H@C-term

confidence 0.9563

1C7_SEC_B5-B9_07Jan2010_(2_3_6)_(2_3_9).group

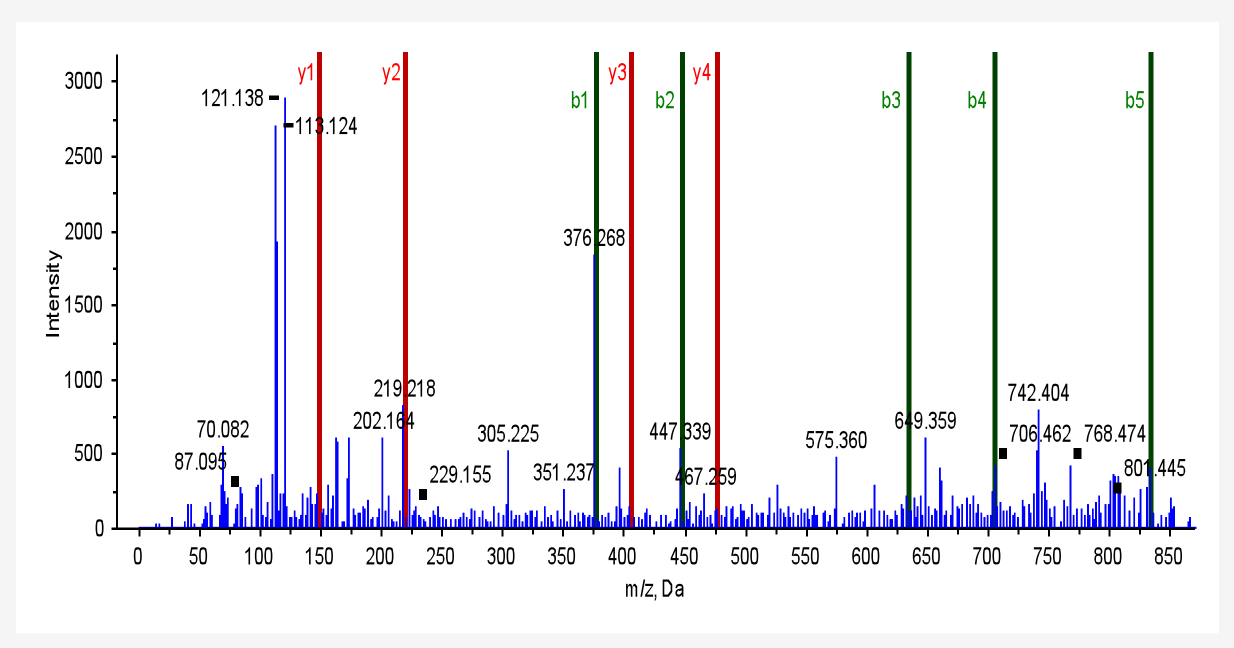

Residue	b	y
A	376.25	851.46
A	447.29	476.21
W	633.37	405.18
A	704.40	219.10
E	833.45	148.06

Competitor Protein: DVU2604_RLYAALAR

m/z 1237.814 iTRAQ8plex@0; missed R-L@1 confidence 0.99

Primary ID: DVU2543_KALAAR

S5_396-398_03-30-2010_(2_3_6).group

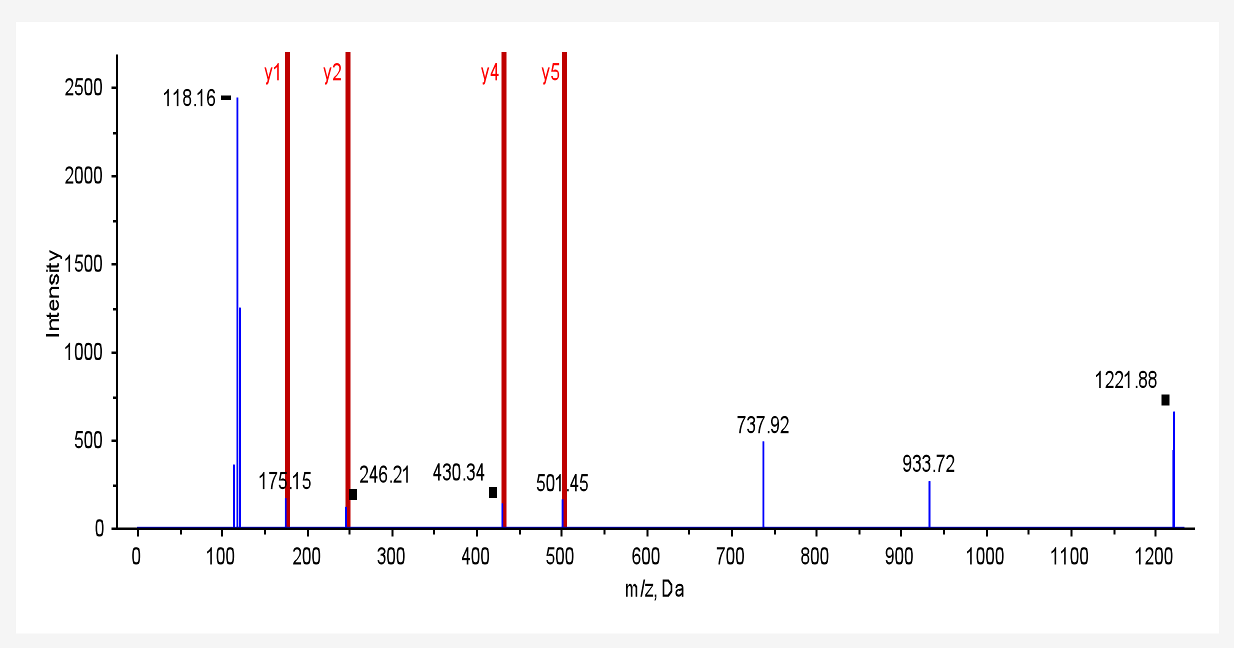

Residue	b	y
R	461.31	1237.77
L	574.40	777.46
Y	737.46	664.38
A	808.50	501.31
A	879.54	430.28
L	992.62	359.24
A	1063.66	246.16
R	1219.76	175.12

Competitor Protein: DVU2637_TLELTR

m/z 876.5247 iTRAQ4plex@0; confidence 0.99

Primary ID: DVU0606_MLELAR

D2_Aldolase_1st_Exp_10-23-07_(2_3_7)_(11_12_8).group

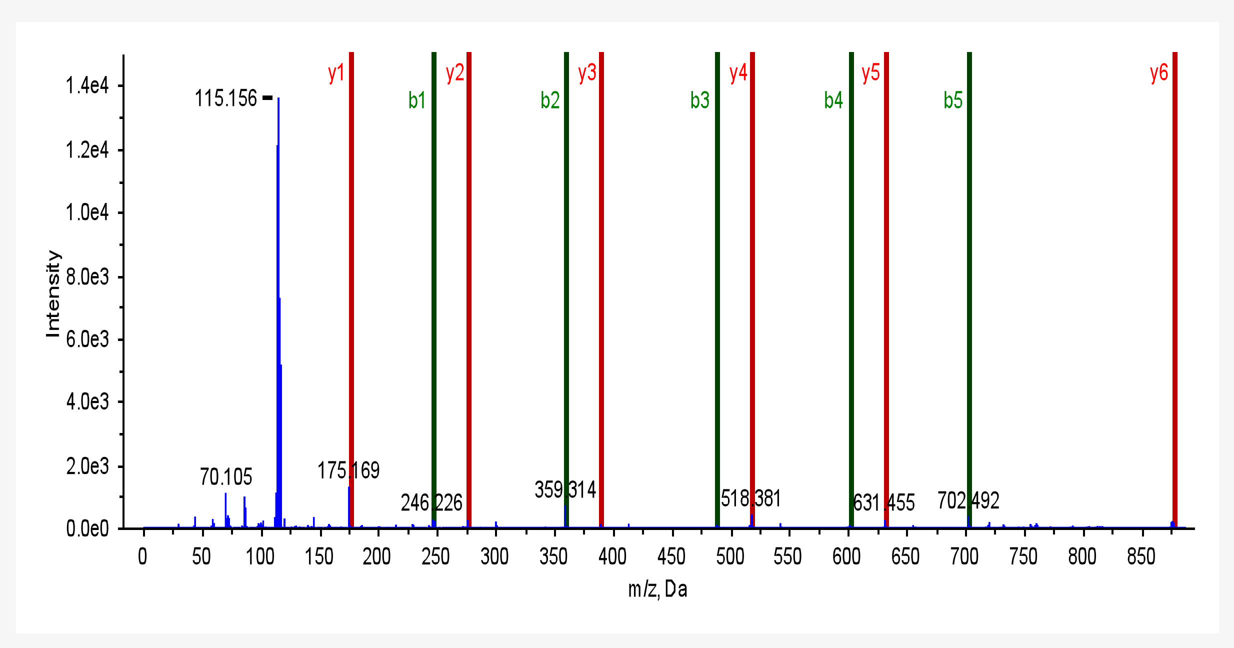

Residue	b	y
T	246.157	876.5271
L	359.2411	631.3774
E	488.2837	518.2933
L	601.3677	389.2507
T	702.4154	276.1666
R	858.5165	175.119

DVU2647_YFATPFPAR

m/z 1373.74 iTRAQ8plex@0; cleaved Q-Y@N-term confidence 0.99

S5_390_391_multiplexes_1-3_03122010_(2_3_6).group

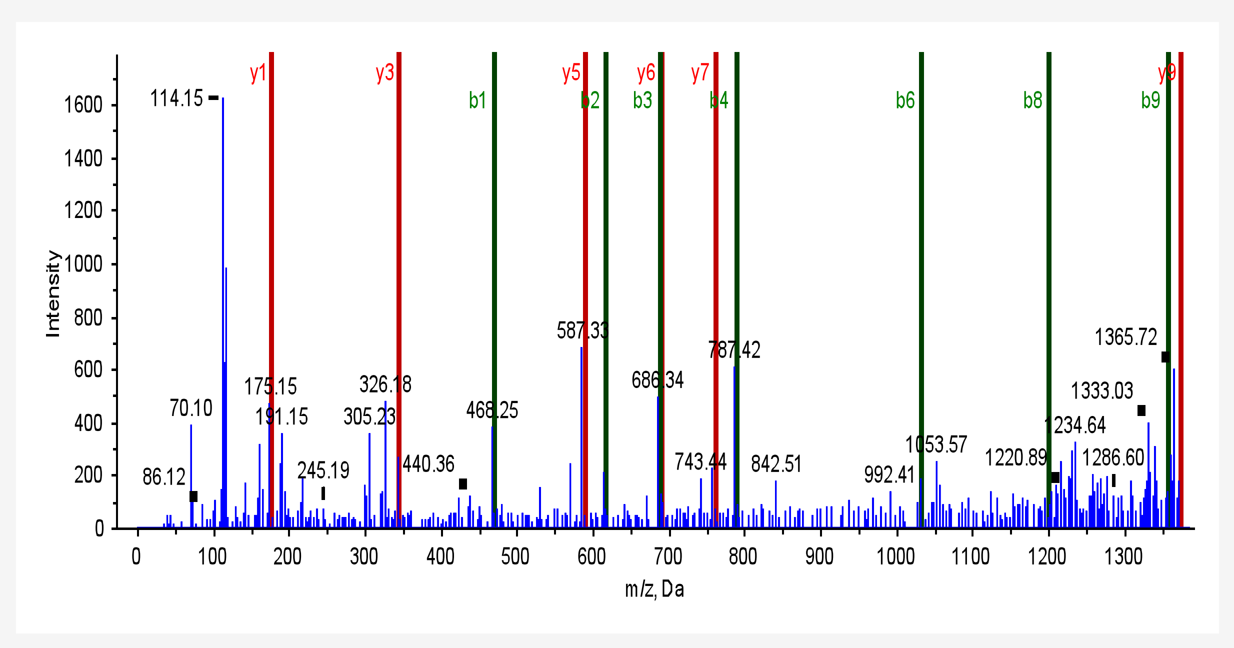

Residue	b	y
Y	468.28	1373.75
F	615.34	906.48
A	686.38	759.41
T	787.43	688.38
P	884.48	587.33
F	1031.55	490.28
P	1128.60	343.21
A	1199.64	246.16
R	1355.74	175.12

Competitor Protein: DVU2724_QAFRAR

m/z 893.4175 Deamidated@1;iTRAQ4plex@0; missed R-A@4 confidence 0.99

Primary ID: DVU1044A_EAPNYR

C14_PVDF_iTRAQ_Aldolase_3rd_10-10-07_(7_8_3).group

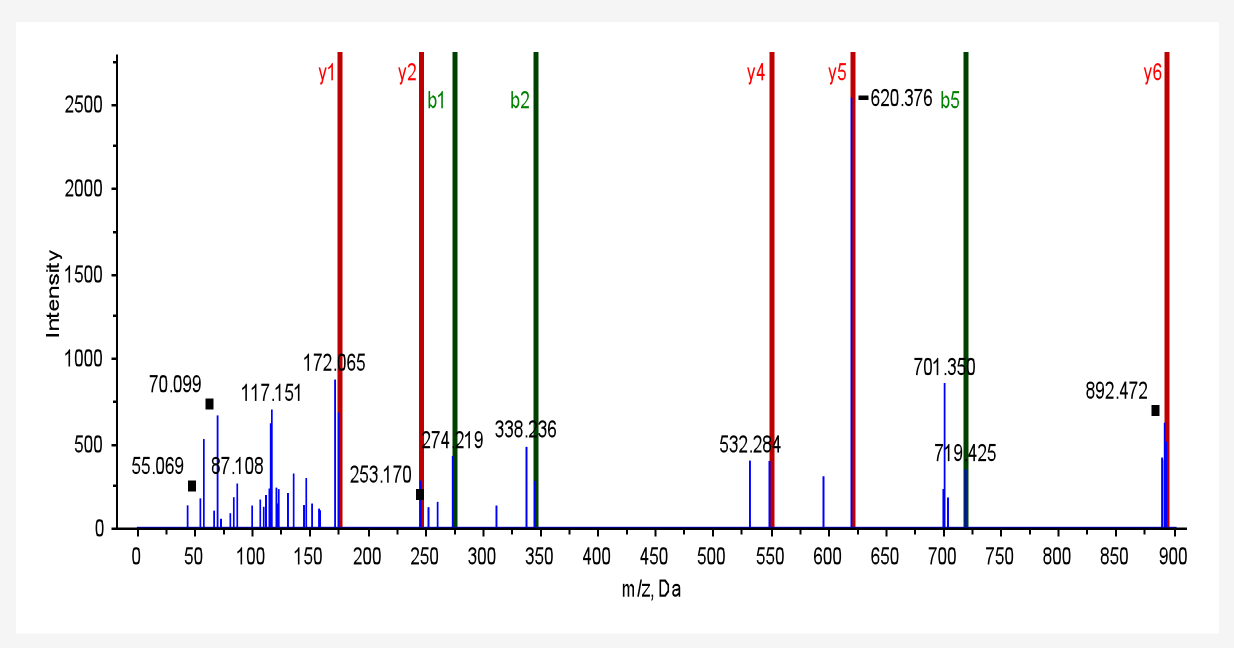

Residue	b	y
Q[Dea]	274.15	893.51
A	345.19	620.36
F	492.26	549.33
R	648.36	402.26
A	719.40	246.16
R	875.50	175.12

Competitor Protein: DVU2741_NLPYGMQR

m/z 1283.762 Deamidated@1;iTRAQ8plex@0; confidence 0.99

Primary ID: DVU0631_DLPPATPIR

S5_401-403_rerun_06082010_(2_3_5).group

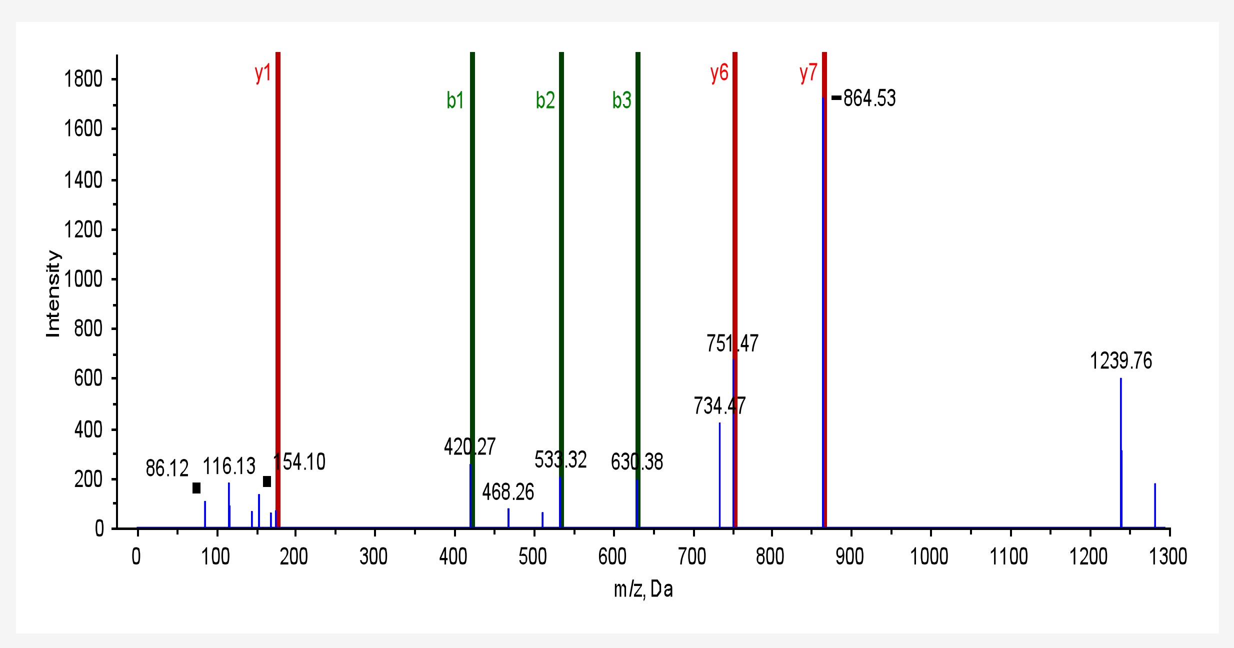

Residue	b	y
N[Dea]	420.24	1283.67
L	533.32	864.44
P	630.38	751.36
Y	793.44	654.30
G	850.46	491.24
M	981.50	434.22
Q	1109.56	303.18
R	1265.66	175.12

DVU2879_AGKFVADAHAALLR

m/z 2048.027 iTRAQ8plex@3;iTRAQ8plex@0; cleaved Q-A@N-term; missed K-F@3 confidence 0.99

AS2_601-603_03022012_(2_3_2).group

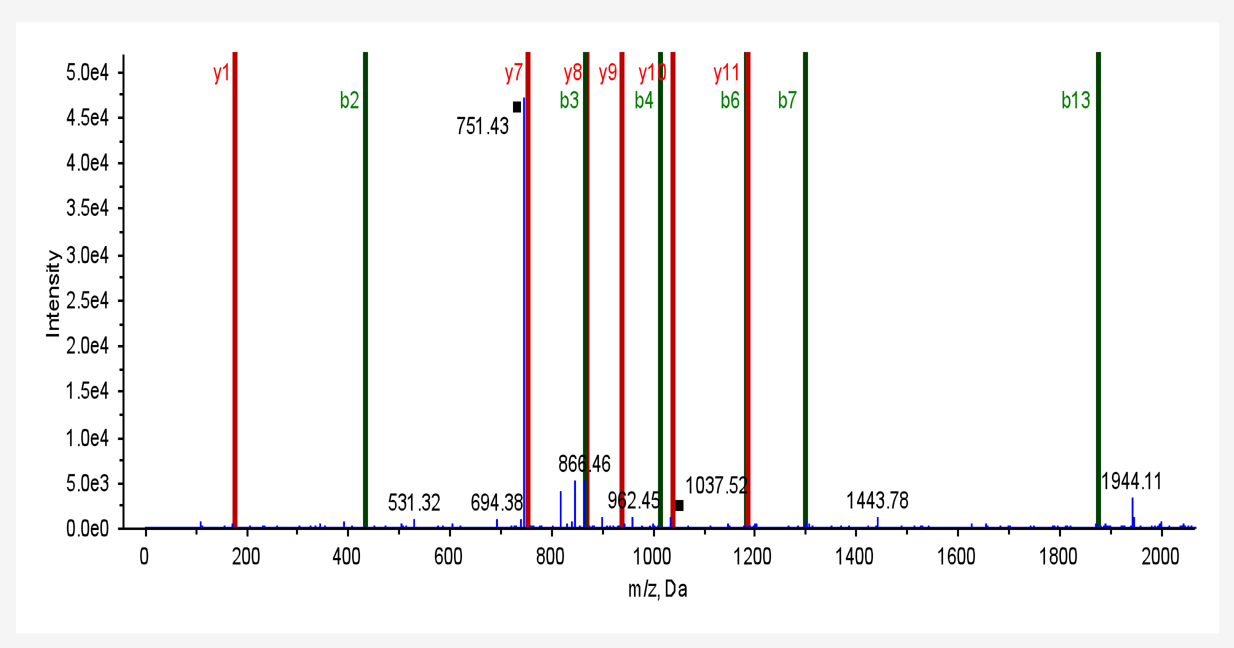

Residue	b	y
A	376.25	2048.22
G	433.27	1672.98
K[IT8]	865.57	1615.96
F	1012.64	1183.66
V	1111.71	1036.59
A	1182.75	937.52
D	1297.77	866.48
A	1368.81	751.46
H	1505.87	680.42
A	1576.91	543.36
A	1647.94	472.32
L	1761.03	401.29
L	1874.11	288.2
R	2030.21	175.12

Competitor Protein: DVU2967_IEELLR

m/z 1076.658 iTRAQ8plex@0; cleaved D-I@N-term confidence 0.99

Primary ID: DVU3260A_PLEELLR

S5_405-407_04232010_(2_3_7).group

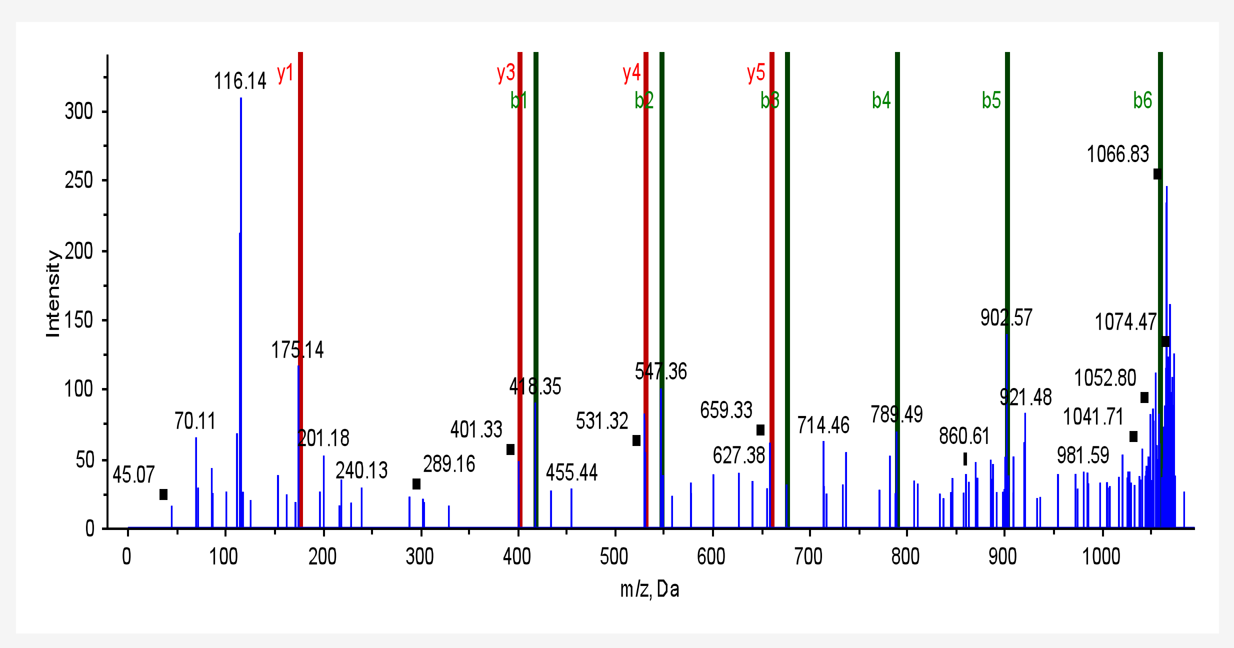

Residue	b	y
I	418.30	1076.66
E	547.34	659.37
E	676.38	530.33
L	789.47	401.29
L	902.55	288.20
R	1058.65	175.12

DVU2989_YAWPGNIR

m/z 1120.602 iTRAQ4plex@0; cleaved T-Y@N-term confidence 0.99

C14_PVDF_iTRAQ_Aldolase_3rd_10-10-07_(15_16_6).group

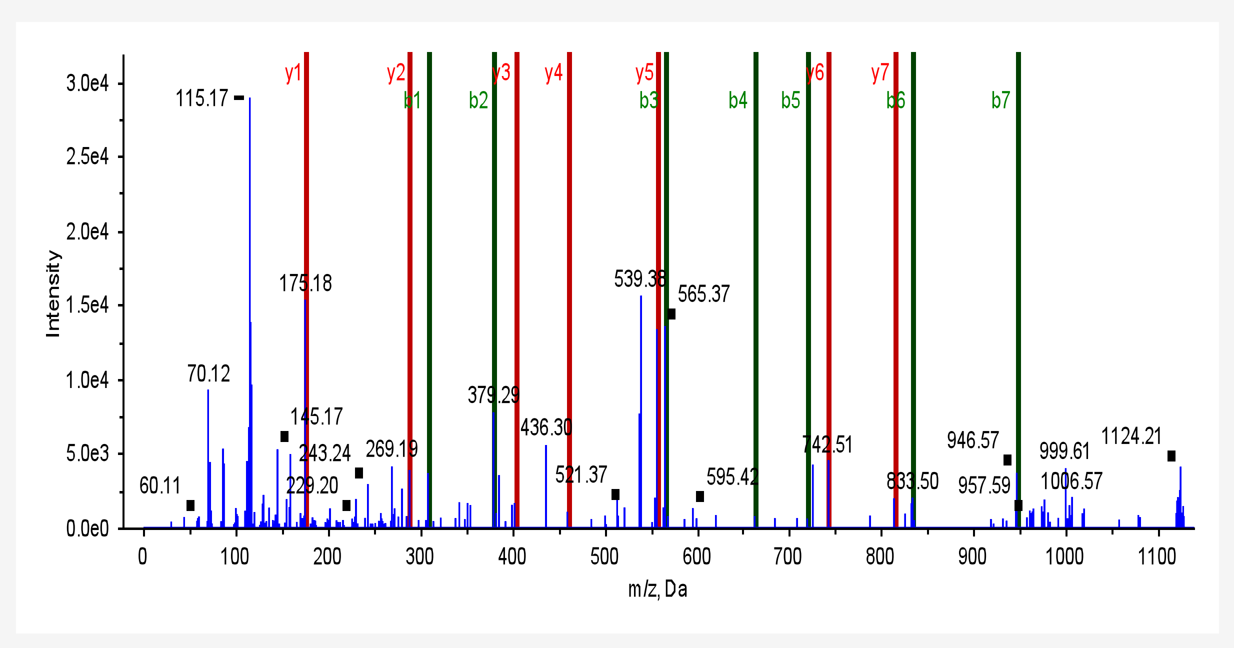

Residue	b	y
Y	308.17	1120.60
A	379.21	813.44
W	565.29	742.40
P	662.34	556.32
G	719.36	459.27
N	833.41	402.25
I	946.49	288.20
R	1102.59	175.12

DVU3022_IAPQVPVILVSGR

m/z 1652.966 iTRAQ8plex@0; cleaved D-I@N-term confidence 0.99

S5_619-621_08162012_(2_3_6).group

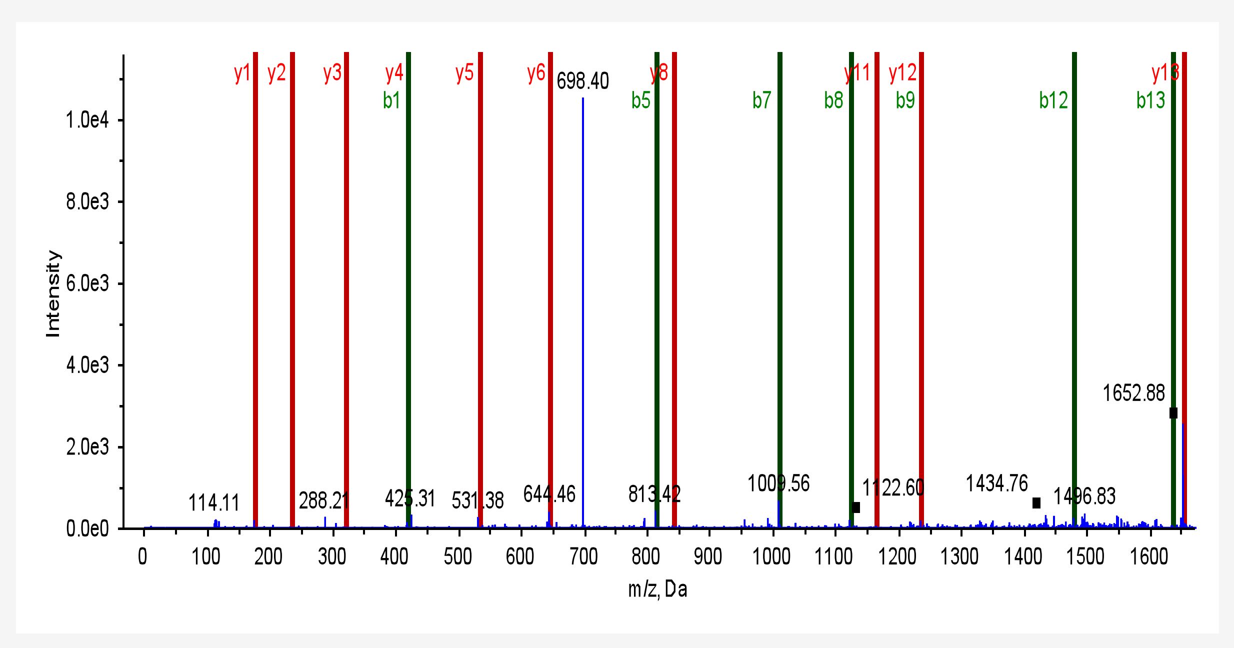

Residue	b	y
I	418.30	1653.04
A	489.33	1235.75
P	586.39	1164.71
Q	714.45	1067.66
V	813.51	939.60
P	910.57	840.53
V	1009.63	743.48
I	1122.72	644.41
L	1235.80	531.32
V	1334.87	418.24
S	1421.90	319.17
G	1478.92	232.14
R	1635.03	175.12

DVU3072_WQWAGLLPR

m/z 1447.115 Oxidation@8;iTRAQ8plex@0; confidence 0.99

S5_256_258_261_265_selected_4plexes_relabeled_092509_(2_3_2).group

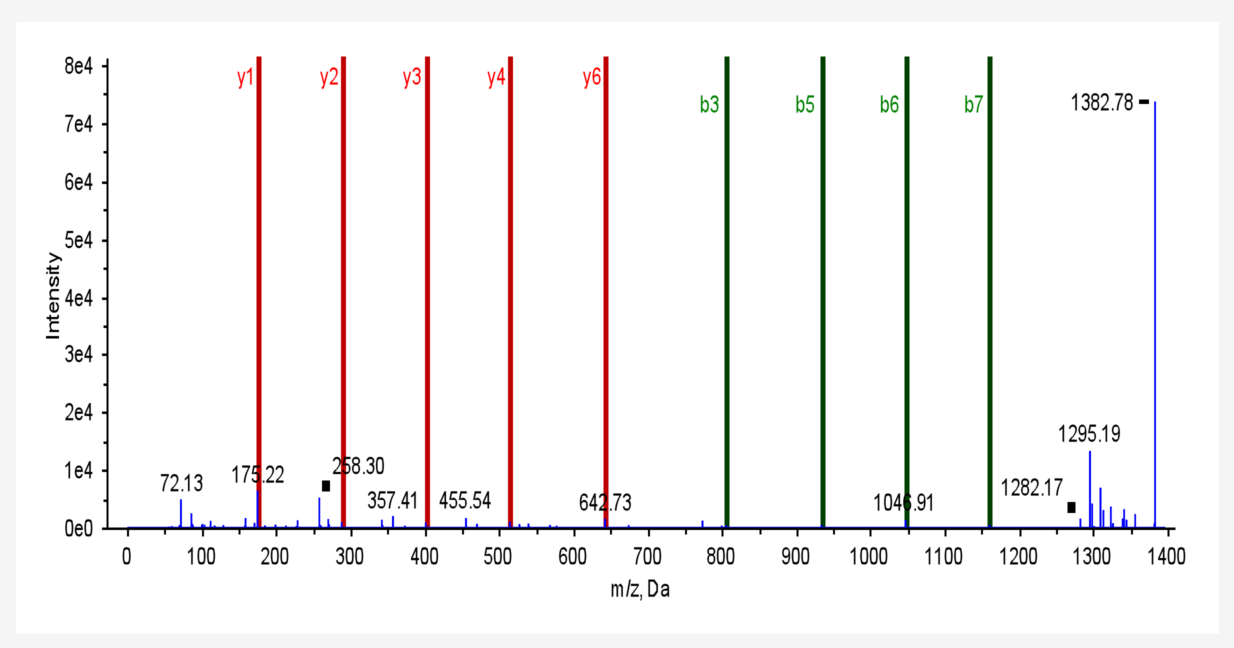

Residue	b	y
W	491.29	1446.82
Q	619.35	956.53
W	805.43	828.47
A	876.47	642.39
G	933.49	571.36
L	1046.57	514.33
L	1159.66	401.25
P[Oxi]	1272.70	288.17
R	1428.81	175.12

Competitor Protein: DVU3127_IEPER

m/z 947.6161 iTRAQ8plex@0; cleaved S-I@N-term confidence 0.99

Primary ID: DVU1833_LEILR

S5_376-378_8plex_1-3_correct_02-11-10_(2_3_9).group

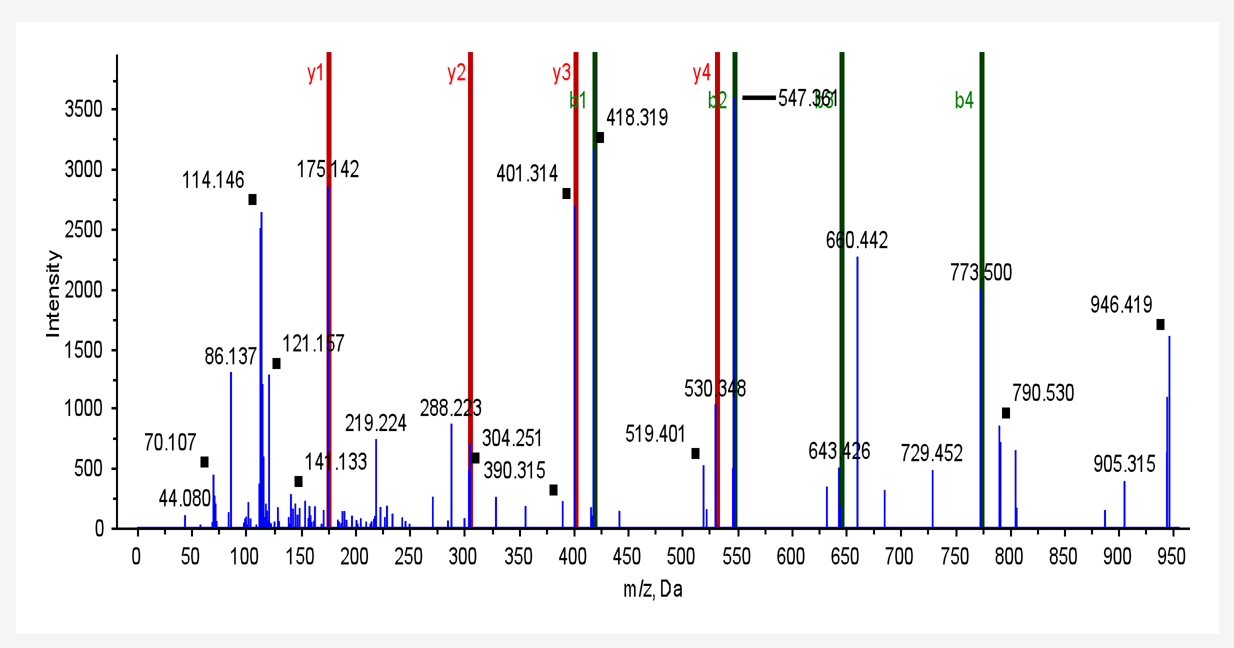

Residue	b	y
I	418.30	947.55
E	547.34	530.26
P	644.39	401.21
E	773.43	304.16
R	929.54	175.12

DVU3180_LSFQK

m/z 1230.773 iTRAQ8plex@5;iTRAQ8plex@0; cleaved S-L@N-term confidence 0.99

2B6_C2_C5_S5_256_258_04-28-09_(2_3_6).group

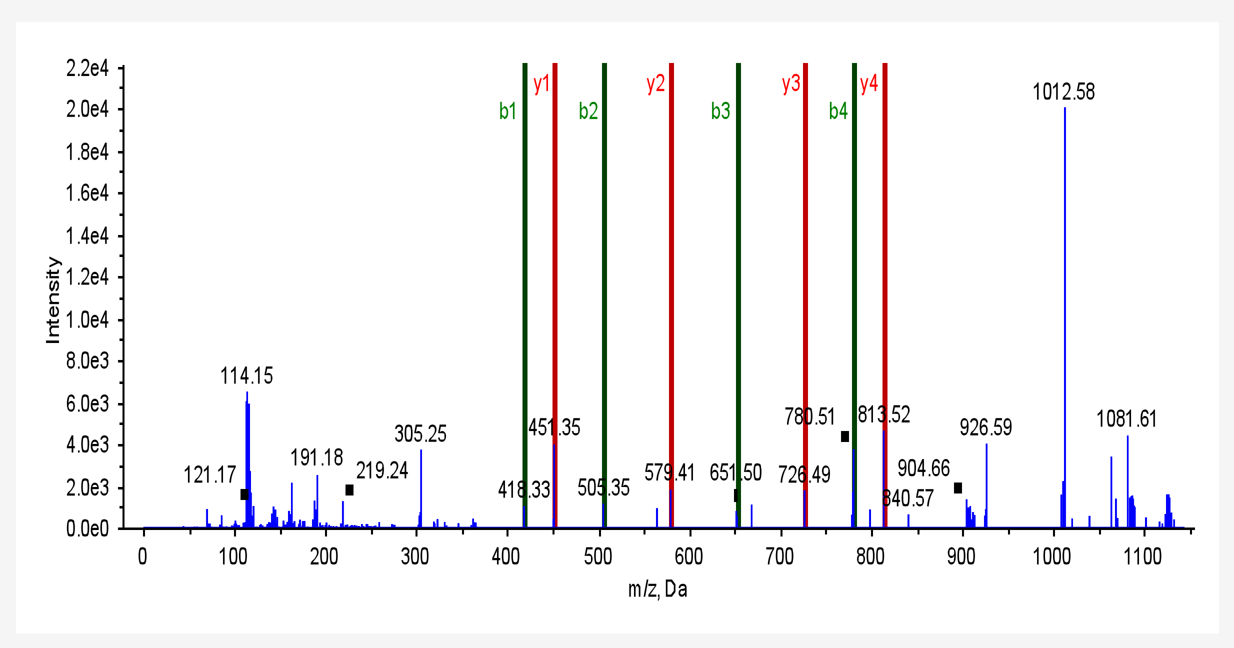

Residue	b	y
L	418.30	1230.77
S	505.33	813.48
F	652.40	726.45
Q	780.46	579.38
K[IT8]	1212.76	451.32

Competitor Protein: DVU3253_EVLDESER

m/z 1280.678 iTRAQ8plex@0; confidence 0.99

Primary ID: DVU3220_EVLDMVDR

2A6_C6_C8_DvH4-6_C10_C12_DvH2_5_6_rerun_03-31-09_(2_3_2).group

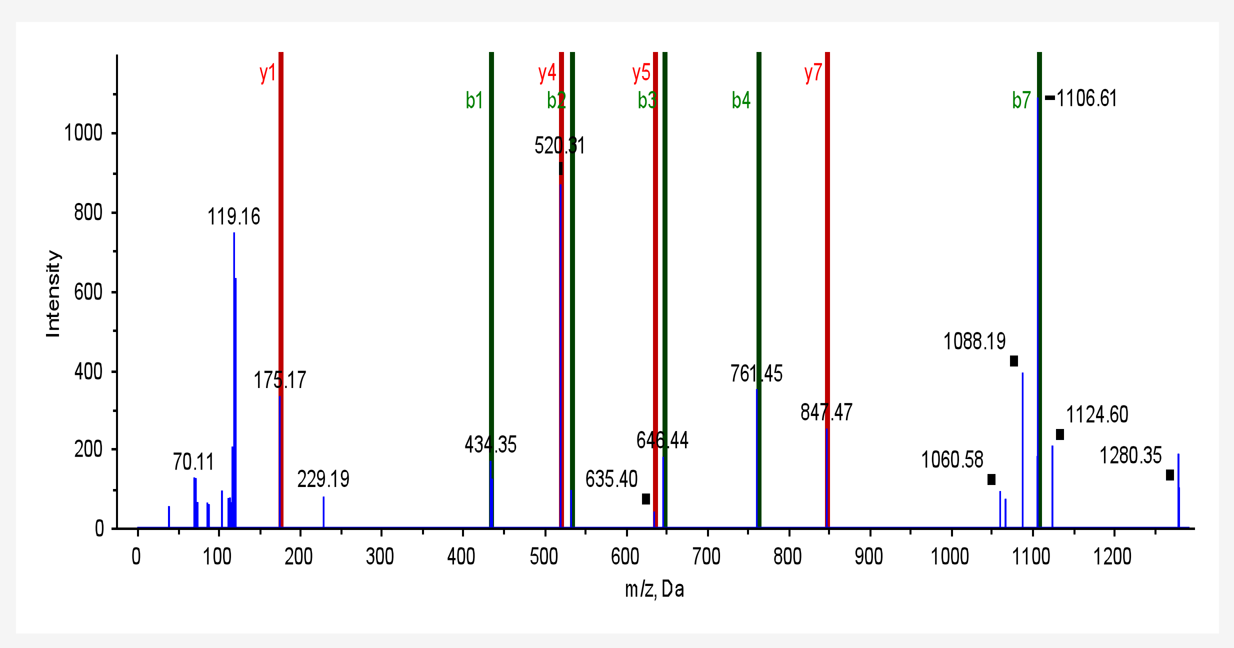

Residue	b	y
E	434.26	1280.66
V	533.32	847.42
L	646.41	748.35
D	761.43	635.26
E	890.48	520.24
S	977.51	391.19
E	1106.55	304.16
R	1262.65	175.12

Competitor Protein: DVU3388_LELVGGK

m/z 1323.839 iTRAQ8plex@7;iTRAQ8plex@0; confidence 0.9846

Primary ID: DVU1191_LELVNK

SEC_B15_C2_HIC_combos_B13-D7_090909_(2_3_3)_(3_4_3).group

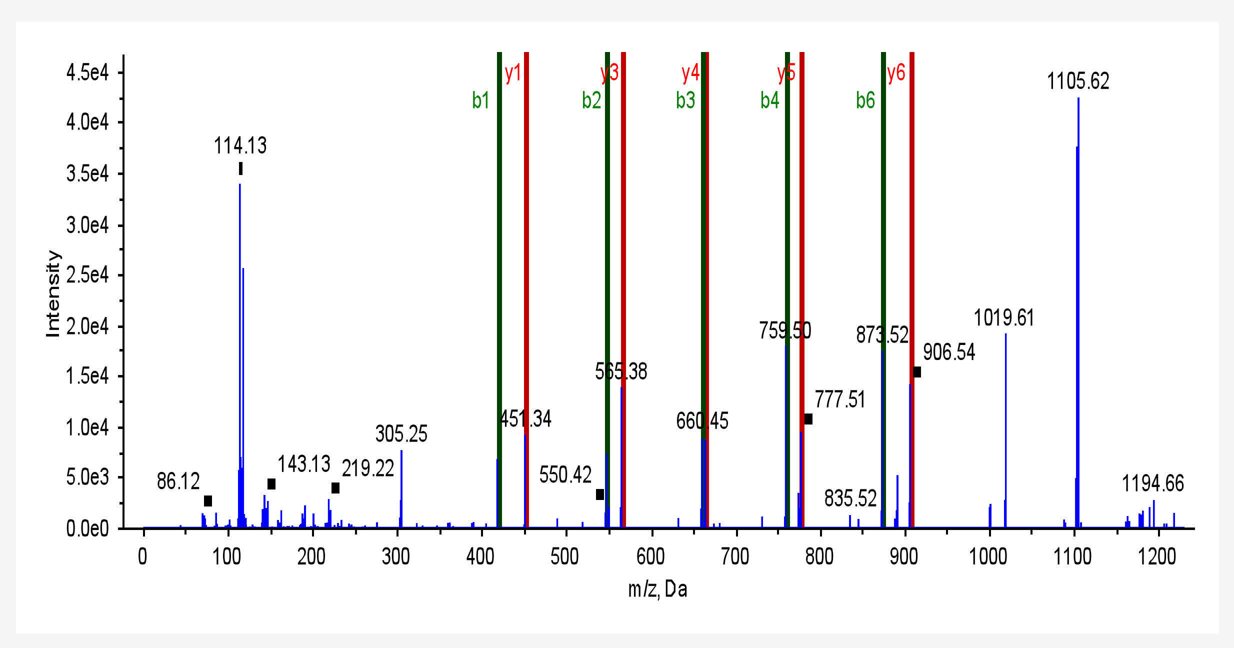

Residue	b	y
L	418.30	1323.85
E	547.34	906.56
L	660.42	777.51
V	759.49	664.43
G	816.51	565.36
G	873.53	508.34
K[IT8]	1305.84	451.32

Multiple IDs for V[L/I]A[L/I][L/I]R:

m/z 988.6803 iTRAQ8plex@0; semitryptic confidence 0.99

DVUA0013, DVU2885, DVU3167

S5_442-444_092110_(2_3_10)_(2_3_2)_(2_3_7).group

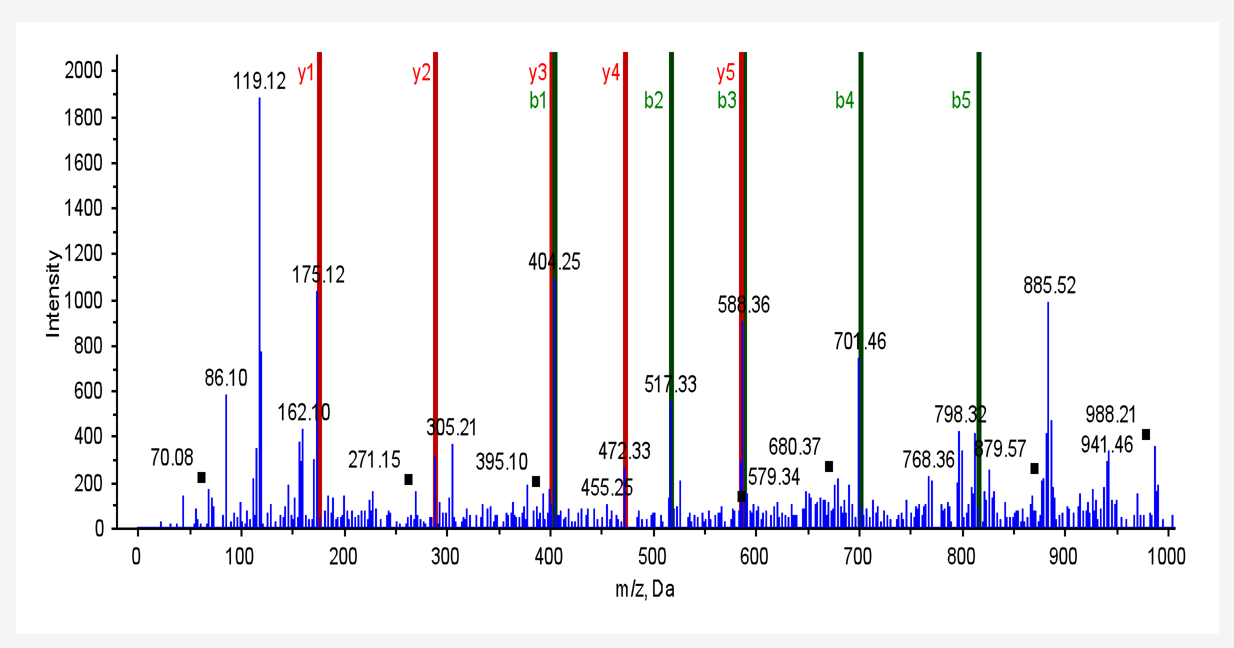

Residue	b	y
V	404.28	988.68
L/I	517.37	585.41
A	588.40	472.32
L/I	701.49	401.29
L/I	814.57	288.20
R	970.67	175.12

Competitor Protein: DVUA0039_LFYIR

m/z 1015.632 iTRAQ8plex@0; cleaved G-L@N-term; confidence 0.99

Primary ID: DVU3207_LFYIR

AS1_2C4_2D_SEC_B6-B12_(2_3_4).group

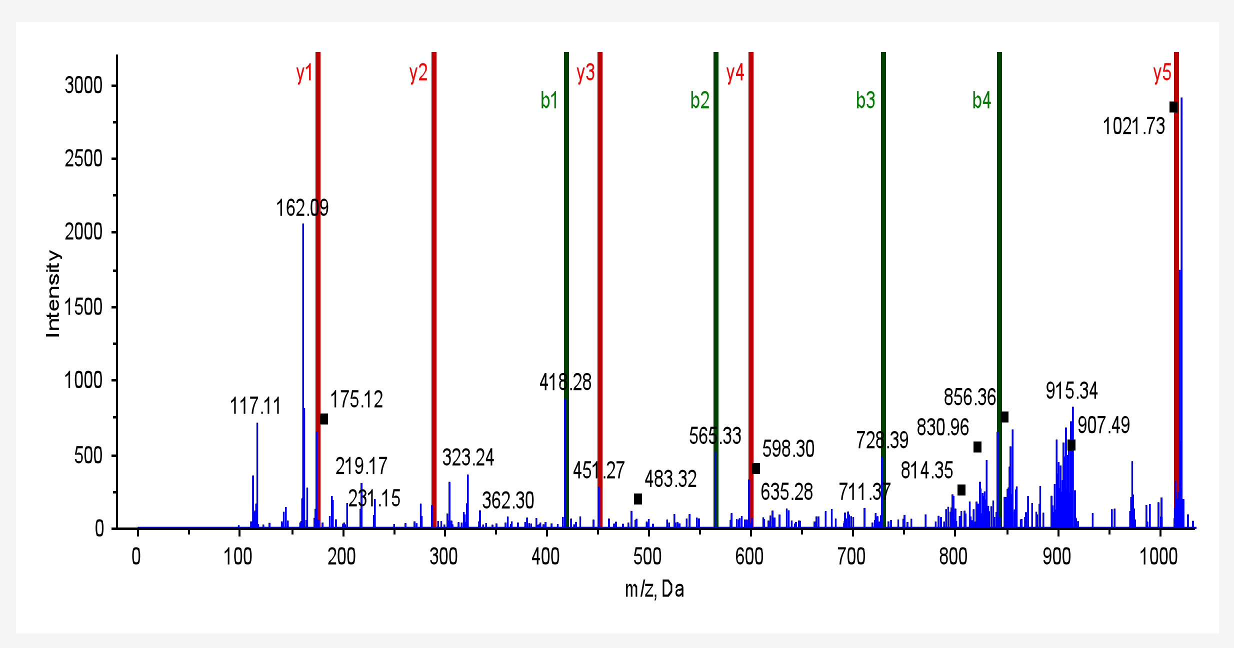

Residue	b	y
L	418.2967	1015.624
F	565.3651	598.3348
Y	728.4284	451.2663
I	841.5125	288.203
R	997.6136	175.119

Competitor Protein: DVUA0046_QEARGGK

m/z 1353.767 iTRAQ8plex@7;iTRAQ8plex@0; missed R-G@4 confidence 0.99

Primary ID: DVU1833_EQHFGK

S5_376-378_8plex_1-3_correct_02-11-10_(2_3_9).group

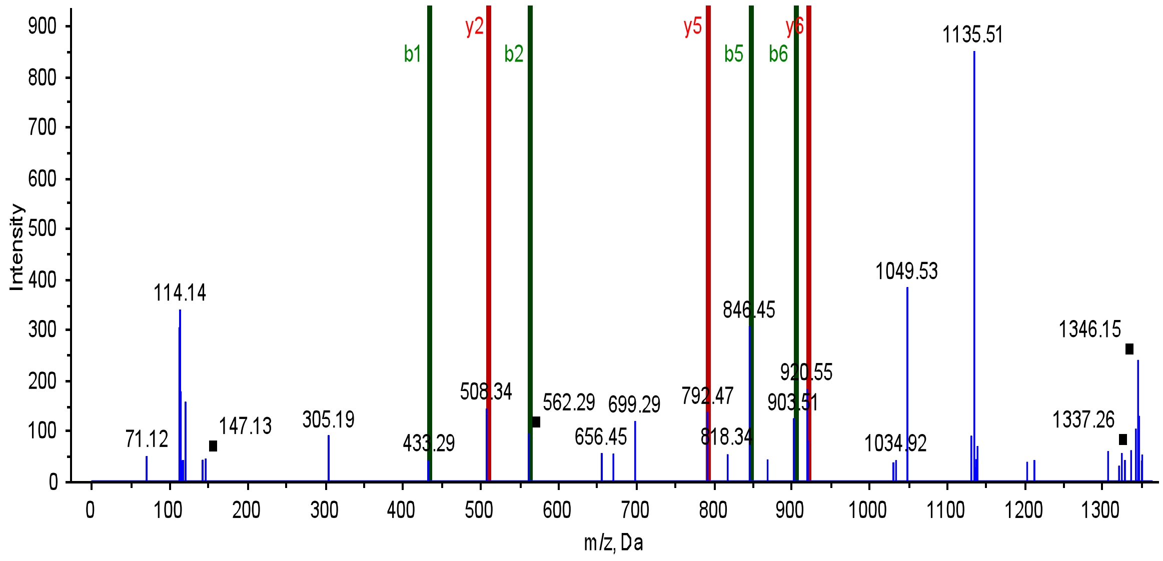

Residue	b	y
Q	433.27	1353.81
E	562.31	921.54
A	633.35	792.50
R	789.45	721.46
G	846.47	565.36
G	903.50	508.34
K[IT8]	1335.80	451.32

Competitor Protein: DVUA0086 _VFERAR

m/z 1081.63 iTRAQ8plex@0; missed R-A@4 confidence 0.99

Primary ID: DVU2376A _VFEINR

2C2_C10_C12_S5_333_331_082509_(3_4_4).group

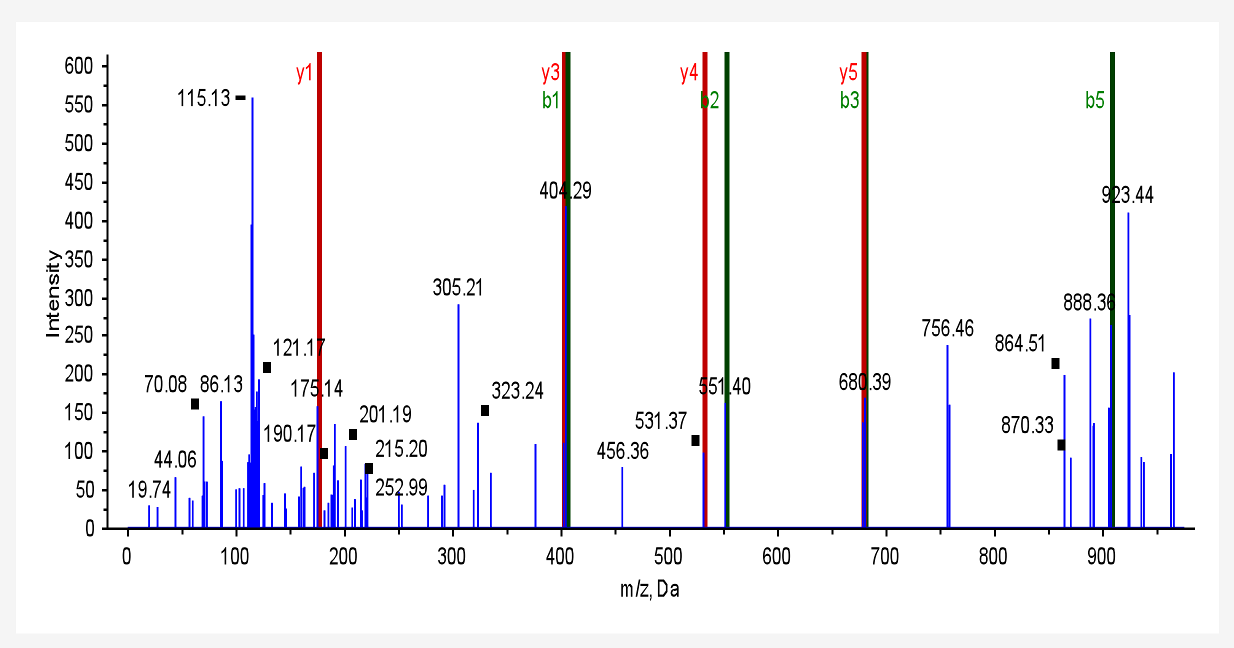

Residue	b	y
V	404.28	1081.64
F	551.35	678.37
E	680.39	531.30
R	836.49	402.26
A	907.53	246.16
R	1063.63	175.12
